# Simultaneous Occurrence of Magnetospheric Fluctuations at Different Discrete Frequencies ($f\approx $ 1 – 5 mHz): A Review

**DOI:** 10.1007/s11214-025-01166-6

**Published:** 2025-04-22

**Authors:** Simone Di Matteo, Umberto Villante

**Affiliations:** 1 Department of Physics, Catholic University of America, 620 Michigan Ave NE, Washington, 20064 DC USA; 2 Heliophysics Division, NASA - Goddard Space Flight Center, 8800 Greenbelt Rd, Greenbelt, 20771 MD USA; 3 Department of Physical and Chemical Science, University of L’Aquila, via Vetoio, Coppito, L’Aquila 67100 Italy; 4 Consorzio di Ricerca in Astrogeofisica, via Vetoio, Coppito, L’Aquila 67100 Italy

**Keywords:** ULF waves, “magic” frequencies, Solar wind “mesoscale” structures, MHD surface waves, Cavity/waveguide modes

## Abstract

In the last 30 years, many papers reported the almost simultaneous occurrence of magnetospheric fluctuations at different frequencies and latitudes (basically, in the range ${f}$ ≈ 1–5 mHz; ${T}$ ≈ 200–1000 s) and the possible existence and stability of sets of favorite frequencies (in particular: ${f}$$_{1}\approx $ 1.3, ${f}$$_{2}\approx $ 1.9, ${f}$$_{3}\approx $ 2.6–2.7, and ${f}$$_{4}\approx $ 3.2–3.4 mHz) has been proposed, determining controversial results. In the present paper we review these investigations focusing particular attention on several critical aspects that may have influenced the results and the comparison of these analyses (particularly, the correspondence between magnetospheric and solar wind fluctuations; the role of the short and long term variations of the solar wind and magnetospheric characteristics; the effects of the great variety of analytical methods adopted for the evaluation of power spectra and for the identification of relevant events). The results of this global analysis do not support the existence of a stable and persistent *absolute* set of favorite frequencies for magnetospheric oscillations; nevertheless, in the range of frequency explored by most investigations (${f}$ ≈ 1.5–4.0 mHz), they reveal a strong predominance of cases between ${f}$ ≈ 1.5–2.5 mHz, with percentages maximizing in the bin centered at ${f}$ = 2.0 mHz (a feature mostly due to events occurring at ${f}$ ≈ 1.9 mHz) and rapidly decreasing with increasing frequency; small evidence for an additional peak emerges at ${f}$ = 3.5 mHz; these aspects are much more explicit in the geomagnetic events than in the ionospheric and magnetospheric ones. Among other processes, the impact of the “mesoscale” solar wind density structures on the magnetosphere might be related with the onset of magnetospheric fluctuations at the observed frequencies.

**Supplementary Information:**

The online version contains supplementary material available at 10.1007/s11214-025-01166-6.

## Introduction

The first observation of rapid variations in the geomagnetic field elements (termed “pulsations” for a long time; $T\approx $ 0.1 – 600 s; $f\approx $ 10 Hz – 1.7 mHz; ultra-low-frequency waves, ULF) was made by Stewart (1861). Only a century later, Dungey (1954) proposed that the regular period of these fluctuations might be due to standing Alfvén waves excited on geomagnetic field lines and reflected between conjugate points of the ionosphere, due to its high conductivity (a process later termed “field line resonance, FLR”; Southwood 1974; Chen and Hasegawa 1974). In this scheme, for any given magnetic shell, identified by a particular value of the $L$-parameter ($L$ being roughly the geocentric equatorial distance of the field line expressed in Earth radii, $R_{E}$; McIlwain 1966), the wave eigenfrequency is determined by the field length between opposite hemispheres and by the distribution of the Alfvén velocity along it. Excited by external disturbances, the magnetospheric oscillations are set up in a narrow range of latitudes, with the wave peak amplitude occurring at the “resonant latitude” of the field line ($\lambda _{r}$; across $\lambda _{r}$ the phase of the wave is expected to change by ≈ 180°) that resonates at the frequency of the incoming wave (“resonance frequency”, $f_{r}$). In the magnetosphere, resonant oscillations (often consisting of several harmonics) occur mainly in the azimuthal direction (toroidal mode). The induced currents in the ionosphere rotate the FLR signal along the north-south component ($H$) of the ground-based magnetic field measurements; correspondingly, in the poloidal mode, the radial magnetic field perturbation is rotated along the east-west component ($D$) (Hughes and Southwood 1976). The observed ULF waves, in general, have a larger energy content at lower frequencies and exhibit an inverse relationship between the wave intensity and the frequency (Lanzerotti and Southwood 1979; Zong et al. 2017).

The ULF waves at the lowest frequencies, known as Pc5 waves ($T\approx $ 150 – 600 s; $f\approx $ 1.7 – 6.7 mHz), play an important role in the magnetosphere-ionosphere electrodynamics coupling and the energy transfer from the solar wind (SW) to the radiation belts and auroral regions, via modulation of particle precipitation (Wang et al. 2020) and total electron content (Kozyreva et al. 2020), Joule heating and/or ion frictional heating with neutrals (Hartinger et al. 2015), and geomagnetically induced currents (Heyns et al. 2021; Hartinger et al. 2023b). Waves in this frequency range are also relevant for processes related to the diffusion, loss and acceleration of particles in the radiation belts (Elkington 2006; Elkington and Sarris 2016; Zong et al. 2017); their periods, indeed, are comparable to those of the drift motion of the charged particles in the ring current and radiation belts. In this sense, Pc5 waves are also important in the context of *Space Weather*. Due to the increasing field line length, $f_{r}$ decreases with the increasing latitude; consequently, the fluctuations in the Pc5 range are commonly detected at high latitudes on closed magnetic field lines mapping in space from around the plasmapause to near the magnetopause on the dayside. Depending on the configuration of the plasmasphere, the latitudinal extension of these waves can vary, especially during geomagnetic storms when the waves often penetrate deeper into the magnetosphere (Lee et al. 2007; Degeling et al. 2018; Rubtsov et al. 2023). Since earliest investigations (Dungey 1955), the origin of the Pc5 waves has been mostly related to the Kelvin-Helmholtz instability (KHI), driven by large SW flow speed ($V_{SW}$), at the dawn/dusk flanks of the magnetopause. In this scheme, the magnetopause perturbations launch inward fast mode waves that, with suitable frequency, can couple into the shear Alfvén modes to form FLRs. Other possible sources have been proposed, such as the impulsive variations of the SW number density/dynamic pressure ($N_{SW}$/$P_{SW}$; $P_{SW} = N_{SW} V_{SW}^{2}$) and the impact on the magnetosphere of interplanetary shock waves (ISs), launching broadband waves, mostly in the noon region (Takahashi and Ukhorskiy 2007; Zong et al. 2009; Zhang et al. 2010; Liu et al. 2010). More in general, it is commonly accepted that ULF waves can be also driven externally by SW fluctuations, foreshock/bow shock phenomena, and magnetopause surface waves, and internally by magnetospheric plasma instabilities (Pu and Kivelson 1983; Sibeck et al. 1989; Agapitov et al. 2009; Keiling 2009; Claudepierre et al. 2010; Archer et al. 2019; Glassmeier et al. 1999; He et al. 2020). At middle and low latitudes, $f_{r}$ occurs in the range of the Pc4 ($T \approx $ 45 – 150 s; $f \approx $ 6.7 – 22.2 mHz) and Pc3 pulsations ($T \approx $ 10 – 45 s; $f \approx $ 22.2 – 100 mHz).

Within a vast bibliography dedicated to the Pc5 waves, a relevant number of papers, over thirty years, have analyzed the occurrence and the characteristics of magnetospheric waves more or less simultaneously occurring at different discrete frequencies (basically, in the range $f \approx $ 1 – 5 mHz; $T \approx $ 200 – 1000 s), the possible existence (and stability) of sets of favorite frequencies (identified as “CMS” or “magic” frequencies, see next paragraphs), and the relationships between the SW and the magnetospheric fluctuations at those frequencies. In the present review we focus on the papers that, in our opinion, are more closely related to these topics and pay particular attention to several critical aspects (so far mostly underestimated, in our opinion) that may have influenced the results (and the comparison) of these analyses, determining controversial results, for example:

i) the role of the $f_{r}$ variations and that of the solar cycle modulation on the organization and characteristics of the SW flow and magnetosphere, which highly question the stability of the “magic” frequencies.
ii) the intermixing and comparison of results obtained for different SW/magnetospheric structures and/or for different parameters, which necessarily mix different generation mechanisms.
iii) the effects of the great variety of the analytical methods adopted for the evaluation of the power spectra and those ones of the criteria used for the identification of the relevant events; the contamination from spurious results; the importance of the spacecraft position on the comparison of SW/magnetospheric measurements.

On the basis of the previous arguments, we chose to provide, for all the analyses of the magnetospheric oscillations examined in the present review, a detailed information of all parameters which are essential for a clear identification of the characteristics of each event and for a definite comparison of the experimental results obtained by different investigations (i.e., latitude, LT/MLT, phase of the solar cycle, radar/magnetic field measurements, statistical/individual analysis, method of analysis, SW structures impacting the magnetosphere, SW parameter compared with magnetospheric observations, etc.). These parameters, which are critical for a global analysis of the magnetospheric fluctuations at discrete frequencies, are often very difficult to recover; moreover, in many cases we needed to recalculate or deduce them from the available information. Nevertheless, to favor the reader less interested in such detailed information and provide a clear account of what has been so far achieved in this field of research, this information (never provided in previous papers) is also summarized in the Table 1.

**Table 1:** The characteristics of magnetospheric waves at discrete frequencies

#	Author(s)	<1.0	1.1–1.4	1.5–1.7	1.8–2.0	2.1–2.4	2.5–2.8	2.9–3.0	3.1–3.5	>3.5	UT	Data^1^	*f* range	*δf*	Location
		[mHz]	[mHz]	[mHz]	[mHz]	[mHz]	[mHz]	[mHz]	[mHz]	[mHz]	Date, Time		[mHz]	[mHz]	($\theta _{a}$, MLT)
[1]	Ruohoniemi et al. (1991)		1.3		1.9		2.6–2.9				1989/01/11 04:00-07:00	I	0.3–5.2	0.32	$\theta _{a}=69.1^{\circ}-70.7^{\circ}$ 00:20-03:20 MLT
[2]	Samson et al. (1991b)		1.3		1.9		2.6				1988/09/28 07:30-08:30	I	0.1–5.2	0.28	$\theta _{a}=72.5^{\circ}-73^{\circ}$ 03:50–04:50 MLT
			1.3		1.9						1988/09/29 04:15–05:15	I	0.1–5.2	0.28	$\theta _{a}=72.0^{\circ}-72.4^{\circ}$ 00:35–01:35 MLT
			1.3		1.9		2.6				1988/09/28 05:30–08:30	G	0.1–5.2	0.09	$\theta _{a}=68.7^{\circ}-73.7^{\circ}$ 22:30–01:30 MLT
			1.3		1.9						1988/09/29 04:00–05:00	G	0.3–5.2	0.28	$\theta _{a}=64.9^{\circ}-73.7^{\circ}$ 21:00–22:00 MLT
[3]	Samson et al. (1992a)		1.3		1.9		2.6–2.7		3.2–3.4		1988/09/28 1988/09/29 1988/10/02 1989/01/11 1989/12/07 1989/12/08 02:00–10:00	I	0.1–5.2	0.09	$\theta _{a}=69.0^{\circ}-71.5^{\circ}$ 22:20–06:20 MLT
[4]	Samson et al. (1992b)		1.3	1.6	1.9		2.6				1988/09/28 05:00–08:00	I	0.1–5.2	0.09	$\theta _{a}=72.5^{\circ}-73.0^{\circ}$ 01:30–04:30 MLT
			1.3		1.9				3.3		1989/12/08 05:30–08:30	I	0.1–5.2	0.09	$\theta _{a}=69.0^{\circ}-70.5^{\circ}$ 02:00–05:00 MLT
			1.3		1.9		2.7		3.4		1988/09/28 04:00–08:00	G	0.1–5.2	0.09	$\theta _{a}=68.7^{\circ}-73.7^{\circ}$ 21:00–01:00 MLT
			1.3		1.9				3.3		1989/12/08 05:30–08:30	G	0.1–5.2	0.09	$\theta _{a}=67.4^{\circ}-71.0^{\circ}$ 22:30–01:30 MLT
			1.2		1.8		2.6				1989/12/08 05:30–08:30	P^2^	0.1–5.2	0.09	$\theta _{a}\approx 68^{\circ}$ 22:30–01:30 MLT
[5]	Walker et al. (1992)	0.8	1.3		1.9		2.7		3.3		1989/01/11 04:15–07:55	I	0.1–5.2	0.04	$\theta _{a}=69.0^{\circ}-73.0^{\circ}$ 00:43–4:23 MLT
[6]	Boulet (1992)		1.3		1.9		2.6				1990	G^3^			
[7]	Samson and Rankin (1994)		1.3		1.8–1.9		2.7		3.3–3.4		1989/01/11 04:00–06:00	I	0.2–5.0	0.14	$\theta _{a}=69.0^{\circ}-71.5^{\circ}$ 00:30–02:30 MLT
[8]	Ziesolleck and McDiarmid (1994)				1.9				3.4	4.7	1993/01/01 12:30–14:30	G	1.0–6.0	0.14	$\theta _{a}=60.7^{\circ}-73.2^{\circ}$ 05:30–07:30 MLT
						2.2				4.0	1993/01/01 14:35–16:35	G	1.0–6.0	0.14	$\theta _{a}=60.7^{\circ}-73.2^{\circ}$ 07:35–09:35 MLT
					1.8		2.5				1993/01/01 16:30–18:30	G	1.0–6.0	0.14	$\theta _{a}=60.7^{\circ}-73.2^{\circ}$ 10:30–12:30 MLT
				1.5	1.9		2.6				1993/01/01 00:30–02:30	G	1.0–6.0	0.14	$\theta _{a}=60.7^{\circ}-73.2^{\circ}$ 17:30–19:30 MLT
[9]	Ziesolleck and McDiarmid (1995)		1.3		1.9		2.6		3.4		1993	G^3^	1.0–6.0	0.14	$\theta _{a}=67.4^{\circ}-73.3^{\circ}$ 00:00–24:00 MLT
			1.1		1.9	2.1		3.0		4.0	alternative sets of frequencies	G^3^			
				1.5		2.3		3.0		4.3		G^3^			
				1.5		2.3		3.0		3.7		G^3^			
[10]	Fenrich et al. (1995)		1.3		1.9						1988/09/28 07:30–08:50	I	0.3–5.0	0.31	$\theta _{a}=72.4^{\circ}-73.5^{\circ}$ 04:00–05:20 MLT
		1.0		1.6	1.9						1988/09/30 06:30–08:10	I	0.3–5.0	0.31	$\theta _{a}=73.5^{\circ}-74.0^{\circ}$ 03:00–04:40 MLT
		0.8	1.3		1.9		2.6		3.3		1989/01/01 05:50–07:00	I	0.3–5.0	0.31	$\theta _{a}=69.4^{\circ}-72.5^{\circ}$ 02:20–03:30 MLT
		0.8	1.3		1.9						1989/10/14 03:00–04:30	I	0.3–5.0	0.31	$\theta _{a}=69.4^{\circ}-70.4^{\circ}$ 23:30–01:00 MLT
			1.3	1.6	1.9		2.5			3.6	1993/10/06 22:00–23:40	I	0.3–5.0	0.31	$\theta _{a}=71.7^{\circ}-73.2^{\circ}$ 15:45–17:25 MLT
			1.3	1.5	1.9		2.5				1993/10/18 18:40–22:10	I	0.2–5.0	0.31	$\theta _{a}=72.5^{\circ}-72.8^{\circ}$ 12:25–15:55 MLT
					1.9						1993/11/18 15:50–17:30	I	0.3–5.0	0.31	$\theta _{a}\approx 68.2^{\circ}$ 09:35–11:15 MLT
					1.9						1993/12/16 03:20–04:50	I	0.3–5.0	0.31	$\theta _{a}\approx 70.2^{\circ}$ 21:05–22:35 MLT
			1.3								1993/12/29 00:00–01:40	I	0.3–5.0	0.31	$\theta _{a}\approx 73.3^{\circ}$ 17:45–19:25 MLT
							2.8				1994/01/11 13:20–15:00	I	0.3–5.0	0.31	$\theta _{a}\approx 69.8^{\circ}$ 07:05–08:45 MLT
					1.9		2.5–2.6				1994/02/02 00:00–01:50	I	0.3–5.0	0.31	$\theta _{a}=70.2^{\circ}-72.3^{\circ}$ 17:45–19:35 MLT
						2.2					1994/02/05 15:00–16:50	I	0.2–5.0	0.16	$\theta _{a}\approx 70.2^{\circ}$ 11:30–13:20 MLT
[11]	Shimazu et al. (1995)								3.3	4.7, 5.9, 7.1	1978/09/27 15:00–15:30	G	0.6–10.0	0.56	*θ* = 65.2^∘^ 18:04–18:34 MLT
												G	0.6–10.0	0.56	*θ* = 64.9^∘^ − 68.1^∘^ 03:27–07:00 MLT
[12]	Provan and Yeoman (1997)			1.6	1.8		2.4–2.8		3.2		1981–1989 01:45–02:45	I^3^	1.0–7.0	0.28	$\theta _{a}=61^{\circ}-67^{\circ}$ 02:00–03:10 MLT
[13]	Villante et al. (1997)		1.0–1.3		1.8–2.0			2.7–2.9	3.2–3.5	3.9–4.1 4.5–4.7	6 days, 00:00–24:00	G^3^	0.7–5.0	0.09	$\theta _{a}\approx 80^{\circ}$ S 00:00–24:00 MLT
[14]	Chisham and Orr (1997)					2.0–2.4			3.1	3.9, 4.9	≈1988	G^3^	1.6–6.0	0.2	$\theta _{a}=51.0^{\circ}-61.3^{\circ}$ 03:00–21:00 MLT
[15]	Francia and Villante (1997)		1.2–1.4		1.8–2.0		2.4–2.6			3.4–3.6 4.2	1985–1986 00:00-24:00	G^3^	0.3–5.0	0.09	$\theta _{a}\approx 36.2^{\circ}$ 00:00–24:00 MLT
		0.7			1.9						1989–1990 00:00-24:00	G^3^			
[16]	Prikryl et al. (1998)	0.8	1.3	1.7	2.0		2.4	2.9			1996/01/24 14:10–17:20	SW	0.8–4.0	0.13	
		0.8	1.1, 1.3	1.7	2.0		2.4	2.9				SW	0.8–4.0	0.13	
		0.8	1.1, 1.3	1.7				2.9			1996/01/24 14:30–17:40	MSH^2^	0.8–4.0	0.13	
		0.8/0.9	1.1	1.4, 1.6	1.9		2.4–2.5	3.0			1996/01/24 14:30–17:40	M	0.8–4.0	0.13	09:27–12:27 MLT 05:27–08:27 MLT
				1.3, 1.4	2.0		2.4				1996/01/24 14:30–17:40	I	0.8–4.0	0.13	*θ* = 70^∘^ − 77^∘^ 08:30–16:40 MLT
		0.8, 0.9	1.1	1.4, 1.6	1.9		2.4–2.5	3.0			1996/01/24 14:30–17:40	G	0.8–4.0	0.13	*θ* = 53.9^∘^ − 87.1^∘^ 03:40–15:18 MLT
[17]	Villante et al. (1998)	1.0	1.4		1.8	2.4					1997/01/11 00:00–04:00	G	0.8–5.0	0.07	$\theta _{a}\approx 36.2^{\circ}$ 01:40–05:40 MLT
		1.0	1.4		1.8	2.2					1997/01/11 00:00–04:00	G	0.8–5.0	0.07	$\theta _{a}\approx 80.0^{\circ}$ S 15:54–19:54 MLT
[18]	Lepidi et al. (1999)					2.1–2.2				3.6–3.7 4.7–4.8	1997/04/11 17:00–19:00	G	1.6–5.0	0.13	$\theta _{a}\approx 36.2^{\circ}$ 18:40–20:40 MLT
						2.1–2.2				3.6–3.7 4.7–4.8		G	1.6–5.0	0.13	$\theta _{a}\approx 80.0^{\circ}$ S 08:54–10:54 MLT
						2.1–2.2				3.6–3.7		M	1.6–5.0	0.13	12:00–14:00 MLT 08:00–10:00 MLT
[19]	Lessard et al. (1999)		1.4	1.7		2.1					1990/12/13 09:00–13:30	M	0.1–8.0	0.06	$\theta _{a}=63.0^{\circ}-65.4^{\circ}$ 00:00–04:00 MLT
						2.1					1990/12/13 10:00–13:30	M	0.1–8.0	0.08	03:10–06:40 MLT
			1.4	1.7		2.1					1990/12/13 08:00–13:00	G	1.0–5.0	0.06	$\theta _{a}=67^{\circ}-69^{\circ}$ 01:25–06:25 MLT
			1.3		1.8	2.1					1990/12/13 09:30–12:00	P^2^	0.5–8.0	0.11	$\theta _{a}=66^{\circ}-69^{\circ}$ 02:52–05:22 MLT
[20]	Mathie et al. (1999a)		1.2–1.4		1.8–2.0		2.4–2.6		3.2–3.4	4.0–4.2	1994/03, 1995/03, 1996/03	G^3^	1.0–5.0	0.18	$\theta _{a}=56.8^{\circ}-76.0^{\circ}$ 06:00–18:00 MLT
[21]	Villante et al. (2001)		1.1	1.7-1.8		2.3	2.8			3.7	1997–1998 00:00–24:00	G^3^	0.1–8.3	0.09	$\theta _{a}\approx 36.2^{\circ}$ 00:00–24:00 MLT
[22]	Stephenson and Walker (2002)		1.3		1.9	2.3	2.7		3.2–3.3		1997/04/28 20:00–06:00	SW	1.0–5.0	0.05	
			1.3		1.9	2.3	2.7		3.2–3.3			I	1.0–5.0	0.05	$\theta _{a}=66^{\circ}-70^{\circ}$ 21:25–04:55 MLT
[23]	Kepko et al. (2002)	0.7	1.4		2.0		2.7				2000/02/05 15:45–18:30	SW	0.1–4.0	0.07	
												M	0.1–4.0	0.07	06:30–09:15 MLT
		0.4, 0.7, 1.0	1.3								2000/04/27 01:52–05:00	SW	0.1–3.0	0.09	
												M	0.1–3.0	0.09	16:52–20:00 MLT
[24]	Kepko and Spence (2003)	0.2, 0.6		1.7							1999/06/15 14:10–16:55 12:18–21:06	SW	0.1–5.0	0.1	
												M	0.1–2.0	0.03	09:10–11:55 MLT 07:18–16:06 MLT
		0.3, 0.7	1.2–1.3	1.7							1997/02/04 12:30–22:30	SW	0.1–3.0	0.03	
												M	0.1–3.0	0.03	07:30–17:30 MLT
		0.6	1.3		1.9	2.4					1996/11/29 15:00–17:00	SW	0.2–5.0	0.14	
												M	0.2–5.0	0.14	10:15–12:15 MLT
		0.8		1.5							1997/09/21 16:10–18:10	SW	0.2–5.0	0.14	
												M	0.2–5.0	0.14	11:18–13:18 MLT
		0.6, 1.0	1.3		1.8						1998/08/03 14:00–18:10	SW	0.1–5.0	0.07	
												M	0.1–5.0	0.07	09:00–13:10 MLT
[25]	Baker (2003)	continuous distribution									1989–1999 00:00–24:00	G^3^	1.7–6.7	0.17	$\theta _{a}=61.2^{\circ}-73.7^{\circ}$ 00:00–24:00 MLT
[26]	Eriksson et al. (2006a)	0.8–1.4			2.0–2.6						2000/04/18 2000/04/19	I, SW	0.8–4.4	0.08	$\theta _{a}=67.7^{\circ }S-69.4^{\circ }S$
											2001/02/12	I, SW	0.8–4.4	0.08	$\theta _{a}=65.6^{\circ }S-67.2^{\circ }S$
											2001/04/24 2001/04/25	I, SW	0.8–4.4	0.08	$\theta _{a}=67.4^{\circ }S-69.9^{\circ }S$
											2001/04/30	I, SW	0.8–4.4	0.08	$\theta _{a}=68.1^{\circ }S-70.9^{\circ }S$
											2001/05/04 2001/05/05	I, SW	0.8–4.4	0.08	$\theta _{a}=66.7^{\circ }S-69.9^{\circ }S$
											2001/06/09	I, SW	0.8–4.4	0.08	$\theta _{a}=66.9^{\circ }S-69.4^{\circ }S$
											2001/09/17	I, SW	0.8–4.4	0.08	$\theta _{a}=66.7^{\circ }S-68.8^{\circ }S$
[27]	Villante et al. (2006)		1.4				2.2			4.2	2004/04/03 13:14–14:14	SW	0.3–8.0	0.28	
			1.4					3.0		4.2		SW	0.3–8.0	0.28	
		0.8						3.0		4.0		SW	0.3–8.0	0.28	
			1.4				2.2–2.5			4.2	2004/04/03 14:18–15:18	G	0.3–8.0	0.28	$\theta _{a}=33.4^{\circ}-42.8^{\circ}$ 15:00–16:00 MLT
[28]	Villante et al. (2007)		1.3			2.2			3.2		1998/08/01 17:30–19:30	SW	0.7–5.0	0.14	
												M	0.7–5.0	0.14	12:30–14:30 MLT 09:45–11:45 MLT
												G	0.7–5.0	0.14	$\theta _{a}\approx 36.2^{\circ}$ 19:07–21:07 MLT
		1.0									1998/08/01 19:30–21:30	SW	0.7–5.0	0.14	
												M	0.7–5.0	0.14	14:30–16:30 MLT 11:54–13:54 MLT
												G	0.7–5.0	0.14	$\theta _{a}\approx 36.2^{\circ}$ 21:07–23:07 MLT
						2.1			3.5		2000/06/08 12:30–14:30	SW	0.7–5.0	0.14	
												M	0.7–5.0	0.14	07:15–09:15 MLT 03:10–05:10 MLT
												G	0.7–5.0	0.14	$\theta _{a}\approx 36.2^{\circ}$ 14:07–16:07 MLT
		1.0				2.3	2.7				2000/11/07 19:30–21:30	G	0.7–5.0	0.14	$\theta _{a}\approx 36.2^{\circ}$ 21:07–23:07 MLT
			1.2				2.6	2.9			2001/04/13 13:30–15:30	G	0.7–5.0	0.14	$\theta _{a}\approx 36.2^{\circ}$ 15:07–17:07 MLT
		1.0									2001/06/26 19:30–21:30	G	0.7–5.0	0.14	$\theta _{a}\approx 36.2^{\circ}$ 21:07–23:07 MLT
		0.9	1.4								2001/09/18 17:00–19:00	G	0.7–5.0	0.14	$\theta _{a}\approx 36.2^{\circ}$ 18:37–20:37 MLT
						2.2	2.7				2001/11/05 15:00–17:00	G	0.7–5.0	0.14	$\theta _{a}\approx 36.2^{\circ}$ 16:37–18:37 MLT
							2.5			4.0, 4.5	2002/03/20 15:15–17:15	M	0.7–5.0	0.14	10:10–12:10 MLT 06:00–08:00 MLT
												G	0.7–5.0	0.14	$\theta _{a}\approx 36.2^{\circ}$ 16:52–18:52 MLT
[29]	Lee et al. (2007)						2.8				1991/03/24 08:15–09:15	G	1.0–6.0	0.28	$\theta _{a}=58^{\circ}-74^{\circ}$ 05:26–12:24 MLT 12:49–16:49 MLT
					1.9						1991/03/24 10:10–11:10	G	1.0–6.0	0.28	$\theta _{a}=60^{\circ}$ 07:21–14:19 MLT
				1.7							1991/03/24 12:00–13:40	G	1.0–6.0	0.28	$\theta _{a}=61^{\circ}$ 12:49–16:49 MLT
[30]	Liou et al. (2008)				1.8					3.5, 4.8, 7.7	1999/09/26 19:00–20:42	UVI^2^	0.2–13.6	0.16	*θ* = 60^∘^ − 90^∘^ 02:00–11:00 MLT
					2.0							SW			
				1.5								PSH^2^			footpoint *θ* = 65.2^∘^ 02:36 MLT
					2.0					3.0-4.0		G			*θ* = 29.4^∘^ − 70.3^∘^ 03:56–06:16 MLT
[31]	Viall et al. (2009)	0.7	1.4		2.0					4.8	1995–2005	SW^3^	0.5–5.0	0.05	
		1.0		1.5	1.9		2.8		3.3	4.4		M^3^	0.5–5.0	0.05	05:00–19:00 LT
[32]	Plaschke et al. (2009a)		1.3		1.9		2.5		3.1	4.1	2007/02 - 2007/09	M^3^			
[33]	Mthembu et al. (2009)	0.6	1.3	1.5	1.9						2002/11/11 06:00–08:00	I	0.2–4.0	0.14	$\theta _{a}=68.5^{\circ}-74.0^{\circ}$ 02:20–04:20 MLT
[34]	Stephenson and Walker (2010)				1.9	2.1					2000/06/07 01:00–20:00	SW	1.5–4.0	0.15	
					1.9	2.1					2000/06/07 02:30–19:00	I	1.5–4.0	0.17	$\theta _{a}\approx 66^{\circ }S$ 00:45–17:15 MLT
[35]	Kokubun (2013)				1.9		2.5		3.3	4.5	1995–2000	M^3^	0.3–10.0	0.32	
					1.9		2.6				1999/02/09 12:35–13:27	M	0.3–10.0	0.32	
												G	0.3–8.3	0.32	$\theta _{a}=72.3^{\circ}-78.6^{\circ}$ 05:26–07:26 MLT 12:33–17:03 MLT
						2.2			3.1	3.8	1999/05/19 13:50–14:42	M	0.3–10.0	0.32	
												G	0.3–8.3	0.32	$\theta _{a}=65.2^{\circ}-73.5^{\circ}$ 07:02–10:30 MLT 11:38–17:42 MLT
				1.6			2.6		3.2	4.2–4.3	1999/03/07 13:35–16:37	M	0.3–10.0	0.32	
												G	0.3–8.3	0.32	$\theta _{a}=66.8^{\circ}-78.7^{\circ}$ 06:47–14:07 MLT
						2.2			3.2	3.8, 4.2	1999/05/14 04:20–06:50	M	0.3–10.0	0.32	
												G	0.3–8.3	0.32	
						2.1		2.9	3.4	3.8	1999/08/20 17:20–18:12	M	0.3–10.0	0.32	
												G	0.3–8.3	0.32	$\theta _{a}=65.2^{\circ}-78.6^{\circ}$ 10:35–14:03 MLT 14:53–16:33 MLT
				1.6					3.5		1999/12/13 17:25–18:17	M	0.3–10.0	0.32	
												G	0.3–8.3	0.32	$\theta _{a}=66.1^{\circ}-78.6^{\circ}$ 11:37–15:47 MLT 20:01–21:41 MLT
[36]	Villante et al. (2013)		1.0		1.9	2.1–2.2	2.7–2.8		3.4–3.6	4.6–5.0	2001/03/28 02:30–04:30	SW	0.3–8.0	0.14	
						2.4			3.2	4.2		M	0.3–8.0	0.14	21:20–23:20 MLT
				1.6			2.5–2.7			3.5–3.7		M	0.3–8.0	0.14	17:20–19:20 MLT
		0.8–1.0					2.7–2.9			4.7		G	0.3–8.0	0.14	$|\theta _{a}|\approx 70^{\circ}-83^{\circ}$ 13:00–22:00 MLT
					1.9		2.6		3.4–3.6	4.7		G	0.3–8.0	0.14	$|\theta _{a}|<70^{\circ}$ 13:00–22:00 MLT
		1.0				2.2–2.4				4.7		G	0.3–8.0	0.14	$|\theta _{a}| \approx 48^{\circ}-65^{\circ}$ 03:30–11:30 MLT
		1.0			1.9	2.2–2.4	2.6–2.7		3.5–3.6	4.7		G	0.3–8.0	0.14	$|\theta _{a}|<48^{\circ}$ 03:30–11:30 MLT
						2.1–2.4	2.6			3.5–3.9 4.7		G	0.3–8.0	0.14	$\theta _{a} =30^{\circ }S-85^{\circ}$ 23:00–02:00 MLT
[37]	Archer et al. (2013)	0.7	1.1								2008/06/18-2008/10/06	M^3^	0.2–167	0.14	
												M^3^	0.2–167	0.14	10:30–13:30 MLT
[38]	Archer and Plaschke (2015)	0.6	1.3		1.9		2.6		3.2		2001 - 2013	M^3^			
[39]	Norouzi-Sedeh et al. (2015)			1.6		2.1		2.9	3.3	4.0–6.0	2006/01 - 2009/06	I^3^	0.5–30.0	0.17	$\theta _{a}=55^{\circ }S-85^{\circ }S$ 13:00–23:00 MLT
		0.8	1.2	1.7		2.1	2.6		3.1			G^3^	0.5–30.0	0.09	$\theta _{a}\approx 64.3^{\circ }S$ 09:30–21:30 MLT
[40]	Villante et al. (2016)			1.5–1.8					3.1–3.3	4.1–4.6 5.9–6.1	2001/04/04 14:25–15:33	SW	0.5–7.7	0.52	
				1.5–1.8					3.3–3.6		2001/04/04 14:58–16:06	M	0.5–7.7	0.52	09:58–11:06 MLT
			1.3–1.8						3.3–3.6	5.9–6.4		M	0.5–7.7	0.52	05:58–07:06 MLT
				1.5–1.8					3.3–3.6	4.4–4.6 5.9–6.2		G	0.5–7.7	0.52	$\theta _{a}=64.4^{\circ }S-73.9^{\circ}$ 00:00–24:00 MLT
[41]	De Lauretis et al. (2016)				2.0						2013/03/27 18:00–19:00 20:45–21:45	SW	1.0–5.0	0.28	
					2.0				3.0–4.0			I	1.0–7.0	0.28	$\theta _{a}\approx 76^{\circ }S$ 06:00–07:00 MLT $\theta _{a}\approx 75^{\circ }S$ 09:45–10:45 MLT
					2.0				3.3	3.9		G	1.0–7.0	0.28	$\theta _{a}\approx 80.0^{\circ }S$ 10:00–11:00 MLT 12:45–13:45 MLT
[42]	Klibanova et al. (2016)							2.9		4.4–4.7	2012/07/14 18:00–18:30	SW	1.7–6.7	0.56	
								2.9		4.4–4.7		G	1.7–6.7	0.56	$\theta _{a}\approx 14.6^{\circ}-82.6^{\circ}$ 06:40–22:05 MLT
[43]	Di Matteo and Villante (2017)				1.7–1.9		2.7–3.4			3.9–4.4	1998–2008	SW^3^	1.2–4.9	0.24	
[44]	Di Matteo and Villante (2018)			1.5–1.7		2.2–2.4				3.9–4.2 4.2–4.7	1998–2008	M^3^	1.2–4.9	0.24	01:00–23:00 MLT
				1.5	1.9		2.7			3.7–3.9 4.4–4.7	2002/11/09 18:40–20:10	M	1.2–4.9	0.24	13:50–15:20 MLT 09:50–11:20 MLT
					1.7–1.9	2.2		3.4		4.2–4.4	2001/02/12 21:20–22:50	M	1.2–4.9	0.24	16:00–17:30 MLT 12:00–13:30 MLT
[45]	Shi et al. (2018)	almost continuous distribution (with a most probable frequency at *f*≈2.08 mHz)									2010–2016	I^3^	1.7–40.0	0.07	$\theta _{a}=55^{\circ}-88^{\circ}$ 00:00–24:00 MLT
[46]	Archer et al. (2019)			1.7	1.8				3.3		2007/08/07 22:10–22:50	M	0.4–10.0	0.42	
									3.5	3.9		G	0.5–8.0	0.2	$\theta _{a}\approx 65.5^{\circ}$ 08:21–09:00 MLT
[47]	He et al. (2020)		1.4–1.5								2017/07/16 13:40:14:40	M	0.28–5.0	0.28	*L* ≈ 4.2 − −5.5 15:40–16:40 MLT
			1.4								2017/07/16 13:30–14:30	M	0.28–5.0	0.28	*L* ≈ 3.0 − −4.2 18:25–19:25 MLT
		0.6	1.1, 1.4		2.0						2017/07/16 12:30–14:30	G	0.2–10.0	0.14	$\theta _{a}\approx 61^{\circ}-75^{\circ}$ 15:40–17:40 MLT
[48]	Di Matteo et al. (2022)	0.2									2002/11/09 18:27–19:49	SW	1.1–4.1	0.55	
		0.2									2002/11/09 19:49–21:19	SW	1.1–4.1	0.55	
		0.2					2.6				2002/11/09 21:19–22:43	SW	1.1–4.1	0.55	
		0.2									2002/11/09 22:43–01:46	SW	1.1–4.1	0.55	
		0.2		1.6							2002/11/09 18:52–20:14	M	1.1–7.2	0.55	14:02–15:20 MLT
		0.2				2.3				3.6, 4.5	2002/11/09 20:14–21:44	M	1.1–7.2	0.55	15:20–16:50 MLT
		0.2					2.6	3.0	3.4		2002/11/09 21:44–23:08	M	1.1–7.2	0.55	16:50–18:10 MLT
		0.2			1.9	2.4					2002/11/09 23:08–02:55	M	1.1–7.2	0.55	18:10–22:00 MLT
		0.2		1.6						4.6	2002/11/09 18:52–20:14	M	1.1–7.2	0.55	10:02–11:20 MLT
		0.2				2.4					2002/11/09 20:14–21:44	M	1.1–7.2	0.55	11:20–12:50 MLT
		0.2					2.7				2002/11/09 21:44–23:08	M	1.1–7.2	0.55	12:50–14:10 MLT
		0.2			1.9				3.2		2002/11/09 23:08–02:55	M	1.1–7.2	0.55	14:10–18:00 MLT
		0.2		1.5						3.7, 4.6	2002/11/09 19:07–20:38	G	1.1–7.2	0.55	$\theta _{a}\approx 50^{\circ}-80^{\circ}$ 07:00–22:00 MLT
		0.2				2.3				3.4–3.7	2002/11/09 19:45–21:16	G	1.1–7.2	0.55	$|\theta _{a}| \gtrsim 30^{\circ}$ 12:00–24:00 MLT
		0.2				2.4–2.6		2.9–3.1			2002/11/09 21:50–23:21	G	1.1–7.2	0.55	$|\theta _{a}| \gtrsim 30^{\circ}$ 12:00–24:00 MLT
		0.2			1.8	2.4			3.1	4.9	2002/11/10 00:18–01:49	G	1.1–7.2	0.55	$\theta _{a}\approx 50^{\circ}-80^{\circ}$ 02:00–04:00 MLT
[49]	Villante et al. (2022)			1.7-2.2			2.7			3.7-4.2 4.9	1998/06/13 20:30–22:30	SW	1.0-5.0	0.28	
					2.0		2.5-2.7	3.0		4.0-4.2 4.5		SW	1.0-5.0	0.28	
					1.7–2.0		2.5	2.9-3.2	3.4	3.9-4.2 4.9		M	1.0-5.0	0.28	11:45–13:45 MLT
				1.5-1.7	2.0-2.2					3.7-3.9 4.2-4.4		M	1.0-5.0	0.28	15:48–17:48 MLT
				1.5–1.7	2.0–2.2		2.5	2.9-3.2	3.4-3.7	4.2–4.4 4.7-4.9		G	1.0-5.0	0.28	*θ* ≈ 21.6^∘^ − 73.4^∘^ 05:55–23:03 MLT
				1.5-1.7	2.0-2.2			2.9-3.4		4.4-4.7 3.9	2002/05/18 20:00–22:00	SW	1.0-5.0	0.28	
			1.2	1.7			2.7-2.9			3.7, 4.7		SW	1.0-5.0	0.28	
			1.2	1.7	2.2		2.5-2.7	2.9-3.2		4.4-4.7 3.7		M	1.0-5.0	0.28	11:15–13:15 MLT
				1.5-2.0			2.5-2.7		3.2-3.4	4.2-4.4 3.7		M	1.0-5.0	0.28	15:18–17:18 MLT
			1.2		1.7-2.2		2.5-2.7		3.4-3.7	3.9-4.2 4.4-4.7		G	1.0-5.0	0.28	*θ* ≈ 21.6^∘^ − 73.4^∘^ 05:25–22:33 MLT

^1^The results pertain: ionospheric measurements from radar (I), photometer (P), and Ultraviolet Imager (UVI); geomagnetic measurements from ground stations (G); magnetospheric measurements in the magnetosphere (M), magnetosheath (MSH), and plasmasheet (PSH); solar wind measurements (SW).

^2^Excluded from the comprehensive review data analysis.

^3^Statistical analysis.

In the following, the latitude of the observing stations is provided in the same coordinate system as in the original papers: geographic ($\vartheta $); geodetic ($\vartheta _{g}$); geomagnetic ($\theta $); invariant ($\theta _{I}$); the Eccentric Dipole Field Line latitude (EDFL; Grant and Rosner 1992); the Corrected Geomagnetic latitude (CGM, most frequently adopted) and the Polar Anglo-American Conjugate Experiment (PACE) Geomagnetic latitude (PGM), later referred to as Altitude-Adjusted Corrected (AACGM) latitude (Baker and Wing 1989; Shepherd 2014), which are identified with the same symbol, $\theta _{a}$, due to their close correspondence. Details of the data analysis and results at each station are summarized in Table 1 (for radar measurements, we provide the latitude and MLT where the waves are observed, when available; otherwise, we report the field of view, FOV, of the radar). More details are available in the supporting information Table S1.

Additionally, to allow a progressive approach to the topics in question, we organized our paper as follows:

i) We first discuss (Sect. 2) the “birth” of the CMS frequencies, as they originally appeared in the scientific literature, reporting the repeated analyses (and interpretations) that were proposed for the same set of (few) events identified in six selected days (1988 – 1989) in the measurements of the HF radar of Johns Hopkins University/Applied Physics Laboratories (JHU/APL) at Goose Bay and in the geomagnetic field measurements performed at observatories of the Canadian Auroral Network for the Origin of Plasmas in the Earth’s Neighborhood (OPEN) Unified Studies (CANOPUS).
ii) Subsequently, we discuss (Sect. 3) the implications of the shorter and longer term $f_{r}$ variations on the experimental observations, followed by a review of the results obtained at the same geomagnetic array in the following years (Sect. 4). Concluding this section, we report the observations of wave packets at discrete frequencies at other ground-based and radar arrays from low to high latitudes in both hemispheres over several decades (Sect. 5).
iii) We then approach (Sect. 6) the aspects of the relationship between magnetospheric fluctuations and SW structures impacting the magnetosphere (SW-driven fluctuations), considering separately magnetospheric events following the impact of interplanetary shocks and those possibly associated with SW compressive ($N_{SW}/P_{SW}$) variations (single events and statistical analysis). In this section, we also report the relationships, more occasionally proposed, between magnetospheric events and fluctuations of SW parameters other than $N_{SW}/P_{SW}$ (namely, IMF and $V_{SW}$ components).
iv) Lastly, we discuss some aspects (such as the role of the analytical methods and the position of the observing spacecraft; Sect. 7) which, in our opinion, should be carefully examined before proposing conclusions.

For comprehensive reviews on the ULF fluctuations, in general, the reader is referred to Saito (1969), Waters (2000), Villante (2007), Menk (2011), Zong (2022).

## The “Birth” of the CMS Frequencies

Earliest suggestions on the possible existence of magnetospheric fluctuations at discrete frequencies were proposed by short term analysis of the JHU/APL radar measurements at Goose Bay (Labrador, $\vartheta \approx $ 53.4°; FOV: $\theta \approx $ 65° – 85°; obviously, the latitude of the radar station does not correspond to the latitudinal range in which measurements are performed) and by the geomagnetic field measurements performed at observatories of CANOPUS ($\vartheta _{g} \approx $ 50.2° – 65.8°; $\theta \approx $ 60.7° – 73.3°; $\theta _{a} \approx $ 61.1° – 73.7°; $L \approx $ 4.2 – 12.3; Rostoker et al. (1995)). It is useful to remind, in this context, that the ionospheric radars detect oscillations of the convective plasma flow in the ionosphere, related to fluctuations of the drift velocities of irregularities in the F-region. These measurements have a much higher spatial resolution than ground-based magnetometers that integrate signals over a significant area of the ionosphere, with a consequent broadening of the spatial distribution of the resonant ULF structure (Hughes and Southwood 1976; Ziesolleck and McDiarmid 1994).

**Fig. 1:**
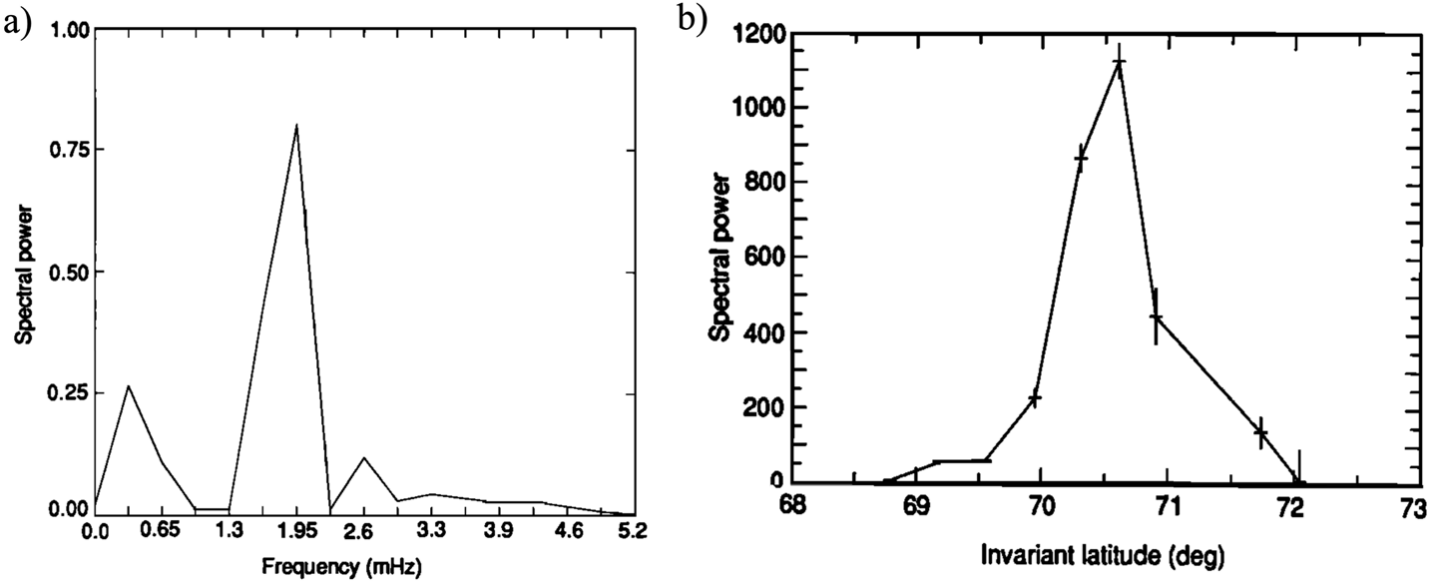
**The characteristics of the radar events observed at Goose Bay and CANOPUS.** (copyright by the American Geophysical Union, reproduced with permissions from Ruohoniemi et al. 1991, #1). a) The power spectrum of the Doppler velocity data from JHU/APL HF radar measurements at Goose Bay. b) The latitude profile of the spectral power at $f \approx $ 1.95 mHz

The first evidence for waves at a set of discrete frequencies, detected as quasi-monochromatic oscillations in the Doppler velocities, was proposed by (Ruohoniemi et al. 1991, #1 in Table 1) who examined 32-point time series of the radar measurements with sampling time $\Delta t= {96}\text{ s}$ spacing on 11 Jan., 1989, 04 – 07 UT during quiet geomagnetic conditions. Given the length of the examined intervals, $DT = {51.2}\text{ min}$, the frequency range under examination was $\Delta f \approx $ 0.3 – 5.2 mHz, with a frequency resolution $\delta f \approx $ 0.32 mHz. Starting at ≈ 04:00 UT (i.e. near MLT midnight, MLT being the Magnetic Local Time, with MLT midnight occurring at ≈ 03:40 UT), these oscillations persisted for several hours, with a spectrum dominated by a single-frequency component that exhibited a latitudinal peak in no more than 1° – 2° in extent (Fig. 1). Sharp spectral peaks were also identified at $f \approx $ 1.30 mHz ($\theta _{a} \approx $ 70.6°; $L \approx $ 9.1); at $f \approx $ 1.95 mHz ($\theta _{a} \approx $ 70.7°; $L \approx $ 9.2); at $f \approx $ 2.6 – 2.9 mHz ($\theta _{a} \approx $ 69.1°; $L \approx $ 7.9); at $f \approx $ 2.60 mHz ($\theta _{a} \approx $ 70.0°; $L \approx $ 8.6).

A new analysis of this event, carried out by Walker et al. (1992, #5) with better frequency resolution ($\Delta f \approx $ 0.1 – 5.2 mHz; $\delta f \approx $ 0.04 mHz; 04:15 – 07:55 UT), reported fluctuations at discrete frequencies, simultaneously occurring at different latitudes, at $f \approx $ 1.3, ≈ 1.9, ≈ 2.7 mHz and additional modes at $f \approx $ 0.8, ≈ 3.3 mHz. These fluctuations showed a packed structure, as expected if they were generated by a succession of pulses. Samson et al. (1991b, #2) extended the analysis of the night and early morning radar data (02:00 – 09:00 UT) to 6 selected days in more than two years: in addition to the event of Jan. 11, 1989, they considered also Sept. 28, Sept. 29, Oct. 2, 1988, Dec. 7; Dec. 8, 1989 (Kp ≈ 1 – 3; as for other cases, detailed analysis have been reported only for few time intervals; four, in the present case, see Table 1). They also examined the simultaneous geomagnetic field measurements at four sites of the CANOPUS network, aligned along a meridian ($\theta _{a} \approx $ 64.9° – 73.7°; $L \approx $ 5.1 – 12.1; $\Delta f \approx $ 0.1 – 5.2 mHz; $\delta f \approx $ 0.09/0.28 mHz). They identified, in several different occasions and in both data series (with the geomagnetic spectra considerably more variable than those of the radar measurements), matching and distinct spectral peaks at latitude dependent frequencies, such as $f \approx $ 1.3, ≈ 1.9, and ≈ 2.6 mHz. In particular, the frequencies of the peaks at $f \approx $ 1.3 and ≈ 1.9 mHz were observed to vary less than ±5% from the nominal value over the day, from day to day and month to month. Similar FLR events were not observed in the evening sector. Further analysis of the same data set by Samson et al. (1992a, #3) and Samson and Rankin (1994, #7) concluded that the four spectral peaks ($\delta f \approx $ 0.09 mHz; $\delta f \approx $ 0.14 mHz) occurred at $\theta _{a} \approx $ 71.5° ($f \approx $ 1.3 mHz), $\theta _{a} \approx $ 70.5° ($f \approx $ 1.8 – 1.9 mHz), $\theta _{a} \approx $ 69.75° ($f \approx $ 2.7 mHz), $\theta _{a} \approx $ 69.0° ($f \approx $ 3.3 – 3.4 mHz). As a matter of facts, these events showed the characteristics expected for FLRs, namely, the latitudinally narrow power peak, accompanied by a ≈ 180° phase variation. Moreover, the decreasing latitude with the increasing frequency of the observed peaks was considered consistent with FLRs excited by cavity modes in the magnetosphere; according to the time of their occurrence, it was suggested an energy source located in the magnetotail. Samson et al. (1992b, #4), examining data from CANOPUS ($\theta _{a} \approx $ 67.4° – 73.7°) and JHU/APL radar, noted that the frequencies of FLRs did not change during substorms. They also concluded that, at a given frequency, the FLR appears at the lowest latitudes near midnight and moves to higher latitudes in the local morning, consistent with the field lines more stretched near local midnight.

In general, the results of these analyses were considered consistent with FLRs driven by the four lowest order mode harmonics of the magnetospheric cavity, “ringing” between the magnetopause flank, at ≈ 14.5 R_E_, and turning points outside the plasmasphere, where Alfvén waves are reflected. Indeed, in the cavity mode scenario (Radoski 1974; Kivelson et al. 1984; Kivelson and Southwood 1985, 1986), a series of quantized global modes, oscillating at the natural eigenfrequencies of the magnetospheric cavity, can be set up, for example, by a SW impulse impinging the magnetopause. These compressional wave modes would then be expected to couple with FLRs (wave mode conversion to an Alfvén wave) at points where the cavity eigenfrequencies match the resonant frequency of the magnetospheric field lines. Clearly, the frequencies of these fluctuations are determined by properties internal to the magnetosphere, such as the Alfvén wave speed profile and the size of the magnetospheric cavity. The frequencies and the corresponding turning points $X_{it}$ predicted by Samson et al. (1991a) were $f_{1t} \approx $ 1.30, $f_{2t} \approx $ 1.88, $f_{3t} \approx $ 2.63 and $f_{4t} \approx $ 3.3 mHz, with $X_{1t} \approx $ 10.9, $X_{2t} \approx $ 7.9, $X_{3t} \approx $ 6.0, and $X_{4t} \approx $ 4.8 R_E_. As energy sources, Samson et al. (1991a) suggested the SW compressional pulses or the KHI in the low-latitude boundary layer. Extending the model, Samson et al. (1992a) proposed that the cavity behaved more like an open-ended waveguide in which, rather than azimuthally standing waves, the modes propagate downtail at the natural frequencies of the magnetosphere, in a cavity formed by the magnetopause and turning points where Alfvén waves are reflected on dipolar field lines. Samson and Rankin (1994) emphasized that the discrete, latitudinally localized, spectral peaks were exactly those expected if they were due to FLRs driven by cavity/waveguide modes and proposed a model for the coupling of the compressional energy in the waveguide modes to FLRs; they also suggested transient phenomena, such as impulsive variations of $P_{SW}$, as energy sources of these modes. According to previous arguments, although observed only in six selected time intervals, the frequencies $f_{1} \approx $ 1.3, $f_{2} \approx $ 1.9, $f_{3} \approx $ 2.6 – 2.7, and $f_{4} \approx $ 3.2 – 3.4 mHz were identified, in the scientific literature, as “cavity mode frequencies” (“CMS”, after the cavity mode model; Samson et al. 1991a, 1992a, these frequencies have been also reported as “magic” frequencies). More in general, Harrold and Samson (1992) proposed a model in which the oscillations at discrete frequencies in the range $f \approx $ 1 – 4 mHz could be also interpreted in terms of waves reflecting between the bow shock and the plasma sheet. All previous analyses concluded that the stability of the CMS frequencies would have implied a remarkable stability in the configuration of the magnetospheric cavity and the position of the magnetopause, considered inconsistent with a dynamic magnetosphere.

## The Role of the $f_{r}$ – Variations

According to previous arguments (and results), the identification of the (CMS) events at any given station might be strongly influenced by the latitudinal $f_{r}$-variation that makes the sites more or less suitable for observation of FLRs processes at given frequencies. On the other hand, additional aspects may be related to the occurrence and identification of events at discrete frequencies. In this sense, the well-known temporal variations of $f_{r}$ may deeply affect the results of similar analyses. Indeed, as shown by Waters et al. (1995), a conspicuous inverted U-shaped daily variation in $f_{r}$ occurs at CANOPUS stations. An analysis conducted during summer 1990 (solar maximum) revealed, at $\theta \approx $ 67° ($L \approx $ 6.6), a daily modulation, with $f_{r} \approx $ 2 mHz near local dawn, increasing up to ≈ 5 mHz by ≈ 06 – 07 LT, followed by a decrease to ≈ 2 mHz by ≈ 15 – 16 LT. It clearly implies a relevant daily excursion of $\lambda _{r}$ at a given frequency. On the other hand, similar results were also obtained at lower latitudes: for example, at the IMAGE network (19 magnetometer sites of the International Monitoring for Auroral Geomagnetic Effects; northern Scandinavia; $\vartheta \approx $ 60.5° – 78.9°; $\theta _{a} \approx $ 56.8° – 76.0°; $L \gtrsim $ 3.3; *MLT* ≈ *UT* + 3), Mathie et al. (1999a) revealed, during March 1994, 1995, 1996 (descending phase and solar minimum), a remarkable MLT dependence of $f_{r}$ that was above the range of the CMS frequencies through the entire dayside sector at stations located below $\theta \approx $ 65°. Similar features were also clearly detected at low latitudes for waves in the Pc3 range (Vellante et al. 1996).

In addition, $f_{r}$ experiences well documented variations also at longer time scales (27-day; annual, with summer values greater that the winter ones; Vellante et al. 2007; Archer et al. 2015). On the other hand, the solar cycle also controls the organization of the SW flow, with a more or less regular 27 - day recurrence of high velocity streams (HVS) and corotating interaction regions (CIR) at solar minimum and an irregular manifestation of coronal mass ejections (CME/ICME) at solar maximum. This implies remarkable differences in the SW characteristics and in the SW-magnetosphere interaction during different phases of the solar cycle in terms of energy transfer, magnetospheric configuration and size, characteristics of the magnetospheric plasma populations and geomagnetic activity (Katsavrias et al. 2012). In particular, the mass density in the magnetosphere might change by a factor ≈ 4 over a solar cycle, possibly determining a change of a factor ≈ 2 in the frequency of standing Alfvén waves, cavity/waveguide modes and magnetopause surface modes, with lower values at solar maximum (Vellante et al. 2007; Denton et al. 2011). In addition, the impact of ISs, the consequent magnetospheric compression, the variation of the field line length and the enrichment of the plasma mass density along the field line may determine relevant and rapid variations of $f_{r}$, especially at high latitudes.

In conclusion, the CMS frequencies proposed in the pioneering investigations ($f_{1} \approx $ 1.3, $f_{2} \approx $ 1.9, $f_{3} \approx $ 2.6 – 2.7 and $f_{4} \approx $ 3.2 – 3.4 mHz; Ruohoniemi et al. 1991; Samson et al. 1991a, 1992a; Walker et al. 1992) should be more properly referred to a very narrow latitudinal region ($\theta _{a} \approx $ 69° – 72°), MLT interval (midnight and early morning hours), season (autumn/winter months), phase of the solar cycle (maximum) and quiet/moderate geomagnetic activity.

## Other Observations at CANOPUS

Following the pioneering investigations, several papers examined the aspects of the occurrence of magnetospheric fluctuations at discrete frequencies, considering, at CANOPUS, different MLT intervals and latitudinal ranges, conducting longer term investigations and adopting different methods of analysis.

Rather than considering single events, Boulet (1992, #6) explored the possible occurrence of peaks at $f \approx $ 1.3, ≈ 1.9 and ≈ 2.6 mHz in the nightside, seasonally averaged spectra from seven stations; they concluded that these peaks were sometimes present and sometimes absent, with no explicit dependence on season, MLT and geomagnetic activity (Ziesolleck and McDiarmid 1995).

The existence and the stability of favorite sets of frequencies in the distribution of Pc5 events and their possible occurrence not only in post-midnight sector were addressed by Ziesolleck and McDiarmid (1994, #8) (Jan. 1993; eleven sites; $\theta \approx $ 60.7° – 73.2°, $L \approx $ 4.2 – 12.1) and extended by Ziesolleck and McDiarmid (1995, #9) (entire 1993; descending phase; five sites, $\theta \approx $ 64.4° – 73.2°; $L = 4.2 - 12.3$; $\Delta f \approx $ 1.0 – 6.0 mHz; $\delta f \approx $ 0.14 mHz). These analyses provided further support for the existence of discrete FLRs (mostly between $f \approx $ 1 – 3 mHz) that were observed through the dayside and dusk magnetosphere. In particular, Ziesolleck and McDiarmid (1994) identified numerous events at $f \approx $ 1.8 – 1.9, ≈ 2.5 – 2.6 and ≈ 3.4 mHz. Noticeably, they found also events at frequencies such as $f \approx $ 1.5, ≈ 2.2 mHz which do not fit in the cavity/waveguide model, as well as events at higher frequencies, $f \approx $ 4.0 – 6.0 mHz. Additionally, while some of the dominant frequencies were stable within ±0.5 mHz over intervals of several hours, other signals revealed a more considerable frequency variation not only within a particular LT sector, but also from day to day; moreover, the characteristics of afternoon and evening events were not consistent with FLRs. From the entire year analysis, Ziesolleck and McDiarmid (1995) concluded, in general, for a broad frequency distribution of events between $f \approx $ 1 – 5 mHz, with some indication of preferential frequencies near $f \approx $ 2.0 mHz, near $f \approx $ 3.0 mHz and near $f \approx $ 4.0 mHz (at lower latitudes); on the other hand, they did not find any indication for an increased occurrence at $f \approx $ 1.3 mHz. Remarkably, they concluded that the CMS frequencies did not appear particularly distinguished from other frequencies, except, perhaps, the $f \approx $ 1.9 mHz peak. Indeed, other frequency sets revealed similar occurrence rates: namely, $f \approx $ 1.1, ≈ 2.1, ≈ 3.0, ≈ 4.0 mHz; $f \approx $ 1.5, ≈ 2.3, ≈ 3.0 and ≈ 4.3 mHz; $f \approx $ 1.5, ≈ 2.3, ≈ 3.0, and ≈ 3.7 mHz. The MLT distribution of events revealed two occurrence peaks almost symmetric with respect to local noon, with post-noon occurrence lower than in the pre-noon sector; in addition, while the morning and noon events revealed FLR characteristics, toroidal mode characteristics emerged in the afternoon and evening events; no relevant seasonal variation in the event occurrence and characteristics was evidenced by this analysis.

**Fig. 2:**
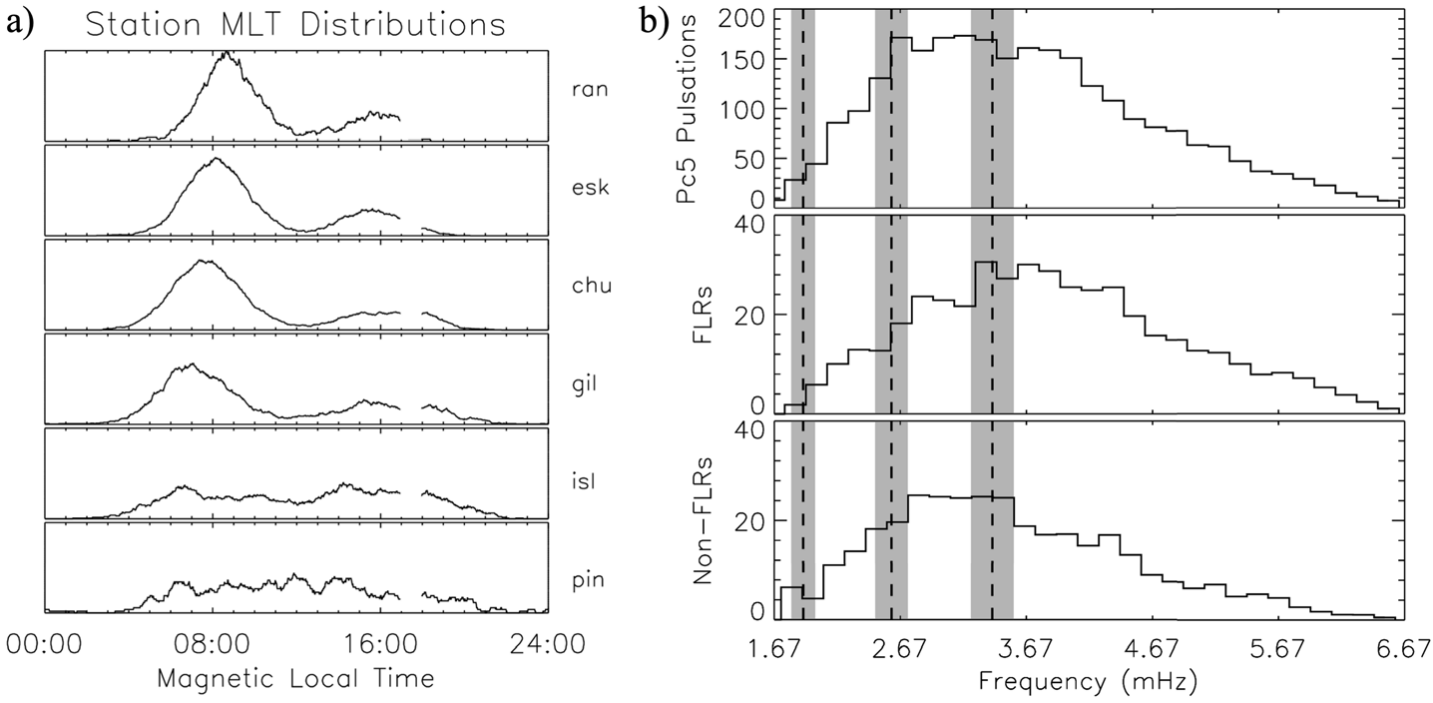
**The characteristics of the geomagnetic events observed at CANOPUS.** (copyright by the American Geophysical Union, reproduced with permissions from Baker 2003, #25). a) The occurrence distributions of the selected events versus MLT and $\theta _{a}$ ($\theta _{a} \approx $ 61.2°–73.7°) at six stations of the CANOPUS array; the panels are autoscaled. b) The frequency distributions of all events (top panel); for FLRs (middle panel), for non-FRLs; the shaded areas indicate the frequency bands (±5%) centered around $f\approx $ 1.9, $f\approx $ 2.6 and $f\approx $ 3.4 mHz

Approximately 10 years later, examining 1870 days (≈ 50%) from solar maximum 1989 to 1999, Baker (2003, #25) analyzed data from the “Churchill line” (7 stations north-south aligned; $\theta _{a} \approx $ 61.2° – 79.7°; $L > \approx $ 4.3) and 4 from the east-west line ($\theta _{a} \approx $ 65.9° – 67.9°; $L \approx $ 6.0 – 7.1). In this case, the frequency range (Pc5, $\Delta f \approx $ 1.7 – 6.7 mHz; $\delta f \approx $ 0.17 mHz) did not allow to investigate the possible occurrence of the peak at $f \approx $ 1.3 mHz. Out of 3334 visually selected events (with higher occurrence and power during periods of high $V_{SW}$), 636 (≈ 19%) were excluded from further analysis because they exhibited multifrequency components. The occurrence of the remaining 2698 events maximized at $\theta _{a} \approx $ 69.7° (“chu”, in Fig. 2a), practically vanishing above $\theta _{a} \approx $ 73.7° (“ran”), poleward of the open-closed field line boundary. In agreement with Ziesolleck and McDiarmid (1995), the MLT dependence showed a clear dawn/dusk asymmetry, more evident above $\theta _{a} \approx $ 67.4° (“gil”). Among these, 450 events (≈ 17%) showed FLR characteristics and mostly occurred with maximum amplitude at high auroral latitudes during the morning hours. Assigning the same frequency to events occurring within ±5% of the central value of each bin, the global analysis at all stations revealed a broad frequency distribution, with higher percentages between $f \approx $ 2.5 – 4.5 mHz and no evidence for stable and recurrent frequencies (Fig. 2b).

## Other Radar and Ground-Based Observations

The observation of wave packets at different discrete frequencies, approximately in the range $f \approx $ 1 – 5 mHz, occurring almost simultaneously at different sites has been investigated at several radar facilities and geomagnetic arrays over many years. Here we report the results more strictly related with the present review.

**Fig. 3:**
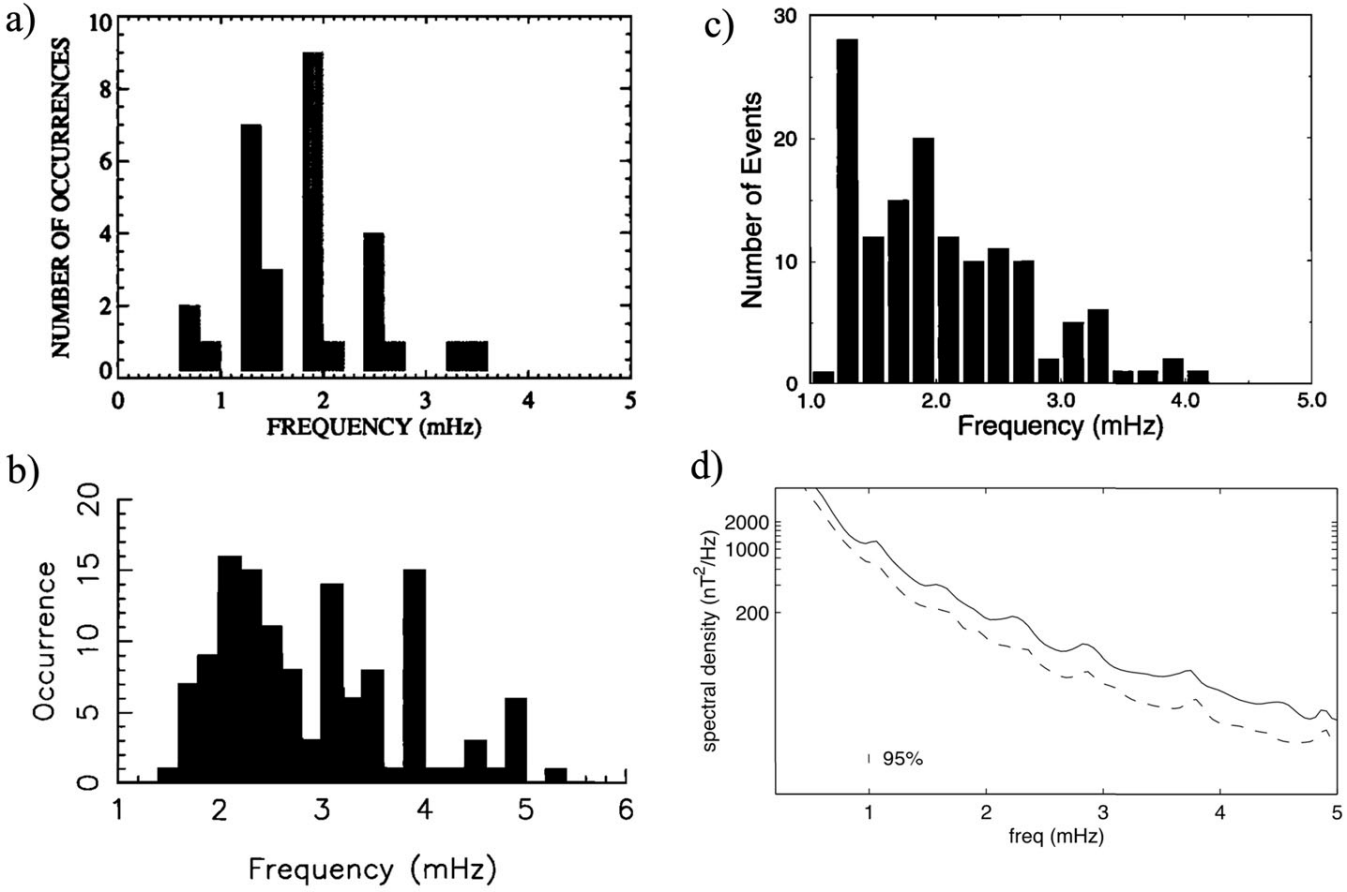
**The frequency distribution of events at SuperDARN, SAMNET, IMAGE and AQU.** a) (copyright by the American Geophysical Union, reproduced with permissions from Fenrich et al. 1995, #10) The frequency distribution of the 12 events observed from three HF radars of SuperDARN; the frequency bins are 0.2 mHz wide, starting from a bin centered at 0.1 mHz. b) (copyright by the American Geophysical Union, reproduced with permissions from Chisham and Orr 1997, #14). The frequency distribution of the 129 events observed at SAMNET. c) (copyright by the Chinese Geophysical Society, reproduced with permissions from Mathie et al. 1999a, #20). The frequency distribution of 137 events observed at IMAGE in March 1994, 1995, 1996. The data are organized in 0.2 mHz bins. d) (copyright by the authors, reproduced with permissions from Villante et al. 2001, #21). The average power spectra of the H (solid track) and D component (dashed) at AQU in the post-noon sector and during high $P_{SW}$ intervals (1997 – 1998). The error bars correspond to the 95% confidence interval
- **SABRE**
  Provan and Yeoman (1997, #12) visually selected events occurring in the post-midnight sector (01:45 – 02:45 UT; ≈ 02:00 – 03:10 MLT) during 16 active days between 1981 (descending phase) and 1989 (maximum) at the SABRE radar at Wick (Scotland, $\vartheta \approx $ 58.4°; $\Delta f \approx $ 1.0 – 7.0 mHz; $\delta f \approx {0.28}\text{ mHz}$). Fluctuation events were identified at $\theta _{a} \approx $ 61° – 67°; $L \approx $ 4.3 – 6.6). The average spectra revealed harmonic series with a distinct peak at $f \approx $ 1.8 mHz, a broad region of enhanced power at $f \approx $ 2.4 – 2.8 mHz and another peak at $f \approx $ 3.2 mHz, with the frequencies of the spectral peaks decreasing with increasing latitude. These results were interpreted in terms of FLRs at frequencies consistent with the CMS frequencies. The absence of events at $f \approx $ 1.3 mHz was imputed to the latitude of SABRE (i.e., field lines too short to support FLRs at these frequencies). On the other hand, an additional peak at $f \approx $ 1.6 mHz, observed at $\theta _{a} \approx $ 61°, did not fit with the harmonic series. As in other cases, the observed frequencies remained apparently constant for a number of days.
- **SAMNET**
  Chisham and Orr (1997, #14) examined, in more than a year (≈ 1988, solar maximum), the characteristics of 129 events persisting for more than three cycles with a stable period and visible at all stations (seven) of the SubAuroral Magnetometer Network in United Kingdom (SAMNET; $\vartheta \approx $ 53.9° – 65.1°; $\theta _{a} \approx $ 51.0° – 61.3°; $L \approx $ 2.6 – 4.4; $\Delta f \approx $ 1.6 – 6.0 mHz; $\delta f \approx $ 0.2 mHz, H and D components; ≈ 3:00 – 21:00 MLT). The analysis confirmed a marked MLT asymmetry in the characteristics of fluctuations. Indeed, while the morning events (much more frequent) were interpreted as the signatures of higher latitude FLRs, the afternoon events were considered the signatures of compressional waves, mainly cavity/waveguide modes, with little evidence of coupling to FLRs. Note, however, that while in the morning the plasmapause often lied across the SAMNET array ($L_{PP} \approx $ 3), in the afternoon the entire array was within the plasmasphere ($L_{PP} \approx $ 5). Finally, no events were detected between 21:00 – 03:00 MLT. The global frequency distribution of the selected events (Fig. 3b) did not support any evidence for CMS frequencies showing peaks at $f \approx $ 2.0 – 2.4, ≈ 3.1, ≈ 3.9 mHz and, possibly, ≈ 4.9 mHz.
- **IMAGE**
  From the analysis conducted by Mathie et al. (1999a) at the IMAGE network during March 1994, 1995, 1996, Mathie et al. (1999b, #20) visually selected 137 events in the dayside MLT sector (≈ 06 – 18 MLT; $\Delta f \approx $ 1.0 – 5.0 mHz; $\delta f \approx $ 0.18 mHz; H component). As in other analysis, the fluctuations activity revealed a strong MLT dependence, with FLR characteristics dominant in the local morning and late afternoon sectors, but mostly absent around local noon and in the early afternoon. Although with a poor day-to-day stability, the analysis revealed that the CMS frequencies were prominent in these observations: indeed, dominant frequencies appeared at $f \approx $ 1.2 – 1.4 (most prominent), ≈ 1.8 – 2.0, ≈ 2.4 – 2.6 (less evident) and ≈ 3.2 – 3.4 mHz, together with some evidence for an additional enhancement at $f \approx $ 4.0 – 4.2 mHz (Fig. 3c). It was suggested that the CMS frequencies might represent the most frequently occurring eigenfrequencies of the magnetospheric cavity, subject to variability in response to the changing nature of the waveguide.
- **Low latitudes**
  Francia and Villante (1997, #15) examined the possible occurrence of modes at discrete frequencies in the range of interest at low latitudes (where $f_{r}$ occurs in the Pc3 band) by conducting a statistical analysis of 3-hour power spectra of the H and D geomagnetic field components at L’Aquila (AQU, Italy, $\theta \approx $ 36.2°, $L \approx $ 1.6; *MLT* ≈ *UT* + 1:40; $\Delta f \approx $ 0.3 – 5.0 mHz; $\delta f \approx $ 0.09 mHz) during solar minimum (1985 – 1986) and solar maximum (1989 – 1990). At solar minimum, the daytime average spectra showed some evidence for small and broad enhancements on the H component, approximately around $f \approx $ 1.2 – 1.4, ≈ 1.8 – 2.0, ≈ 2.4 – 2.6 mHz (and, with less evidence, at ≈ 3.4 – 3.6 and around ≈ 4.2 mHz). These aspects became more evident during a two months interval characterized by an average $V_{SW} \approx {510}\text{ km}\text{ s}^{-1}$. At solar maximum (1989 – 1990), similar broad and small enhancements occurred around $f \approx $ 1.9 mHz and, less evident, at other frequencies. At the same station and with a similar data processing, during the ascending phase 1997 – 1998, Villante et al. (2001, #21) reported some evidence for similar enhancements around $f \approx $ 1.1, ≈ 1.7, ≈ 2.3, ≈ 2.8 and ≈ 3.7 mHz, more explicit in the afternoon and during periods of high $P_{SW}$ (Fig. 3d). An interesting result was also proposed by Villante et al. (2001): they examined the same 3-month interval analyzed by Mathie et al. (1999b) and revealed the occurrence at AQU of power enhancements approximately at the same frequencies as the ones at the IMAGE array, suggesting that modes at the same frequencies might be observed from auroral down to low latitudes.
- **Antarctica**
  A statistical analysis of the power spectra of H and D was conducted by Villante et al. (1997, #13) at polar cap latitudes in Antarctica (Terranova Bay, TNB, *Mario Zucchelli Station*; $\theta \approx $ 77.3°S, $\theta _{a} \approx $ 80.0°S; *LT* ≈ *UT* + 13; *MLT* ≈ *UT* − 8; $\Delta f \approx $ 0.7 − 5.0 mHz; $\delta f \approx $ 0.09 mHz). They analyzed data from three austral summers during the maximum of solar activity (1988 – 1991). Note that around MLT noon, the station approaches the dayside cusp. Surprisingly, at those latitudes (i.e., poleward of the open-closed field line boundary), the analysis revealed small-amplitude broad-enhancements in the average spectra of the H component, more evident during daytime MLT intervals and higher SW speeds. Figure 4a shows the results obtained selecting the dominant peaks in each spectrum: as can be seen, peaks of events occur approximately at $f \approx $ 1.0 – 1.3, ≈ 1.8 – 2.0, ≈ 2.7 – 2.9, ≈ 3.2 – 3.5, ≈ 3.9 – 4.1 and ≈ 4.5 – 4.7 mHz.

- **SuperDARN**
  Fenrich et al. (1995, #10) visually selected 12 FLR events from three HF radars of the Super Dual Auroral Radar Network (SuperDARN; Saskatoon, $\vartheta \approx $ 52.16°, Kapuskasing, $\vartheta \approx $ 49.39°, and Goose Bay; latitudinal range of observed events $\theta _{a} \approx $ 68.2° – 74.0°; $L \approx $ 7.3 – 13.2; $\Delta f \approx $ 0.3 – 5.0 mHz; $\delta f \approx $ 0.16/0.31 mHz). These events occurred in the autumn/winter months on both the dayside and nightside, with quiet magnetospheric conditions, during 1988 – 1989 (solar maximum, a period including earliest observations; in particular, on Jan. 1, 1989, they confirmed the mode at $f \approx $ 0.8 mHz proposed by Walker et al. (1992)) and during 1993 – 1994 (descending phase, including the period examined by Ziesolleck and McDiarmid (1995)). The most striking result was the recurrence of FLRs at the same frequencies at various locations, with different frequencies dominating at different times. For resonances simultaneously occurring at different frequencies, the lower frequency ones were localized at higher latitudes. Organized in 0.2 mHz bins, the frequencies clustered around the CMS ones, centered at $f \approx $ 1.3 (extending up to ≈ 1.6), ≈ 1.9 and ≈ 2.5 – 2.6 mHz (Fig. 3a). Some events occurred also at lower ($f \approx $ 0.8 – 1.0 mHz) and higher frequencies ($f \approx $ 3.3 – 3.6 mHz). More than twenty years later, Shi et al. (2018, #45) examined the ionospheric signatures of fluctuations in a wide frequency band from 17 SuperDARN stations in the northern hemisphere ($\Delta f \approx $ 1.7 – 40.0 mHz; $\delta f \approx $ 0.07 mHz; 2010 – 2016, from the solar minimum to the new descending phase). The Pc5 events were extensively observed at high and polar latitudes, with a clear trend toward lower latitudes as the frequency increases. In general, their occurrence rate maximized in winter, in the MLT dusk sector (with a peak approximately at $\theta _{a} \approx $ 70°) and during periods characterized by a northward orientation of the interplanetary magnetic field (IMF) and low geomagnetic activity. In the polar region, the occurrence rate maximized at $\theta _{a} \approx $ 80° – 83°, in the pre-midnight sector. Regarding the frequency distribution, the events mostly occurred below $f \approx $ 4.0 mHz, with a most probable frequency of $f \approx $ 2.08 mHz and no evidence of a set of favorite frequencies. An analysis of the most relevant events revealed a peak of occurrence at $f \approx $ 2.36 mHz.

**Fig. 4:**
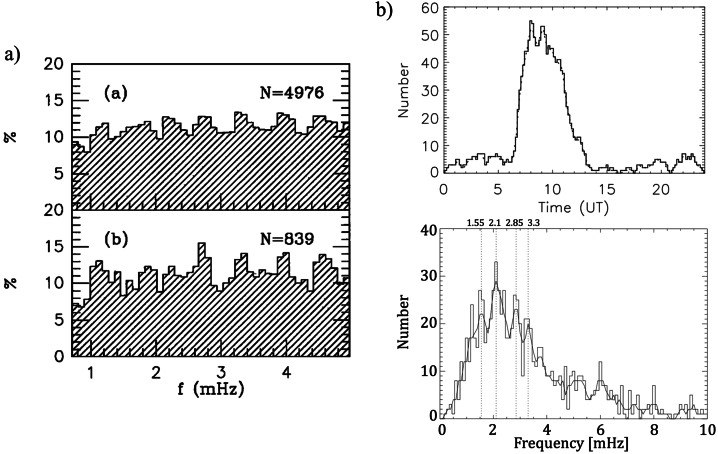
**The characteristics of events observed in the southern hemisphere.** a) (copyright by the American Geophysical Union, reproduced with permissions from Villante et al. 1997, #13). The frequency distribution of the spectral peaks at TNB (Antarctica) for all analyzed intervals during 1988 – 1991 (top) and during periods of $V_{SW} >$ 430 km/sec (bottom). b) (copyright by the American Geophysical Union, reproduced with permissions from Norouzi-Sedeh et al. 2015, #39). Top panel, the number of events observed by radars versus UT (*LT* = *UT* + 9.4). Bottom panel, the relative frequency distribution ($\delta f =$ 0.1 mHz; a three-points smoothed curve is also shown): favored frequencies appear at $f\approx $ 1.3, ≈ 1.6, ≈ 2.1, ≈ 2.9, ≈ 3.3 mHz and higher
- **CANOPUS, GREENLAND, WDC, IMAGE, SAMNET and 210°MM**
  Lee et al. (2007, #29) investigated the global characteristics of the ULF waves detected during the main phase of the great magnetic storm of 24 March 1991 (descending phase) examining data from several ground-based magnetometer arrays (CANOPUS, GREENLAND, WDC, IMAGE, SAMNET, and 210° Magnetic Meridian (MM) Network; $\theta _{a} \approx $ 46.2°S – 85.8°; $\Delta f \approx $ 1.0 – 6.0 mHz; $\delta f \approx $ 0.28 mHz). They identified several dominant spectral peaks in different time intervals, more explicit at $f \approx $ 1.7 mHz (observed at unusually low latitudes, with maximum amplitude at $L \approx $ 4.3; 12:00 – 13:40 UT; $\theta _{a} \approx $ 61°; 12:49 – 16:49 MLT), $f \approx $ 1.9 mHz ($L \approx $ 4; 10:10 – 11:10 UT; $\theta _{a} \approx $ 60°; 07:21 – 14:19 MLT), $f \approx $ 2.8 mHz ($L \approx $ 3.6; 08:15 – 09:15 UT; $\theta _{a} \approx $ 58° – 74°; 05:26 – 12:24 MLT). The characteristics of these fluctuations were considered consistent with FLRs and supported the hypothesis of the existence of cavity or waveguide modes in the magnetosphere. As a matter of facts, for the observed modes, $\lambda _{r}$ was much lower than normal, consistent with a relevant variation of the local eigenfrequencies compared to non-storm times.
- **TIGER** **and Macquarie Island**
  Norouzi-Sedeh et al. (2015, #39) analyzed data from the two Tasman International Geospace Environment Radars (TIGER/SuperDARN, Bruny Island; $\vartheta \approx $ 43.4°S and Invercargill, New Zealand; $\vartheta \approx $ 46.5°S; combined FOV: $\theta _{a} \approx $ 54.2°S – 87.6°S; $\Delta f \approx $ 0.5 – 30.0 mHz; $\delta f \approx $ 0.17 mHz) and from the magnetometers at Macquarie Island ($\vartheta \approx $ 54.5°S; $\theta _{a} \approx $ 64.3°S, $L \approx $ 5.3; $\Delta f \approx $ 0.5 – 30.0 mHz; $\delta f \approx $ 0.09 mHz; H component). They analyzed more than 300 days during 2007 – 2009 (around the solar minimum), identifying 194 radar/magnetometers coincident events over 89 different days. As shown in Fig. 4b, most of the events, lasting for ≈ 1 h, were observed in the afternoon/evening sector (≈ 6 – 12 UT; ≈ 15 – 21 LT; ≈ 13 – 23 MLT given the maximum possible excursion of ≈ 2 h between LT and MLT in the radar FOV) with the most frequent occurrence near ≈ 17 LT (≈ 15 – 19 MLT) during quiet geomagnetic conditions. Favored frequencies, detected in radar measurements, were in the band $f \approx $ 1 – 3 mHz (Fig. 4c) with favored frequencies $f \approx $ 1.6, ≈ 2.1, ≈ 2.9, and ≈ 3.3 mHz and no obvious variation of frequency with latitude. The decrease in the event occurrence below $f \approx $ 1 mHz was due to the pre-filtering of the data between 0.5 – 30.0 mHz. A “bump” between $f \approx $ 4 – 6 mHz was imputed to the variable location of the plasmapause, determining the variable $f_{r}$ at those latitudes. The analysis of the power spectra of the geomagnetic field, during days in which wave events were detected in the radar measurements, revealed, in the LT-dependent spectra, regularly spaced favored frequencies at $f \approx $ 0.8, ≈ 1.2, ≈ 1.7, ≈ 2.1, ≈ 2.6, and possibly, ≈ 3.1 mHz, most evident between 18:30 – 21:30 LT (≈ 16:30 – 23:30 MLT).

## SW Driven Magnetospheric Events

Several papers analyzed the occurrence and the characteristics of the magnetospheric fluctuations, focusing attention on their association with sharp variations/fluctuations of the SW parameters. Some of them were published before the appearance of the CMS frequencies in the scientific literature (e. g., Voelker 1966; Saito and Matsushita 1967; Kaufmann and Walker 1974; Fukunishi 1979; Baumjohann et al. 1984; Sibeck et al. 1989; Potemra et al. 1989; Erlandson et al. 1991). Those more strictly related to aspects examined in the present review are discussed hereafter.

### Fluctuations Following the Impacts of IS/$P_{SW}$ Fronts

Following Storm Sudden Commencements (SSCs) or Sudden Impulses (SIs), typically caused by the impact on the magnetopause of IS or other $P_{SW}$ fronts, fluctuations at discrete frequencies have been often detected in the magnetosphere. It is worth noting in this sense that, especially in the oldest analysis related to the impact of SW fronts, the possible occurrence of SW fluctuations at frequencies comparable to those of the magnetospheric fluctuations was not explicitly analyzed.

**Fig. 5:**
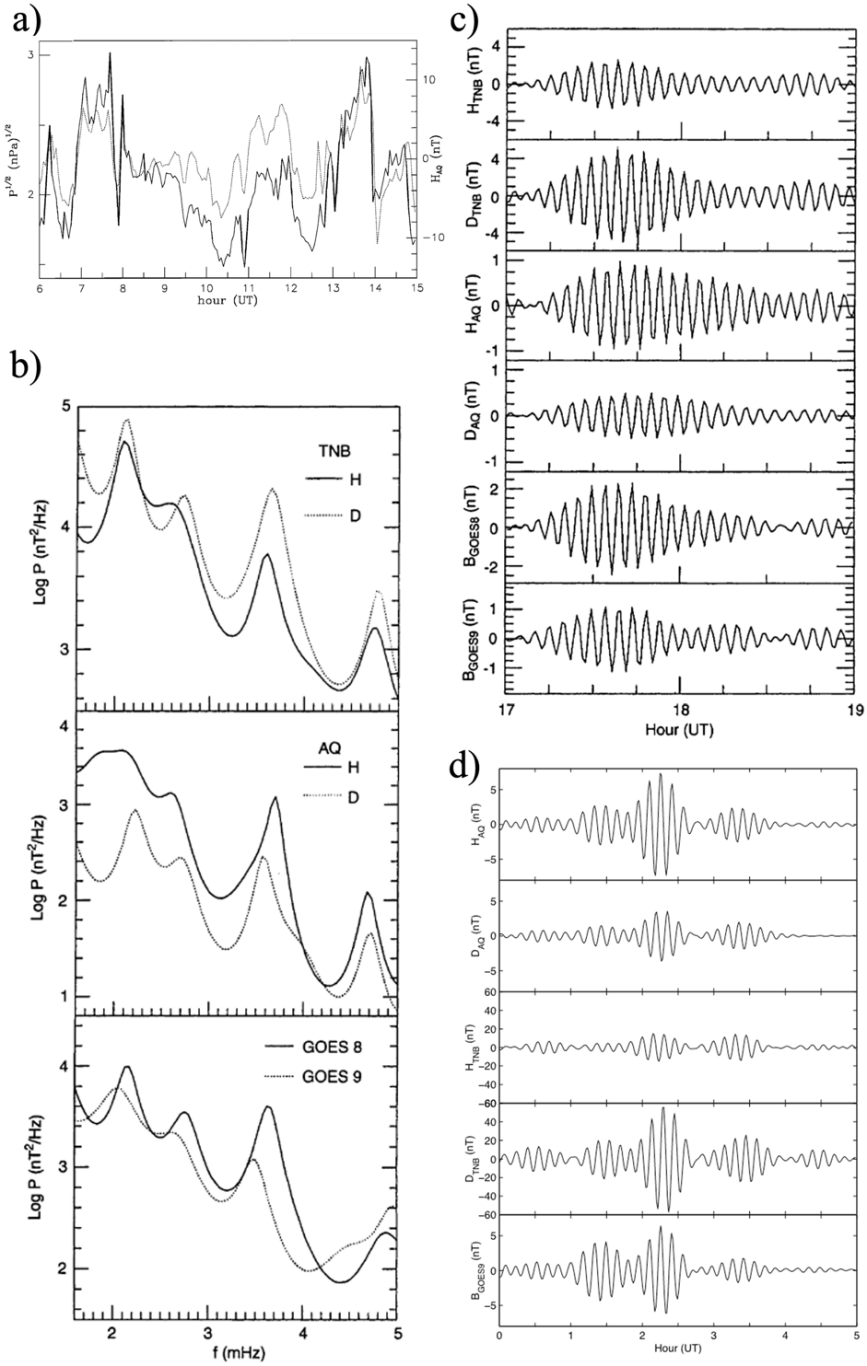
**Modes simultaneously observed at the geostationary orbit, low latitudes, and Antarctica.** a) (copyright by the American Geophysical Union, reproduced with permissions from Francia et al. 1999). The comparison between the H component in the dayside at AQU (dotted trace) and the square root of the SW dynamic pressure (Wind, solid trace; April 11, 1997) reveals a close correspondence between these parameters. The H data have been shifted for the time delay of 57 min. b) (copyright by the European Geosciences Union, reproduced with permissions from Lepidi et al. 1999, #18). The power spectra of the geomagnetic field components at TNB (April 11, 1997, 17 – 19 UT; top panel) and AQU (central panel) and those of the magnetic field magnitude at GOES8 and GOES9 (bottom panel) show a remarkable similarity and a common peak at $f\approx $ 3.6 mHz. c) (after Lepidi et al. 1999, #18). The filtered data at $f\approx $ 3.6 mHz at TNB, AQU and GOES8 show very similar waveforms. d) (copyright by the authors, reproduced with permissions from Villante et al. 2001, #21). The filtered data at $f =$ 1.8 mHz of the geomagnetic field components at AQU and TNB and those of the field magnitude at the geostationary orbit; January 11, 1997, 00 – 05 UT
- **ACE/GOES/ground - based networks**
  The correspondence between $P_{SW}$ variations and magnetospheric/geomagnetic fluctuations, highlighted by Francia et al. (1999), was clearly evidenced by Motoba et al. (2003) (see also Motoba et al. 2002) who showed that, on February 1998 (solar minimum), during a period of strongly southward IMF orientation, a series quasi-periodic $P_{SW}$ pulses at ACE likely determined periodic magnetospheric compressions at $f \approx $ 1.2 mHz, with greater amplitude around noon at GOES8, GOES9, and GOES10. Correspondingly, coherent oscillations of the geomagnetic field at the same frequency were observed on a global scale, from polar to equatorial latitudes (CANOPUS; IMAGE; Magnetospheric Array for Cap and Cleft Studies, MACCS; Circum-pan Pacific Magnetometer Network, CPMN; INTERMAGNET). Their signatures were considered consistent with those predicted for the modulation of the global ionospheric current systems.
- **Wind/GOES/CRRES/CANOPUS**
  A comparison between CANOPUS (eastern meridional sites and east-west chain; $\theta _{a} \approx $ 60.9° – 79.4°; $L \gtrsim $ 4.2; $\Delta f \approx $ 1.0 – 5.0 mHz; $\delta f \approx $ 0.06 mHz), auroral photometers ($\Delta f \approx $ 0.5 – 8.0 mHz; $\delta f \approx $ 0.11 mHz), GOES7 ($\Delta f \approx $ 0.1 – 8.0 mHz; $\delta f \approx $ 0.08 mHz) and CRRES magnetic and electric observations ($\Delta f \approx $ 0.1 – 8.0 mHz; $\delta f \approx $ 0.06 mHz) following a substorm on December 13, 1990 (after solar maximum; Lessard et al. 1999, #19) revealed, in all data set, a common spectral peak at $f \approx $ 2.1 mHz, approximately at the same latitude ($\theta _{a} \approx $ 63° – 69°), suggesting the occurrence of the same resonance mode on the ground and in the magnetosphere. Additional common peaks were also observed at $f \approx $ 1.4 and ≈ 1.7 mHz at a slightly higher latitude. These results were tentatively interpreted as waves in the magnetospheric waveguide (and, possibly, FLRs) triggered by a $N_{SW}$ enhancement (Wind).
- **ACE/Geotail/GOES/Polar/210°MM**
  According to Liou et al. (2008, #30), the arrival of a long-duration (≈ 100 min, ACE) $P_{SW}$ enhancement on September 26, 1999 (ascending phase) produced striking auroral oscillations that were observed by the Ultraviolet Imager (UVI; $\theta \approx $ 60° – 90°, 02:00 – 11:00 MLT) onboard the Polar spacecraft at discrete frequencies centered around $f \approx $ 1.8, ≈ 3.5, ≈ 4.8, ≈ 6.3, and ≈ 7.7 mHz ($\Delta f \approx $ 0.16 – 13.6 mHz; $\delta f \approx $ 0.16 mHz). The comparison with magnetometer measurements (H-component from several stations of the 210°MM network; $\theta \approx $ 29.4° – 70.3°) located in the dawn sector (≈ 03:56 – 06:16 MLT), where auroral fluctuations exhibited greatest amplitude, revealed that large amplitude geomagnetic fluctuations were observed simultaneously at two sites underneath the oval and at other stations from high to low latitudes. Similarly, large-amplitude waves of higher frequency ($f \approx $ 3 – 4 mHz) were also detected. The observed features were considered consistent with the global compression of the magnetopheric cavity and the fluctuations at discrete frequencies were proposed as indicative of cavity mode harmonics.
- **SuperDARN** **at Goose Bay**
  Mthembu et al. (2009, #33) examined the characteristics of the ULF fluctuations detected on November 11, 2002 (solar maximum), 06 – 08 UT, by the SuperDARN radar at Goose Bay ($\Delta f \approx $ 0.2 – 4.0 mHz; $\delta f \approx $ 0.14 mHz). They found relevant wave activity between ≈ 02:20 – 04:20 MLT, likely related to a sudden $P_{SW}$ change, with peaks at $f \approx $ 0.6, ≈ 1.3, ≈ 1.5, ≈ 1.9 mHz. The behavior of the prominent mode at $f \approx $ 1.9 mHz clearly showed FLR characteristics: the amplitude increased slowly with latitude from $\theta _{a} \approx $ 68.5°, peaked at $\theta _{a} \approx $ 71.3° ($\lambda _{r}$), and then decreased again; consistently, across $\lambda _{r}$ the phase changed by ≈ 200°.

- **IMP-8/GOES/VIKING/Huancayo**
  Comparing measurements from interplanetary (IMP-8) and magnetospheric spacecraft (GOES5, GOES8, VIKING) with ground magnetograms at a near equatorial station on August 3, 1986, ≈ 17:10 – 19:30 UT (Huancayo, Perù; $\vartheta \approx $ 12.0°S; *LT* ≈ *MLT* ≈ 12:10 – 16:30; solar minimum), Erlandson et al. (1991) showed that some dramatic $P_{SW}$ variations produced compressions and rarefactions of the magnetosphere which were interpreted in terms of fast mode waves, generated near the magnetopause and propagating anti-sunward into the inner magnetosphere. Visual inspection of their results reveals quasi-periodic $P_{SW}$ variations, mostly due to $N_{SW}$ variations, very roughly at ≈ 5 and ≈ 30 min (≈ 0.5 and ≈ 3.0 mHz).
- **ISEE/GOES/SMA** **and** **NAMN**
  Shimazu et al. (1995, #11) compared the occurrence of Pc5 fluctuations (H component) ($\Delta f \approx $ 0.6 – 10.0 mHz; $\Delta f \approx $ 0.56 mHz) at the Scandinavian Magnetometer Array (SMA, $\theta \approx $ 60.4° – 67.4°) and the North American Magnetometer Network (NAMN, $\theta \approx $ 62.9° – 79.7°; April – December 1978, ascending phase) and concluded that, in general, the fluctuation activity was longitudinally localized. They reported, however, a clear event simultaneously observed at similar latitudes: at College ($\theta \approx $ 64.9°), Fort Simpson ($\theta \approx $ 67.7°), and Fort Smith ($\theta \approx $ 68.1°), in the early morning (03:27 – 07:00 MLT), and at Kiruna ($\theta \approx $ 65.2°), in the evening (18:04 – 18:34 MLT). It occurred during northward IMF orientation and high SW speed ($V_{SW} \approx {620}\text{ km}\text{ s}^{-1}$; ISEE 3). In this occasion, very similar, composite waveforms were observed at the two stations, with components almost at the same frequencies with values mostly above the CMS range ($f \approx $ 3.3, ≈ 4.7, ≈ 5.9 and ≈ 7.1 mHz). Similar waveforms were simultaneously detected at low latitudes but were absent at the geostationary orbit (GEOS2 and GEOS3). It was suggested that this event was determined by the passage of a sharp $P_{SW}$ increase triggering cavity mode fluctuations in the magnetosphere.
- **Wind/GOES/low latitudes/Antarctica**
  Although not directly related to shock impacts/wave events, a study by Francia et al. (1999) about the geomagnetic field response to persistent and more or less regular $P_{SW}$ variations provided interesting results. The main event was on April 11, 1997, 06 −15 UT, during solar minimum but similar conclusions were obtained also for different days. The comparison between the behavior of the time delayed $\sqrt{P_{SW}}$ data and ground magnetic field (H component) measurements in the dayside at low latitude (AQU) revealed a spectacular and persistent correspondence between these parameters for time scales longer than 3 min ($f \lesssim $ 5 mHz; Fig. 5a), with a correlation coefficient R ≈ 0.74 (R ≥ 0.9 over intervals of ≈ 1 – 5 h) and peak amplitude of the geomagnetic response ($\Delta H/\Delta (\sqrt{P_{SW}})$ around noon. It clearly highlighted that the quasi-static variations of $P_{SW}$ are rapidly transmitted to the magnetosphere, possibly slightly altering the magnetospheric size and/or modulating the magnetopause current. Later in the same day, during a period characterized by fluctuation activity (17 – 19 UT; Lepidi et al. 1999, #18), a surprising correspondence emerged between the power spectra of the magnetic field magnitude (i.e., compressional fluctuations; $\Delta f \approx $ 1.6 – 5.0 mHz; $\delta f \approx $ 0.13 mHz) at the geostationary orbit and those at AQU/TNB with the simultaneous occurrence of modes at the same frequencies at all observing stations ($f \approx $ 2.1 – 2.2, ≈ 3.6 – 3.7, ≈ 4.7 – 4.8 mHz; Fig. 5b). Figure 5c shows a clear example of such correspondence for the mode at $f \approx $ 3.6 mHz observed by GOES9 on the morning side of the magnetosphere (≈ 08 – 10 LT), by GOES8 in the noon sector (≈ 12 – 14 LT), at AQU in the evening (≈ 18 – 20 LT; ≈ 19:40 – 21:40 MLT) and at TNB (with remarkable amplitude, especially along the D component), approaching the cusp (≈ 09 – 11 MLT). A similar correspondence between low latitude and Antarctic observations was evidenced on Jan. 10 – 11, 1997 (solar minimum) when a wide magnetic cloud reached the Earth causing intense geomagnetic activity. A comparison between AQU and TNB observations (Villante et al. 1998, #17) revealed that the major $P_{SW}$ enhancements were followed by SIs and intense geomagnetic activity at both stations while the power spectra (H and D component; $\Delta f \approx $ 0.8 – 5.0 mHz; $\delta f \approx $ 0.07 mHz) revealed relevant common peaks at $f \approx $ 1.0, ≈ 1.4, ≈ 1.8 and ≈ 2.2 (TNB) – 2.4 mHz (AQU). Moreover, as for the previous case, Villante et al. (2001, #21) reported a mode at $f \approx $ 1.8 mHz simultaneously observed at GOES9 in the late afternoon region (≈ 16 – 18 LT; Fig. 5d), at AQU in the night sector (≈ 02 – 04 LT; ≈ 03:40 – 05:40 MLT), and even deep in the cap at TNB (≈ 17 – 19 MLT; mostly along the D component). These observations clearly revealed that the same fluctuation modes were simultaneously observed in the entire magnetosphere and in both hemispheres.

### The Role of the $P_{SW}$/$N_{SW}$ Fluctuations

The possible correspondence between packets of SW compressive fluctuations and magnetospheric fluctuations has been investigated in several analysis.

#### Single Events

**Fig. 6:**
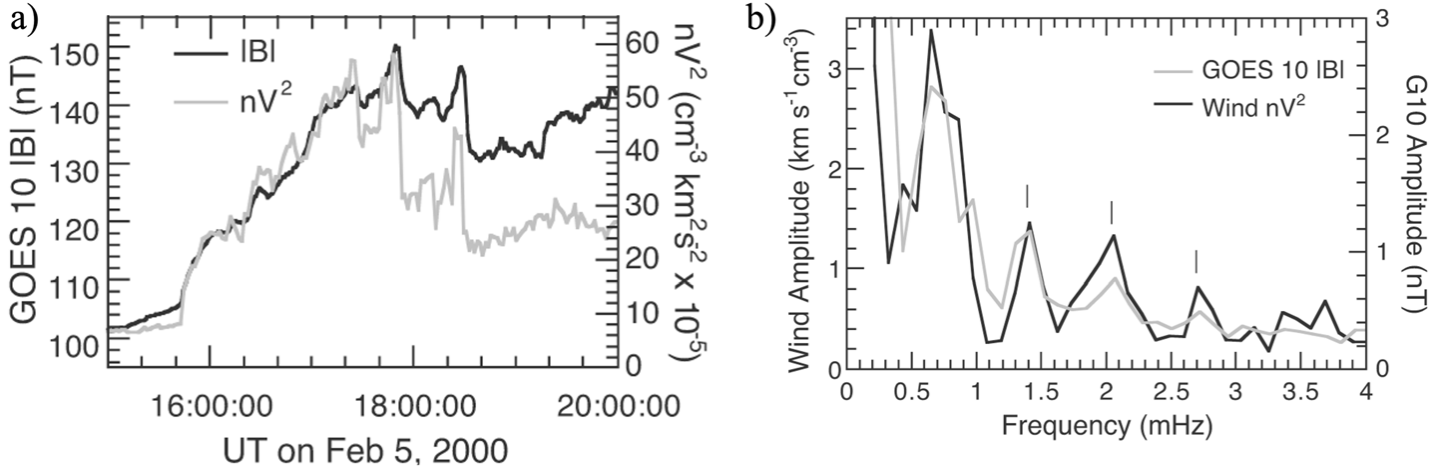
**A comparison between the** **SW** **pressure and the magnetospheric field variations.** (copyright by the American Geophysical Union, reproduced with permissions from Kepko et al. 2002, #23). Left panel, the comparison between the SW pressure and the magnetospheric field strength at the geostationary orbit (February 5, 2000; 15:45 – 18:30 UT). Right panel, the comparison between the relative power spectra
- **ACE/SHARE** **Radar**
  Eriksson et al. (2006a, #26) compared 10 events from the SHARE radar (Sanae, Antarctica, $\vartheta \approx $ 71.7°S; $\Delta f \approx $ 0.8 – 4.4 mHz; $\delta f \approx $ 0.08 mHz; waves power peaked at: $\theta _{a} \approx $ 65.6°S – 70.9°S; $L \approx $ 5.9 – 9.3; midnight/morning LT sector) and ACE observations (2000 – 2001; solar maximum). They confirmed that the ionospheric wave packet structure was in good agreement with the wave packet structure of $P_{SW}$. Globally, the highest correlation between ACE and SHARE observations was found in the band $f = 0.8$ – 1.4 mHz and, to a less extent, in the band $f = 2.0$ – 2.6 mHz.
- **Wind/GOES/AQU**
  Villante et al. (2007, #28) examined nine remarkable events of long-period fluctuations of the geomagnetic field at AQU (H and D component; 1998 – 2002; ascending phase and solar maximum; $\Delta f \approx $ 0.7 – 5.0 mHz; $\delta f \approx $ 0.14 mHz) and concluded for a repeated occurrence of power enhancements at $f \approx $ 0.9 – 1.0, ≈ 1.2 – 1.4, ≈ 2.1 – 2.3, ≈ 2.5 – 2.7 mHz. Furthermore, they revealed a high coherence between the fluctuations in H, those in the magnetospheric field magnitude (GOES8, GOES10) and the compressive fluctuations in the SW (namely, $N_{SW}$ and IMF strength, with no correspondence with fluctuations of $V_{SW}$; Wind). Remarkably, most wave packets revealed an unprecedented one-to-one correspondence between SW and magnetospheric fluctuations in terms of frequency, onset, and duration (Fig. 7a). Additionally, a close agreement was achieved between the waveforms predicted for the modulation of the magnetopause current due to variable $P_{SW}$ and those observed in the magnetic field at the geostationary orbit and at ground-based stations (Fig. 7b). It clearly revealed that the magnetospheric/geomagnetic fluctuations were directly driven by $N_{SW}$/$P_{SW}$ fluctuations at the same frequencies.

- **IMP-8/AMPTE CCE/VIKING/EISCAT**
  On April, 23 – 24, 1987 (solar minimum), a unique alignment of the AMPTE CCE and Viking satellites with respect to the EISCAT (European Incoherent Scatter Scientific Association) Magnetometer Cross in Northern Scandinavia ($\theta _{a} \approx $ 64.0° – 67.8°) allowed Potemra et al. (1989) to conclude that an isolated $N_{SW}$ oscillation at $T \approx $ 8 – 10 min ($f \approx $ 1.5 – 2.1 mHz, IMP-8), persisting only for a couple of cycles, determined compressional magnetospheric oscillations at the same frequency. Interestingly, they possibly enhanced, in the auroral zone, resonant oscillations (already present) at approximately twice the frequency ($T \approx $ 4 min, $f \approx $ 4.2 mHz).
- **Wind/GOES**
  Kepko et al. (2002, #23) and Kepko and Spence (2003, #24) confirmed (Francia et al. 1999) a high correlation (R ≈ 0.9) between the time-shifted $P_{SW}$ fluctuations (Wind; 1996 – 2000; solar minimum and ascending phase) and the magnetospheric field in the dayside (mostly the $B_{z}$ component at GOES8 and GOES10; Fig. 6). In addition, the spectral analysis ($\Delta f \approx $ 0.1 – 5.0 mHz; $\delta f \approx $ 0.03/0.14 mHz) showed that, in several cases, the wave power spectra in the SW and in the magnetospheric field contained the same peaks at discrete frequencies ($f \approx $ 1.0, ≈ 1.2 – 1.5, ≈ 1.7 – 2.0, ≈ 2.4 and ≈ 2.7 mHz), often matching the CMS frequencies. They reported, however, also common peaks below 1.0 mHz ($f \approx $ 0.2, ≈ 0.3, ≈ 0.4, ≈ 0.6, ≈ 0.7 and ≈ 0.8 mHz). As a matter of facts, the $P_{SW}$ fluctuations might have directly driven magnetospheric fluctuations approximately at the same frequencies.

**Fig. 7:**
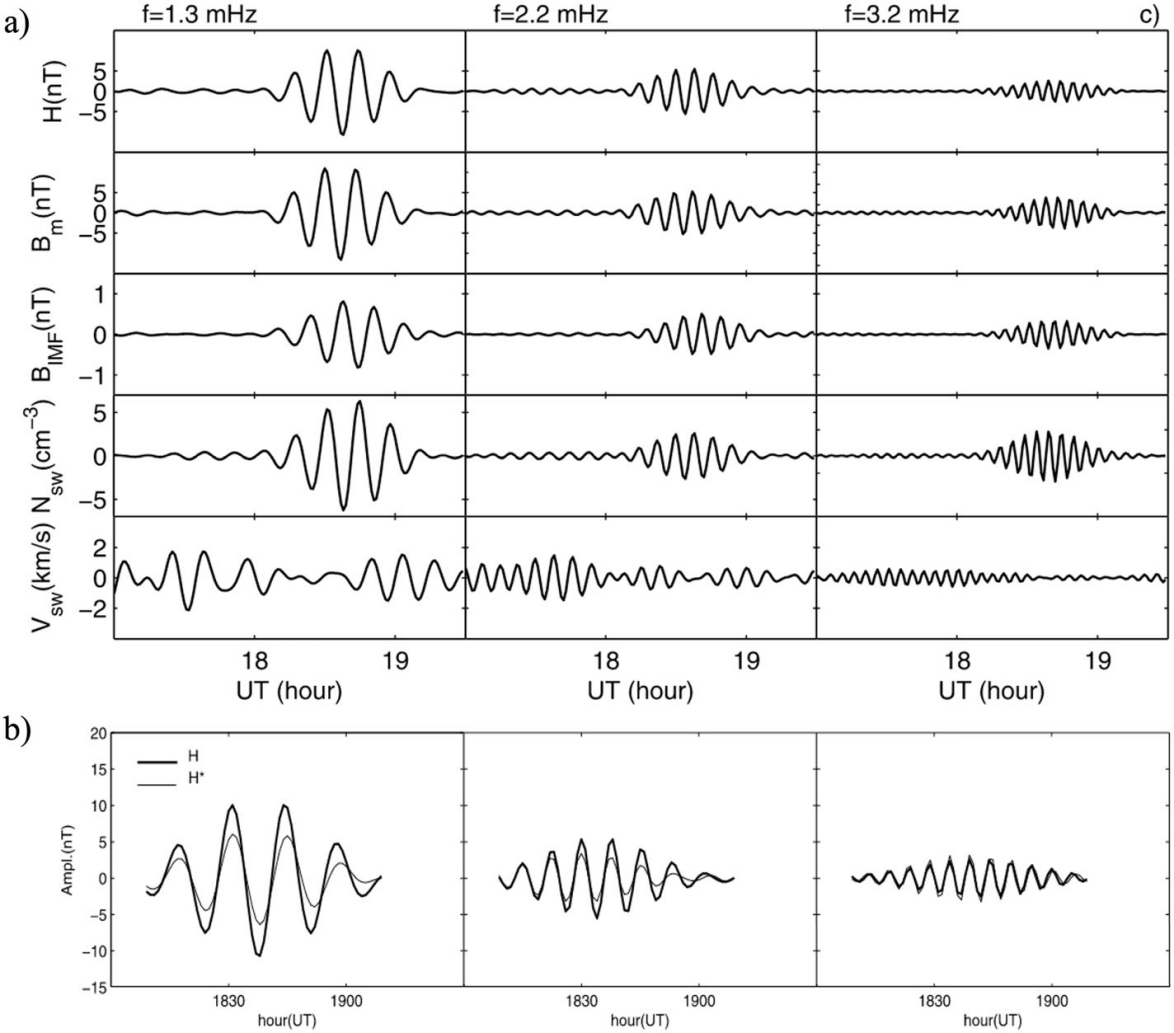
**A comparison between magnetospheric field and** **SW** **fluctuations.** (copyright by the American Geophysical Union, reproduced with permissions from Villante et al. 2007, #28). a) The comparison between filtered signals in three different frequency bands (bandwidth ± 0.25 mHz at half height). From the top: the H component of the geomagnetic field at AQU, the field strength at the geostationary orbit ($B_{m}$), the IMF strength ($B_{{IMF}}$), the SW number density ($N_{SW}$) and velocity ($V_{SW}$); August 1, 1998. b) The comparison between the filtered H signals (observed, thick trace) and those ones predicted by the modulation of the magnetopause current due to $P_{SW}$ variations ($H^{*}$; thin trace)
- **ACE/GOES/INTERMAGNET**
  Worldwide analysis of the geomagnetic field fluctuations at 76/77 INTERMAGNET stations, from equatorial up to $\theta \approx $ 80° in both hemispheres, during two periods of wave activity following the impact of two ISs on the magnetopause (March 27, 2001 and April 4, 2001; solar maximum), were conducted by Villante et al. (2013, #36) ($\Delta f \approx $ 0.3 – 8.0 mHz; $\delta f \approx $ 0.14 mHz) and by Villante et al. (2016, #40) ($\Delta f \approx $ 0.5 – 7.7 mHz; $\delta f \approx $ 0.52 mHz). In these cases, the magnetospheric field measurements at each data point along the geostationary orbit (GOES8, GOES10) were rotated in the Mean Field Aligned (MFA) coordinates ($b_{\mu}$ along the average field, as defined by the $N$ min vector running average; $b_{\varphi}$, perpendicular to $b_{\mu}$ and to the spacecraft position vector, directed eastward; $b_{\nu}$ completing the orthogonal system) in which the mean field was defined by a 20 min vector average. The analysis of the event discussed by Villante et al. (2013) revealed several signals at discrete frequencies ($f \approx $ 1.0, ≈ 1.9, ≈ 2.2 – 2.4, ≈ 2.6, ≈ 3.4 – 3.6, ≈ 4.7 mHz). The power enhancements detected in the magnetosphere and, practically ubiquitously, at ground-based instruments found correspondence in $P_{SW}$ power enhancements, approximately at the same frequencies (actually, power enhancements at nearby frequencies were observed also in the $V_{SW}$ and IMF components). The event proposed by Villante et al. (2016) was associated with a conspicuous $P_{SW}$ jump (determined by an IS) that, combined with the erosion of the magnetospheric field due to the southward IMF, caused a remarkable inward motion of the magnetopause and a relevant magnetospheric compression. The analysis revealed that fluctuations at the same discrete frequencies ($f \approx $ 1.5 – 1.8, ≈ 3.3 – 3.6, ≈ 4.4 – 4.6, and ≈ 5.9 – 6.2 mHz) were detected at the geostationary orbit in the morning and the dawn sector (measurements in other sectors were not available), and, ubiquitously, at ground-based stations. As for the previous case, all these fluctuations revealed a one-to-one correspondence with those ultimately identified in the HVS following the shock wave (basically, $P_{SW}$, although fluctuations were also present in other SW/IMF parameters).
- **Geotail/TNB/SuperDARN**
  A comparative study of ULF measurements at TNB and SuperDARN observations in the southern hemisphere, De Lauretis et al. (2016, #41) identified a wave event observed at TNB at $f \approx $ 2 mHz ($\Delta f \approx $ 1.0 – 7.0 mHz; $\delta f \approx $ 0.28 mHz) around local MLT noon, when the station approaches the dayside cusp (March 27, 2013, approaching solar maximum). It showed a counter clockwise polarization in the local morning and a clockwise polarization in the afternoon, as expected for a FLR excited at a latitude few degrees lower than TNB. SuperDARN observations reported the simultaneous occurrence of ionospheric fluctuations at the same frequency ($\theta _{a} \approx $ 75°S – 76°S, morning and pre-noon region) that were interpreted in terms of FLRs. $P_{SW}$ oscillations at the same frequency were detected by Geotail, just upstream of the morning flank of the bow shock, suggesting that the FLRs were driven by SW oscillations transmitted into the magnetosphere. In addition, the authors also speculated the possible direct propagation of SW waves along open field lines (Villante et al. 2001). At TNB, these waves were accompanied by less evident fluctuations at $f \approx $ 3.3 mHz along both the H and D component in the pre-noon sector and only along the D component in the afternoon sector. At SuperDARN, additional broadband fluctuations at $f \approx $ 3.0 – 4.0 mHz were observed.

#### Statistical Analyses

- **Wind/GOES**
  Viall et al. (2009, #31) examined the SW (Wind) and dayside magnetospheric fluctuations (GOES7, GOES8, GOES10) at discrete frequencies over 11 years (1995 – 2005; across the solar maximum 2001 – 2002; $\Delta f \approx $ 0.5 – 5.0 mHz; $\delta f \approx $ 0.05 mHz). They found that the $N_{SW}$ oscillations occurred more often at certain frequencies ($f \approx $ 0.7, ≈ 1.4, ≈ 2.0, and ≈ 4.8 mHz; see also Viall et al. (2008)). Similarly, an analysis of the $B_{z}$ component of the magnetic field at the geostationary orbit, within one hour of local noon, revealed more frequently occurring frequencies such as $f \approx $ 1.0, ≈ 1.5, ≈ 1.9, ≈ 2.8, ≈ 3.3, and ≈ 4.4 mHz. In addition, the comparison of the data sets showed that in ≈ 54% of the SW segments with a spectral peak, at least one of the same discrete frequencies was statistically significant in the corresponding magnetospheric data segment.
- **Geotail** **and ground-based observations**
  Geotail observations over six years (1995 to 2000; mostly ascending phase) in the outer magnetosphere (≈ 9 – 30 $R_{E}$) were analyzed by Kokubun (2013, #35), who visually selected 330 wave packets in plasma flow and magnetic field elements on the basis of amplitudes of fluctuations in the plasma velocity greater than ≈ 30 km s^−1^. More frequent during higher $V_{SW}$ conditions, these events revealed a pronounced dawn-dusk asymmetry with long duration events often observed in the morning sector (particularly around dawn) and rarely occurring in the afternoon sector; in addition, very few events were observed around noon. After rotating the magnetic field measurements at each point along the spacecraft trajectory in the MFA coordinates (the vector running average was over ≈ 25.4 min; $\Delta f \approx $ 0.3 – 10.0 mHz; $\delta f \approx $ 0.32 mHz), a number of preferential frequencies emerged in the morning sector around $f \approx $ 1.9, ≈ 2.5, ≈ 3.3 mHz and, possibly, ≈ 4.5 mHz, while dominant frequencies did not emerge in the afternoon sector. Several events were also compared with ground-based magnetic field observations performed at 30 stations ($\Delta f \approx $ 0.3 – 8.3 mHz; $\delta f \approx $ 0.32 mHz) in both hemispheres between $\theta _{a} \approx $ 73.2°S and $\theta _{a} \approx $ 77.5°. For the reported cases, as well as for the Geotail statistical results, the dominant frequencies (more clearly detected at $|\theta _{a}|> \approx $ 65°) were $f \approx $ 1.9, ≈ 2.1 – 2.2, ≈ 2.5 – 2.6, ≈ 3.1 – 3.5, ≈ 3.8 – 4.3 mHz.
- **ACE/GOES**
  Di Matteo and Villante (2017, #43) analyzed the $P_{SW}$ fluctuations (1998 – 2008) in the leading edges of HVS following 201 ISs, observed by ACE, and found events commonly selected by two different analytical methods (“common” events, more details in Sect. 7) in 185 streams. Their frequency distribution ($\Delta f \approx $ 1.2 – 4.9 mHz; $\delta f \approx $ 0.24 mHz) revealed more relevant percentages at $f \approx $ 1.7 – 1.9, ≈ 2.7 – 3.4, and ≈ 3.9 – 4.4 mHz (with the most relevant peak at $f \approx $ 4.2 mHz). With a similar analysis, Di Matteo and Villante (2018, #44) found “common” wave events in 107 out of 124 magnetospheric field intervals following SIs (GOES8 and GOES10) with higher percentages at $f \approx $ 1.5 – 1.7, ≈ 2.2 – 2.4, ≈ 3.9 – 4.2 mHz, and, more explicitly, at $f \approx $ 4.2 – 4.7 mHz. Out of 14 spectacular events, selected with extreme thresholds, three occurred at $f \approx $ 2.9 mHz and four at $f \approx $ 4.2 mHz. These results might suggest, in general, a correspondence between the frequencies observed in the leading edges of HVS and those detected in the magnetosphere. Nevertheless, a comparison conducted on pairs of corresponding SW/magnetospheric intervals revealed that a clear correspondence between the frequencies of the external and internal fluctuations is achieved only in a subset of events (Viall et al. 2009). On the other hand, the analysis of some relevant events suggested that while the SW compressional fluctuations drove magnetospheric modes approximately at the same frequencies (Kepko et al. 2002; Kepko and Spence 2003; Villante et al. 2007, 2013, 2016), in another cases, the absence of SW fluctuations at similar frequencies suggested magnetospheric fluctuations triggered by the magnetospheric compression or by other processes related to the IS impact. In addition, magnetospheric modes with the same characteristics at GOES8 and GOES10 were observed only in a few cases, suggesting that global waveguide/cavity modes might occur rarely.

### The Role of Fluctuations of Other SW Parameters

As previously remarked, some papers reported the possible relationships between magnetospheric fluctuations and waveforms observed not only in $N_{SW}$/$P_{SW}$ but also in other SW parameters (e. g., Villante et al. 2013). These aspects have been more explicitly highlighted in a number papers.

**Fig. 8:**
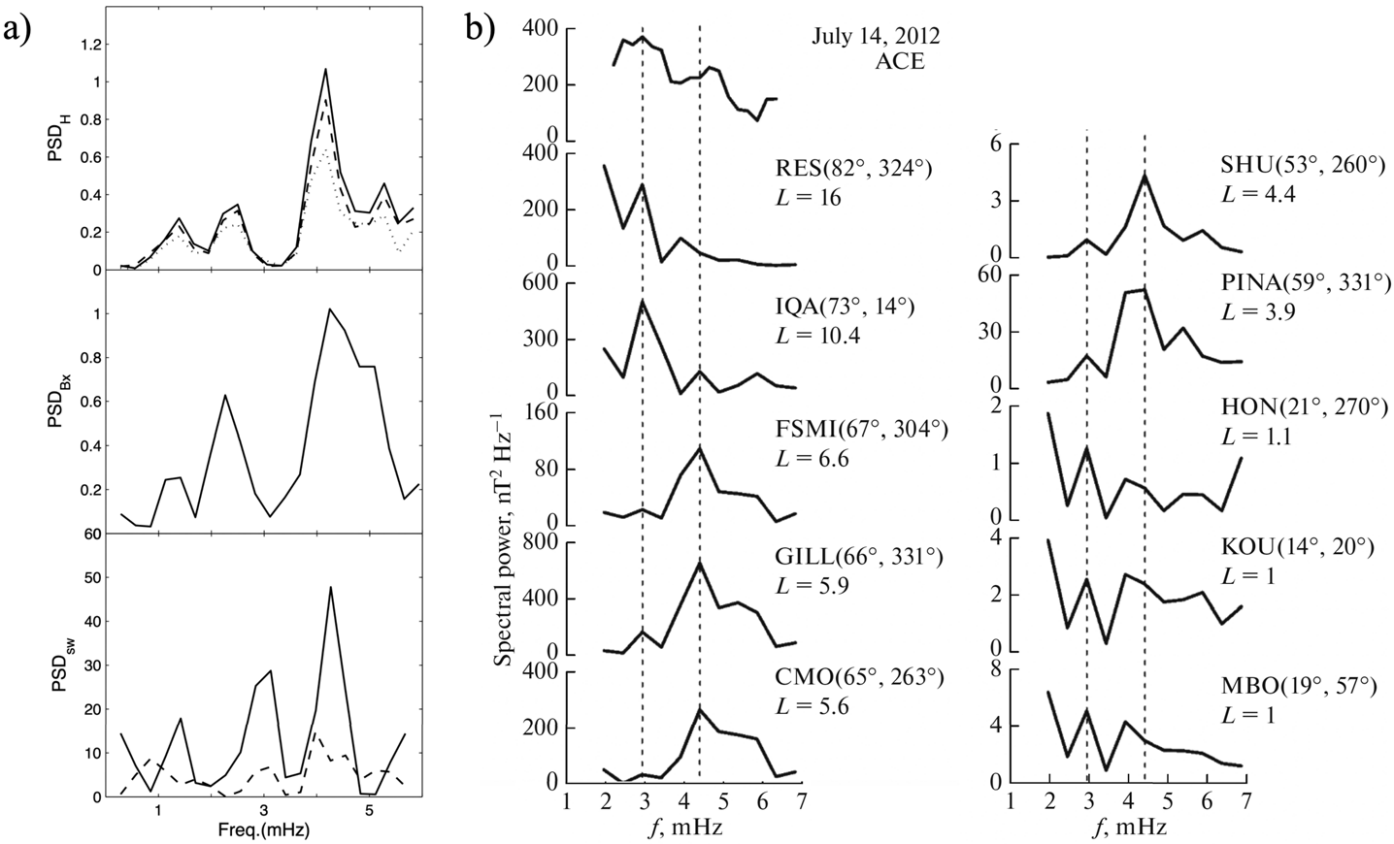
**A comparison between geomagnetic field and** **SW** **fluctuations.** a) (copyright by the authors, reproduced with permissions from Villante et al. 2006, #27). Spectral analysis of the wave event occurring on April 3, 2004, 14:18 – 15:18 UT. Top plot, the comparison between the power spectra of the geomagnetic field H component at three stations of the SEGMA array organized by decreasing latitude (NGC, $\theta _{a}\approx $ 42.8°, solid line; CST, $\theta _{a}\approx $ 40.8°, dashed line; RNC, $\theta _{a}\approx $ 38.3°, dotted line). Central plot, the power spectrum of the IMF radial component ($B_{X}$). Bottom plot, the power spectra of $V_{SW}$ (solid line) and $N_{SW}$ (dashed line); SW data have been time shifted by 1 h. b) (copyright by the Pleiades Publishing Ltd, reproduced with permissions from Klibanova et al. 2016, #42). From the top, the power spectrum of the IMF radial component and the ground magnetic field observations (north-south component) at high, middle and low latitudes
- **Wind/ACE/ground-based networks**
  Klibanova et al. (2016, #42) examined the Pc5 fluctuations ($\Delta f \approx $ 1.7 – 6.7; $\delta f \approx $ 0.56 mHz) observed after the SSC of July 14, 2012 (ascending phase) determined by the shock front observed by Wind and ACE. They analyzed magnetic field measurements from 34 stations at: the CANMOS (CANadian Magnetic Observatory System) and CARISMA array (Canadian Array for Realtime Investigations of Magnetic Activity; $\theta _{a} \approx $ 54° – 82°); American midlatitude (SHU, $\theta _{a} \approx $ 53°; SIT, $\theta _{a} \approx $ 60°) and low-latitude stations (KOU, MBO, HON, SJG; $\theta _{a} \approx $ 15° – 28°); the IMAGE array ($\theta _{a} \approx $ 55° – 72°); and KAK ($\theta _{a} \approx $ 22°; Japan). Relevant geomagnetic fluctuations occurred at $f \approx $ 2.9 mHz, predominantly at polar cap latitudes (open field lines) but also present at low latitude with smaller amplitude (Fig. 8b). As in Villante et al. (2006), apparently, they were associated with fluctuations at the same frequency of the IMF radial component, observed soon after the shock front and penetrating into the magnetosphere. Additional fluctuations at $f \approx $ 4.4 – 4.7 mHz, globally observed at all latitudes in the dayside sector, were interpreted as the manifestation of radial magnetopause oscillations.

- **Wind/IMP-8/Geotail/GOES/SuperDARN/CANOPUS**
  Prikryl et al. (1998, #16) reported a series of observations on January 24, 1996 (solar minimum), in the SW, magnetosheath, magnetosphere, HF radar and ground-based magnetometers (Wind, IMP, Geotail, GOES8; Stokkseyri/SuperDARN, Iceland, FOV: $\theta \approx $ 68° – 85°; $L \gtrsim $ 7.1; CANOPUS; $\Delta f \approx $ 0.8 – 4.0 mHz, $\delta f \approx $ 0.13 mHz). In this case, Wind observed large amplitude IMF fluctuations with dominant periodicities of ≈ 12 and 20 min ($f \approx $ 0.8 and ≈ 1.3 mHz) superimposed to fluctuations on scale of hours. Subsidiary components were detected at $f \approx $ 1.7, ≈ 2.0, ≈ 2.4, ≈ 2.9 mHz. Similar fluctuations were observed in $P_{SW}$ which also showed a remarkable oscillation at $f \approx $ 1.1 mHz. Approximately 20 min later, similar waveforms were detected in the post-noon magnetosheath (electric and magnetic field), in the magnetic field components at the geostationary orbit, in the ionospheric and ground-based measurements. According to the authors, the lower frequency compressional SW fluctuations caused compression and rarefaction in the dayside magnetosphere, while the higher frequency IMF oscillations excited FLRs on magnetic shell adjacent the noon magnetopause.
- **Wind/ACE/SHARE** **radar**
  Some investigations examined the correspondence between the SW observations and the fluctuation events detected in the Antarctic ionosphere from the SHARE radar. Stephenson and Walker (2002, #22) discussed an event occurred on April 28-29 1997 (solar minimum; $\Delta f \approx $ 1.0 – 5.0 mHz, $\delta f \approx $ 0.05 mHz) in the post-midnight sector and revealed that simultaneous oscillations were seen in the radar measurements and in several SW parameters (Wind; $V_{SW,x}$ $P_{SW}$, $N_{SW}$, $B_{x}$, $B_{y}$, $B_{z}$) near the CMS frequencies at $f \approx $ 1.3, ≈ 1.9, ≈ 2.7 and ≈ 3.3 mHz. However, they also remarked the occurrence of many other signals at other frequencies, with approximately the same power. Stephenson and Walker (2010, #34) analyzed the event of June 7, 2000 (two other events showed similar features; solar maximum; $\Delta f \approx $ 1.5 – 4.0 mHz; $\delta f \approx $ 0.15/0.17 mHz) and revealed a high degree of phase and amplitude coherence between the FLRs at $f \approx $ 1.9 mHz and, particularly, at $f \approx $ 2.1 mHz in the radar measurements and the fluctuations of several SW parameters (mostly, $V_{SW}$; ACE).
- **ACE/SEGMA**
  Villante et al. (2006, #27) analyzed the geomagnetic field observations at SEGMA (South European Geomagnetic Array; $\theta _{a} \approx $ 33.4° – 42.8°; $L \approx $ 1.5 – 1.9; $\Delta f \approx $ 0.3 – 8.0 mHz; $\delta f \approx $ 0.3 mHz) during a geomagnetic storm (April 3, 2004; descending phase) and identified in the power spectra of the H component at all stations common enhancements at $f \approx $ 1.4, ≈ 2.2 – 2.5 mHz and, more explicit, at ≈ 4.2 mHz, all of them with amplitude increasing with latitude (Fig. 8a). Remarkably, peaks at the same frequencies were detected in the IMF radial component $B_{x}$, while the $V_{SW}$ spectra showed enhancements at $f \approx $ 1.4 and ≈ 4.2 mHz, with a smaller peak at $f \approx $ 3 mHz. In this case, much smaller enhancements appeared in the spectra of $N_{SW}$ at $f \approx $ 0.8, $f \approx $ 3.0 and 4.0 mHz. It suggests geomagnetic waves possibly driven by SW non-compressive fluctuations. Interestingly, an analysis of the time interval in which the peak at $f \approx $ 4.2 mHz more clearly emerged in the SW spectra revealed a strong correlation between $B_{x}$ and $V_{SW,x}$ (R ≈ 0.92) and a small ratio between the variance of the IMF strength and the total variance of the field components (R ≈ 0.03), suggesting the Alfvénic character of these fluctuations.

## Role of Analytical Methods and Spacecraft Position

As shown in the previous paragraphs, a great variety of analytical methods has been adopted for the evaluation of the power spectra (see the supporting information Table S1 for a detailed list). This feature and the related aspects (the interval length, the data sampling rate, the frequency range, the frequency resolution, etc.) and the different criteria for the identification of the relevant events (from visual selection to highly sophisticated methods) may significantly influence the analysis of the fluctuations at discrete frequencies and the comparison between different investigations.

**Fig. 9:**
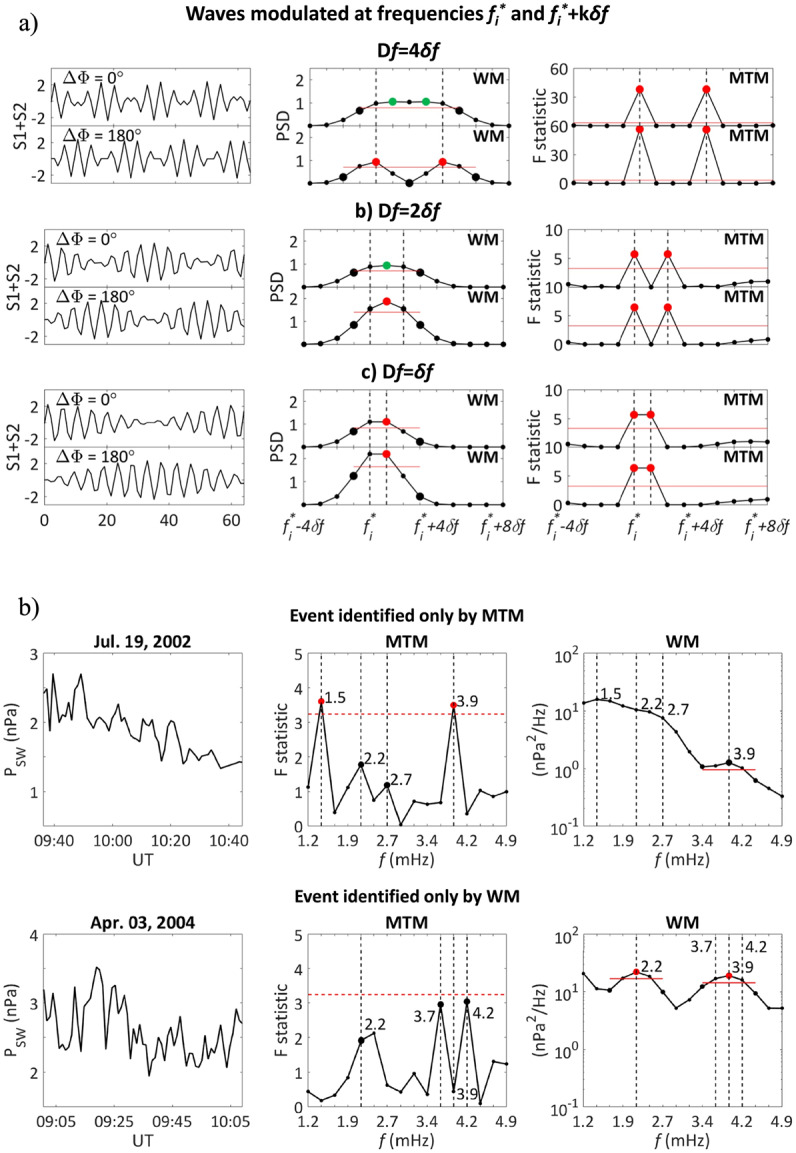
**Some critical aspects of the spectral analysis.** a) (copyright by the American Geophysical Union, reproduced with permissions from Di Matteo and Villante 2017, #43). The results of the frequency estimate and event identification obtained by the WM and MTM analysis for signals composed of two sinusoids S1 and S2, respectively at frequency $f_{i}^{*}$ and $f_{i}^{*} + k\delta f$ ($\delta f$ being the spectral resolution). The top panels correspond to $k = 4$, the middle panels to $k = 2$, and the bottom panels to $k = 1$. The results are proposed for initial phase difference between signals $\Delta \phi =$ 0° and $\Delta \phi =$ 180°. On the left panels, the composite signals; in the central panels, the WM spectra; in the right panels the results of the MTM analysis. The black dashed vertical lines identify the real signal frequencies; the red horizontal lines identify the thresholds for the event identification for WM and MTM, respectively; the red (green) points identify the selected (unselected) peaks. Depending on methods and signal characteristics, the analysis can provide wrong frequency estimates and erroneous event identification. b) (after Di Matteo and Villante 2017, #43). Two examples of events identified either by MTM or by WM for given thresholds. The experimental $P_{SW}$ data (left panels); the results of the MTM analysis (central panels); the WM spectra (right panels). The black dashed vertical lines identify the frequencies of the peaks identified either by WM or MTM. The red lines represent the threshold for the selection of MTM and WM events. The red (black) dots identify the selected (unselected) peaks. In (a) the events are identified only by MTM; in (b) only by WM

A first analysis in this sense was conducted by Di Matteo and Villante (2017) who compared the performances of two methods adopted in the scientific literature for the analysis of periodic variations: the Welch method (WM) and the Multitaper method and F-test (MTM). Considering synthetic signals, they showed that the identification of the wave occurrence and the frequency estimate may be strongly influenced by the signal characteristics and noise level, with MTM possibly more efficient than WM. It is worth noting that, in presence of multi-component signals, WM may erroneously identify modes at intermediate (wrong) frequencies between two real ones (Fig. 9a). On the other hand, a critical aspect is represented by the agreement/disagreement between different methods in the identification of a signal at the same frequency bin. Interestingly, considering real cases, Di Matteo and Villante (2017) presented two examples in which, adopting automatic criteria, periodic $P_{SW}$ variations would have been selected either by MTM (case a, in Fig. 9b) or by WM (case b). In addition, by examining 201 HVS, they revealed that the WM/MTM agreement was achieved only in ≈ 50% of cases (“common events”). Di Matteo and Villante (2018) extended the analysis to the magnetospheric fluctuations following 124 SIs and confirmed that, as in the SW, the WM/MTM agreement was achieved for ≈ 50% of the events. These results make clear that different analytical methods might provide, for the same data set, different statistical results (eventually contaminated by spurious identifications) and different sets of favorite frequencies.

**Fig. 10:**
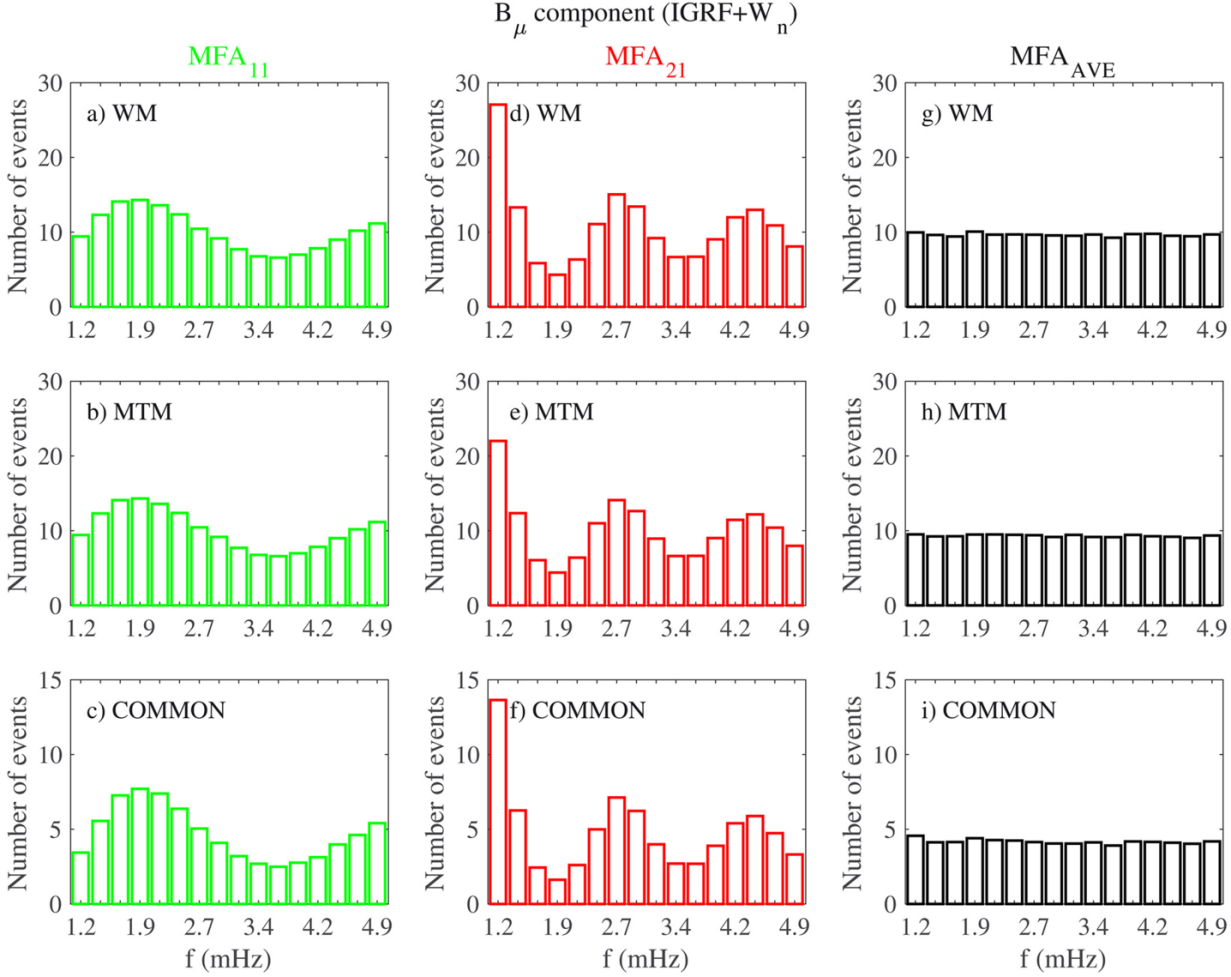
**The influence of rotation in** **MFA** **coordinates.** (copyright by the American Geophysical Union, reproduced with permissions from Di Matteo and Villante 2018, #44). The frequency distributions of the spurious events identified in the $B_{\mu}$ component (along the average field) of the magnetospheric field after having rotated in the MFA coordinates (as determined by running averages) the data at each point along the geostationary orbit. In this case the results are obtained for 1-min International Geomagnetic Reference Field (IGRF) data, intermingled with white noise $W_{n}$ (in absence of signals). The left panels correspond to running averages over 11 data points, the central panel to averages over 21 data points, the right panel to an average over the entire examined interval (68 min interval). The three top panel show the results of the WM analysis, the middle panels, those of the MTM analysis, the bottom panels show the results obtained for (“common”) events selected by both methods. Spurious events preferentially occur at frequencies determined by the length of the running averages. By contrast, uniform distributions of spurious events are obtained when the average field is obtained on the entire interval

An additional problem arises in the magnetosphere where, to better identify the characteristics of the wave modes, the magnetic field measurements, at each point along the spacecraft trajectory, are often rotated in the MFA coordinates, as determined by the running averages of the experimental measurements. Di Matteo and Villante (2018) showed that this technique may determine a dramatic contamination in the event identification, with relevant consequences especially for (statistical) analyses in which the events are automatically selected. Indeed, simply considering the IGRF plus white noise representation of the magnetospheric field at the geostationary orbit, in absence of superimposed synthetic signals, both WM and MTM provided spurious identifications of events at frequencies strongly related to the length of the running average window (Fig. 10). We found interesting to evaluate here the expected effects of the rotation in MFA coordinates for observations at the geostationary orbit with the same parameters (i.e., interval length and data points concurring to the running average) adopted by Villante et al. (2013) and Villante et al. (2016) for the analysis of single events; spurious events would occur respectively at $f \approx $ 1.1, ≈ 2.8, ≈ 4.4, ≈ 6.0, ≈ 7.5 mHz and at $f \approx $ 1.0, ≈ 2.9, ≈ 4.4, ≈ 5.9, ≈ 7.4 mHz. In the statistical analysis conducted by Kokubun (2013), the possible contamination from spurious events could have occurred approximately at $f \approx $ 1.0, ≈ 2.2, ≈ 3.5, ≈ 4.8, ≈ 6.1 mHz.

As previously reported, the SW and magnetospheric observations for selected events are often compared with ground-based measurements. Nevertheless, this comparison may be contaminated by the different shape and slope ($\alpha $) of the power spectra in different regions, with ground and magnetospheric spectra typically steeper than those in the SW. Indeed, while the IMF and $V_{SW}$ components typically show, from ≈ 0.3 to ≈ 1 AU, $\alpha \approx -1.0/-1.7$ (Alfonsetti et al. 1998; Bruno and Carbone 2013; Treumann et al. 2019), the magnetospheric spectra typically show $\alpha \approx -2.0/-2.5$ (Ozeke et al. 2014; Pokhotelov et al. 2015). Meanwhile, a preliminary long-term analysis of the power spectra of the geomagnetic field components at low latitude (AQU; approximately two solar cycles; Colonico et al. 2020), revealed that, between $f \approx $ 1 – 5 mHz and depending on season, the spectra typically decrease with $\alpha $ ranging between $\approx -3.2$ and $\approx -2.1$, with an average $\alpha \approx -2.6$. Similar conclusions were obtained at extreme southern latitudes, in Antarctica, where, in the same frequency range, the power spectra of the geomagnetic field components, steeper during the summer, decreased on average with $\alpha \approx -2.9$ (Villante et al. 1997). Consistently, at high latitudes, the ground (Yagova 2015) and the magnetospheric spectra (AMPTE, GOES; Ozeke et al. 2012) were observed to decrease with $\alpha \approx -2.4/-2.8$ in the Pc5 range.

**Fig. 11:**
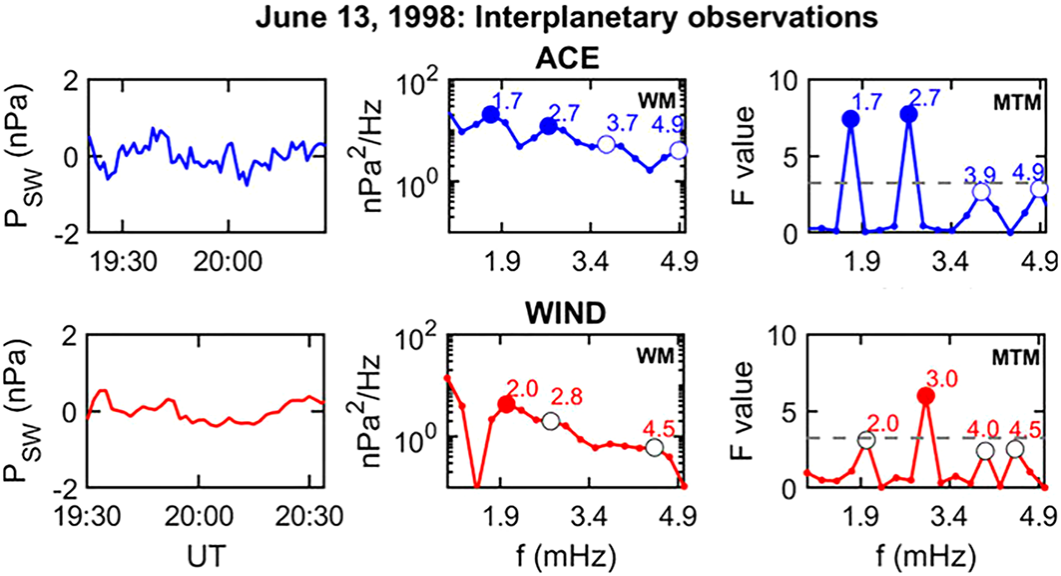
**The comparison between** **SW** **observations at different positions.** (copyright by the authors, reproduced with permissions from Villante et al. 2022, #49). The identification of events in $P_{SW}$ observed on June 13, 1988, by ACE (19:20 – 20:28 UT; top row) and Wind (19:41 – 20:49 UT; bottom row), both located in front of the magnetosphere. Left panels, the detrended $P_{SW}$ observations; central panels, the results of the WM analysis; right panels, the results of the MTM analysis. Filled and empty circles represent selected events and unselected peaks/enhancements, respectively. The horizontal line for MTM indicates the confidence threshold. This example demonstrates that results might be different when the SW is observed at different positions

Lastly, the analysis of the correspondence between SW and magnetospheric events require an unambiguous identification of the characteristics of the SW that effectively impact the magnetosphere. This is a long-standing limitation in studies of solar wind-magnetosphere coupling, as summarized by Di Matteo and Sivadas (2022). For this scope, it is important to understand if the SW characteristics are the same when observed at different places in front of the magnetosphere. In a recent analysis, Villante et al. (2022, #49) compared Wind and ACE observations for a period in which the spacecraft were separated along and perpendicular to the Sun Earth line by only $DX \approx $ 80 R_E_ and $DY \approx $ 40 R_E_, respectively, and showed a delay of $DT \approx $ 20 min. Note, for example, that for a wave frequency $f \approx $ 1.7 mHz, the wave period, $T \approx $ 9.8 min, would be ≈ 0.5 $DT$, and the wavelength, $\lambda \approx $ 35 R_E_ ≈ 0.44 $DX$. Despite these favorable conditions, the wave events selected at the two spacecraft and the corresponding frequencies were not the same (Fig. 11).

## Summary and Discussion

Earliest analyses of the JHU/APL HF radar observations at Goose Bay and of the geomagnetic field measurements at CANOPUS (six days, autumn/winter, solar maximum, post-midnight MLT sector) evidenced almost monochromatic fluctuations at different frequencies simultaneously occurring in a narrow latitudinal range (approximately between $\theta _{a} \approx $ 69° – 72°). These oscillations were originally interpreted in terms of FLRs driven by cavity/waveguide modes of the magnetosphere (and their harmonics), possibly reflected between the magnetopause and turning points outside the plasmasphere. The associated frequencies ($f_{1} \approx $ 1.3, $f_{2} \approx $ 1.9, $f_{3} \approx $ 2.6 – 2.7, and $f_{4} \approx $ 3.2 – 3.4 mHz) were identified as CMS (or “magic”) frequencies. The possibility that they would constitute a stable set of favorite frequencies has been discussed in the scientific literature over many years. Following these pioneering investigations, long term analyses at the same sites, revealed that similar fluctuations occurred through the entire year, in the dayside and in the dusk magnetosphere (although likely absent around noon), with a greater occurrence at higher latitudes. Further analyses, at other sites, revealed the (almost) simultaneous occurrence of several fluctuations at discrete frequencies as a common aspect from low up to extreme latitudes (with some evidence even in the polar cap).

**Fig. 12:**
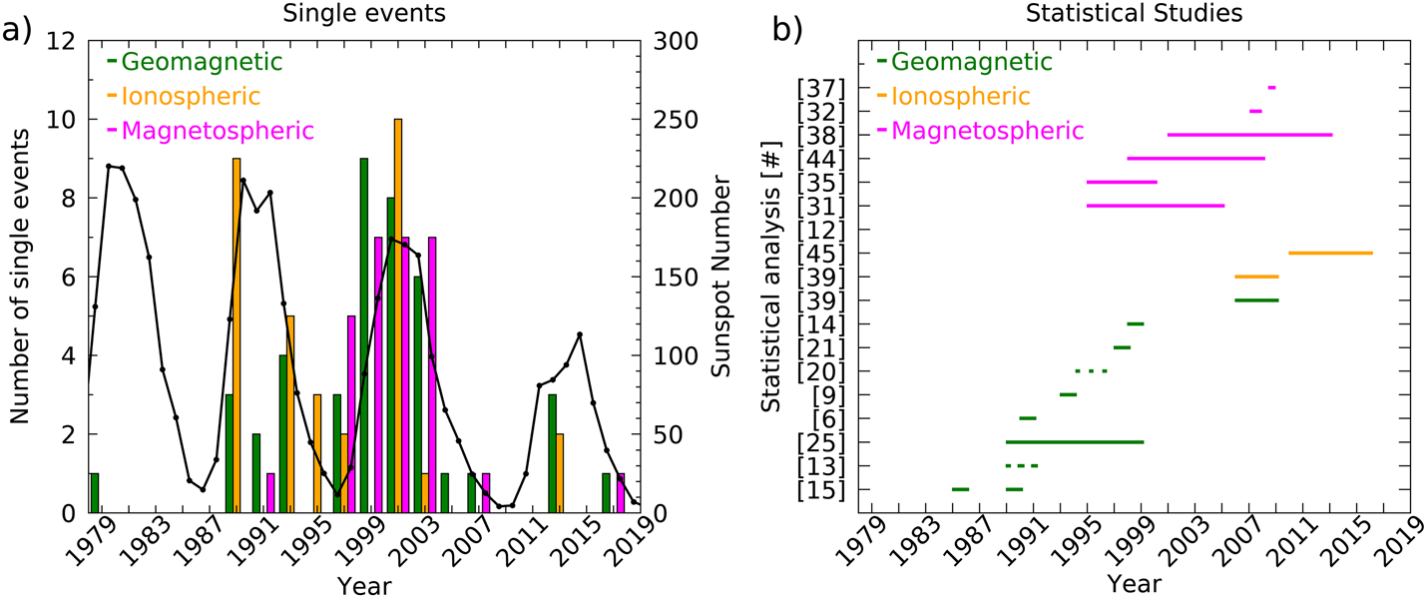
**Time intervals for single events and statistical investigations**. a) Time interval distribution for the single events compared with Sunspot Number (SILSO, World Data Center 1978-2018). b) Stack-plot of the time intervals analyzed in statistical investigations. Green, orange, and magenta pertain results from geomagnetic, ionospheric, and magnetospheric field observations, respectively. Number in brackets identify each investigation as defined in Table 1

**Fig. 13:**
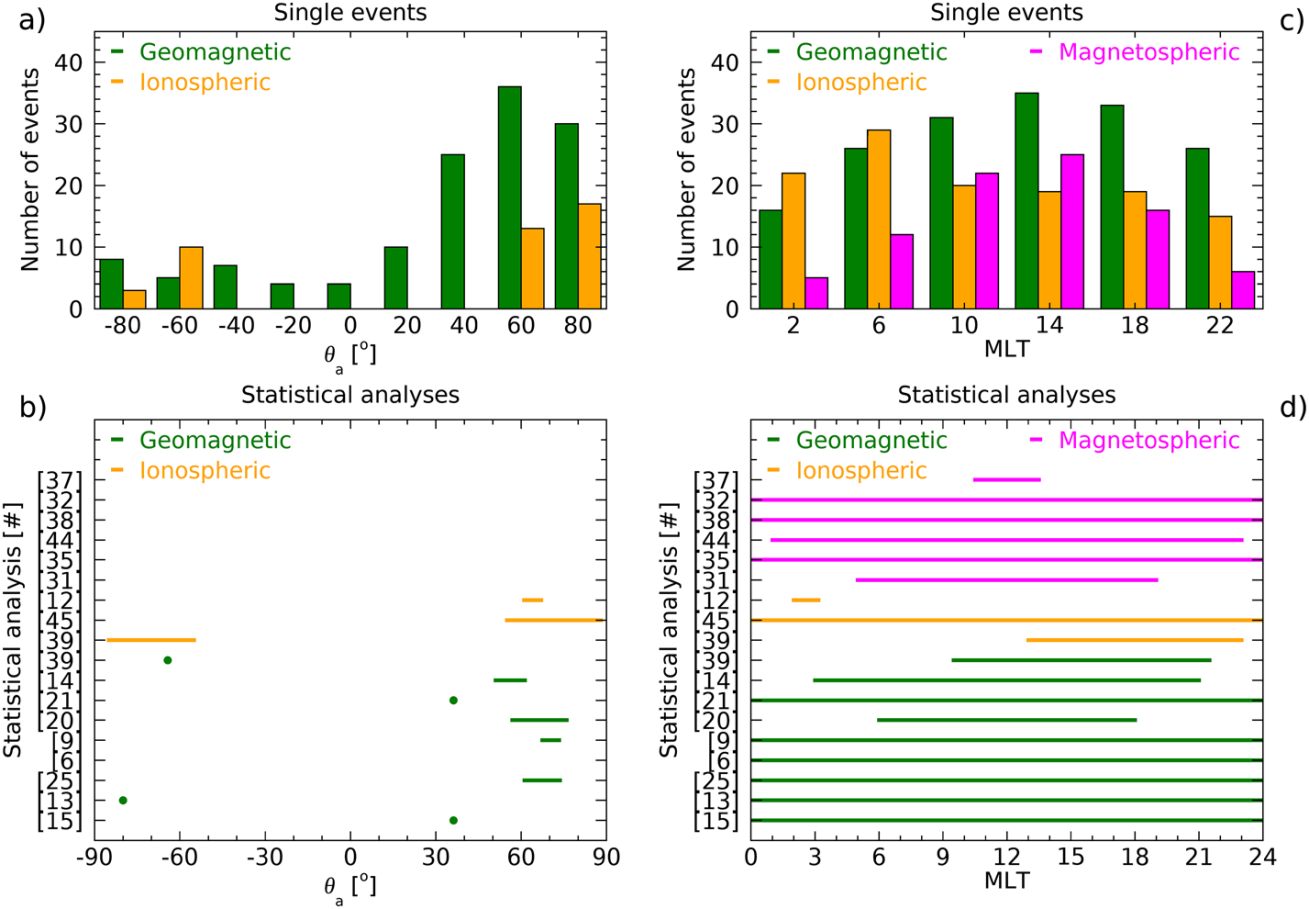
**Spatial distribution of the sites where waves at set of discrete frequencies were reported.** a) and b) Latitudinal distribution and stack-plot for case studies and statistical investigations, respectively. Dots in the stack-plot indicate analysis performed at a single station. c) and d) The same for the MLT distribution. Green, orange, and magenta indicate results from geomagnetic, ionospheric, and magnetospheric field observations, respectively. The latitudinal distribution for magnetospheric field studies is excluded since the information is often unavailable and dependent on the field line tracing methodology. Number in brackets identify each investigation as defined in Table 1

**Fig. 14:**
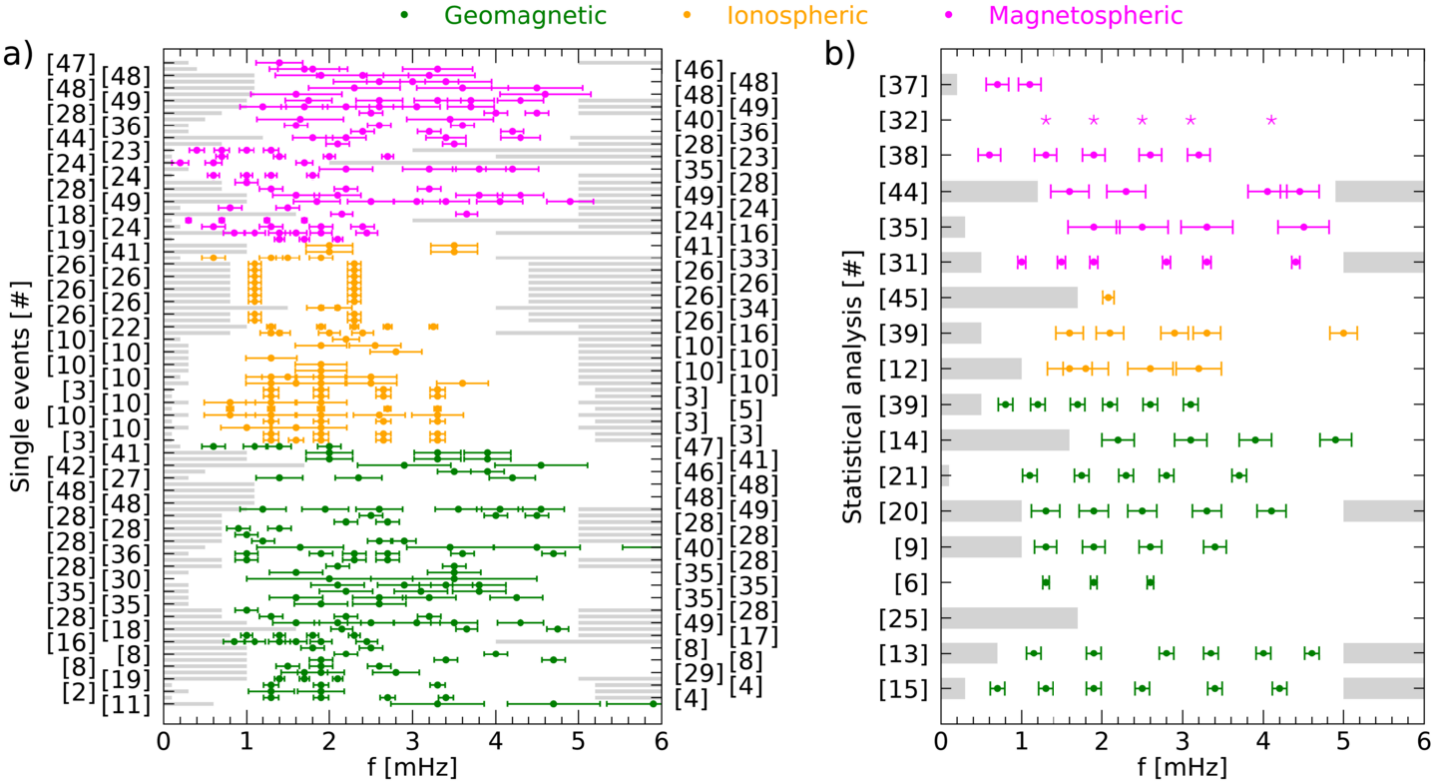
**Set of discrete frequencies for single events and statistical investigations.** a) and b) Stack-plot of the set of discrete frequencies reported in single events and statistical investigation, respectively. The gray bar, when present, delimit the reported frequency range analyzed in each investigation. Green, orange, and magenta indicate results from geomagnetic, ionospheric, and magnetospheric field observations, respectively. Number in brackets identify each investigation as defined in Table 1

**Fig. 15:**
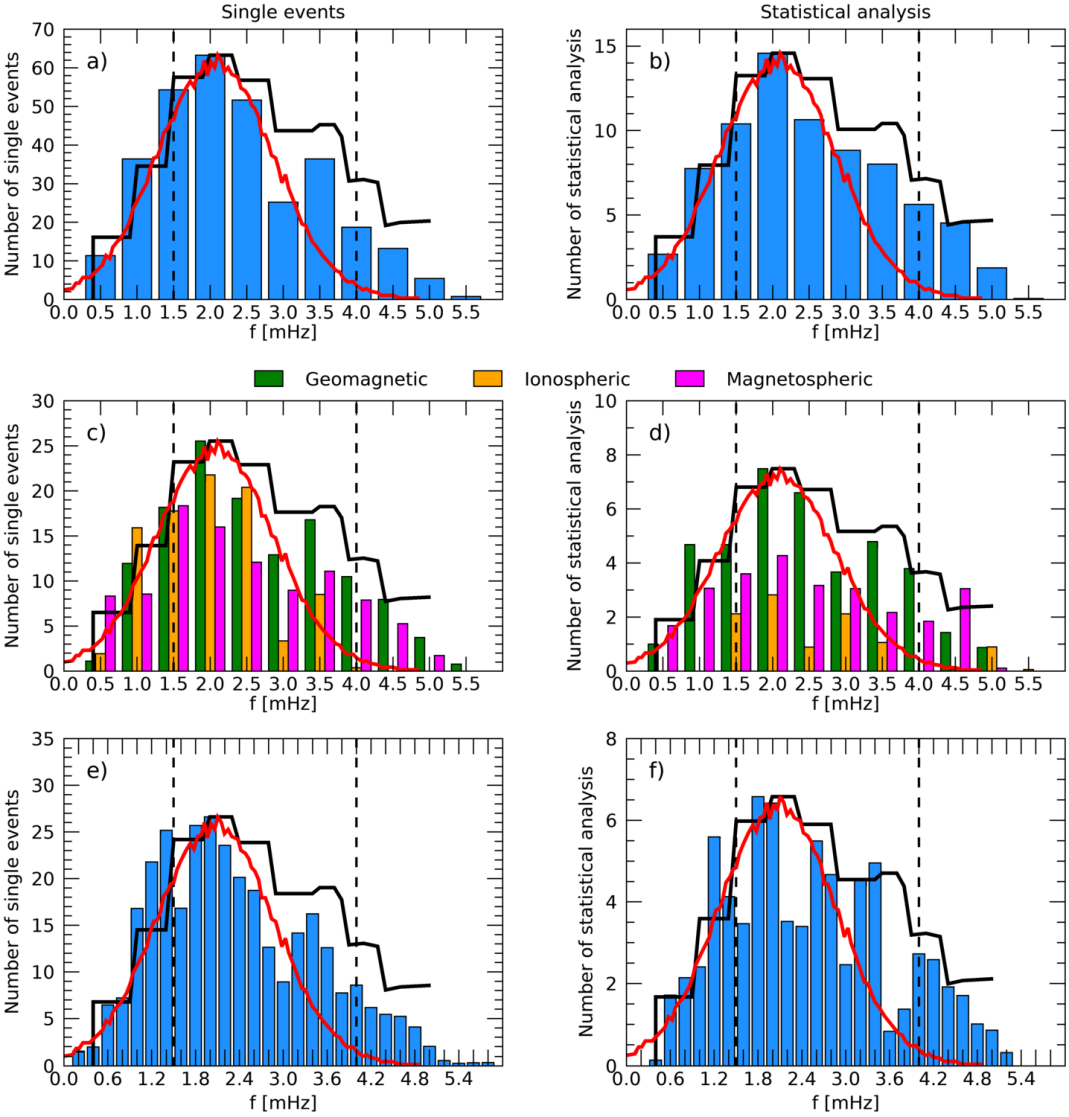
**Frequency distribution for single events and statistical analysis.** a) Global frequency distribution combining experimental results from geomagnetic (green), ionospheric (orange), and magnetospheric (magenta) investigations which are depicted separately in panel c); bin width is 0.5 mHz. b) and d) The same but for statistical investigation in which we counted as a single entity the occurrence of events in each frequency band in the single analysis, independently on the actual number of the concurring events or percentage (unweighted histograms). Dashed vertical lines delimit the frequency range that was most frequently analyzed in the reviewed investigations. e) and f) same as a) and b) but with 0.2 mHz bin width. Red lines, modeled frequency distribution (not to scale) of periodic *N*_*SW*_ structures by (Kepko et al. 2024); black lines, occurrence rate (not to scale) of periodicities in transverse velocity fluctuations in the solar corona (Morton et al. 2019)
- **The relation between magnetospheric and** **SW** **variations**. The occurrence of periodic variations in SW parameters have been frequently observed during periods in which waves at a set of discrete frequency are observed in the magnetosphere, indeed:
  - Highlighting a close similarity between the $P_{SW}$ and the H traces in the dayside sector, Francia et al. (1999) clearly evidenced that the quasi-periodic variations of $P_{SW}$ ($f \lesssim $ 5 mHz) are rapidly transmitted to the magnetosphere, slightly altering the magnetospheric size and/or modulating the magnetopause current. In this sense, they extended the conclusions by Sibeck et al. (1989) who documented, for a single event, that remarkable and quasi-periodic variations of $P_{SW}$ and IMF strength, with period $T \approx $ 8 – 10 min ($f \approx $ 1.6 – 2.6 mHz), found correspondence in compressional fluctuations of the dayside magnetospheric field and geomagnetic field at *South Pole* (close to MLT local noon). In the subsequent years, evidence has been progressively accumulated in favor of a close correspondence between the sets of fluctuations in $N_{SW}$/$P_{SW}$ and those in the magnetospheric and geomagnetic field, approximately at the same frequencies, in a range extending from $f \approx $ 0.2 up to $f \approx $ 5.9 mHz (Prikryl et al. 1998; Kepko et al. 2002; Kepko and Spence 2003; Stephenson and Walker 2002; Eriksson et al. 2006b; Villante et al. 2007; Fenrich and Waters 2008; Liou et al. 2008; Viall et al. 2009; Stephenson and Walker 2010; Villante et al. 2013, 2016; De Lauretis et al. 2016; Di Matteo and Villante 2018; Villante et al. 2022; Di Matteo et al. 2022). Note that, as we discuss in the following, the bands below $f \approx $ 1.0 mHz have been more rarely examined and waves within $f \approx $ 5.0 – 7.0 mHz not routinely considered. Villante et al. (2007) found a spectacular correspondence between the SW and the magnetospheric fluctuations in terms of frequency, onset and duration, as well as a close agreement between the waveforms predicted for the modulation of the magnetopause current and those observed at the geostationary orbit. Assuming a similar origin from the lowest to the highest frequencies, this correspondence suggests that the compressive structures of the incoming SW may alter the shape and size of the magnetospheric cavity leading to the appearance of compressional fluctuations of the magnetospheric field, approximately at the same frequencies. As underlined by Kepko et al. (2002), in these cases, the magnetospheric field increases or decreases to balance the corresponding $P_{SW}$ fluctuations, through a “forced breathing mode” of the entire magnetosphere.
  - Interesting results in this context were provided by Kepko et al. (2020) who conducted a statistical analysis of the $N_{SW}$ “mesoscale” structures (1995 – 2015; Wind spacecraft; see also Viall et al. 2008) with interesting results. The distribution of the occurrence of the estimated radial length scales ($L_{x}$) of these SW structures showed enhancements near $L_{x}\approx $ 130 – 140 Mm and $L_{x} \approx $ 170 – 190 Mm, in the slow SW ($V_{SW} <$ 550 km s^−1^), while bands near $L_{x}\approx $ 230 and $L_{x}\approx $ 300 Mm were commonly observed in both the slow and in the fast SW ($V_{SW} >$ 550 km s^−1^). The proposed favorite $L_{x}$ are comparable to the dimensions of the dayside magnetosphere, consequently, when the SW structures impact the magnetopause they may drive variations of the magnetospheric field up to frequencies corresponding approximately to the fast MHD wave travel time through the magnetosphere ($T_{t} \gtrsim $ 3 min). It follows that the frequency distribution of the magnetospheric fluctuations might show enhancements clustered around the frequencies determined by the length (and speed) of the advected SW structures. In this sense, the structures observed during slow SW would determine frequencies around $f \approx $ 3.0 and ≈ 2.3 mHz while those commonly observed during both slow and fast SW would determine frequencies around $f \approx $ 1.9 and ≈ 1.2 mHz (slow SW), and $f \approx $ 3.1 and ≈ 2.0 mHz (fast SW). In conclusions, the impact of the “mesoscale” SW structures on the magnetosphere would lead to predict frequencies such as $f\approx $ 1.2, ≈ 1.9–2.0, ≈ 2.3 and ≈ 3.0–3.1 mHz which attain the range of frequencies in which the magnetospheric fluctuations are commonly observed. Remarkably, in this sense, the more persistent peak emerging at $f \approx $ 1.9 – 2.0 mHz (Fig. 15) might be due to its association with both slow and fast SW structures impacting the magnetosphere. A similar argument might hold for fluctuations at $f\approx $ 3.0–3.1 mHz, less persistent in the experimental spectra which rather show a clear enhancement of events at somewhat higher frequencies ($f\approx $ 3.5 mHz). In this context, the peaks at $f\approx $ 1.2– 1.4 mHz and $f\approx $ 2.4–2.6 mHz, emerging in the analysis at higher frequency resolution (Fig. 15e-f), might be tentatively related to $N_{SW}$ structures corresponding to $f\approx $ 1.2 mHz and $f\approx $ 2.3 mHz pertaining only the slow SW. Interestingly, according to a recent analysis (Kepko et al. 2024), the preferential frequencies of the quasi-periodic density structures (PDS) observed by Wind (also identified in the variations of the alpha-to-proton number density ratio) occur superimposed on a significant continuous occurrence rate profile extending between $f\approx $ 1 and ≈ 3.0 mHz, and centered near ≈ 2.1 mHz. Kepko et al. (2024) were able to reproduce the observed distribution by assuming that ≈ 30% of the SW segments contain a PDS according to a Gaussian distribution (red lines in Fig. 15; not to scale). This distribution, in turn, showed similarities with the occurrence rate of periodicities in transverse velocity fluctuations in the solar corona (black lines, Fig. 15) observed by the Hinode Coronal Multi-channel Polarimeter (Morton et al. 2016; Tomczyk et al. 2007; Morton et al. 2019). Based on these observations and simulations, Kepko et al. (2024) suggested the likely solar origin of these *N*_*SW*_ structures.
  - Several investigations considered in the present review, focusing attention on the Pc5 characteristics, did not explore the frequency band below $f \approx $ 1.7 mHz (e. g., Chisham and Orr 1997; Baker 2003; Klibanova et al. 2016). Consequently, events at lower frequencies are more rarely reported in the results summarized in the previous paragraphs. In addition, it is worth noting that FLRs at these frequencies might be expected only at the highest latitude stations. This aspect was specifically addressed by Provan and Yeoman (1997) who compared the spectra at Goose Bay and Wick and concluded that “*…the frequencies of FLRs are almost identical despite the different latitudinal range covered by the two radars, …the Wick radar does not detect pulsations at frequencies as low as 1.3 mHz, …the geomagnetic field lines at Wick are too short to support waves of this low frequency*”. All these aspects might then concur to explain the low occurrence of events at $f \approx $ 1.2 – 1.3 mHz. Nevertheless, several investigations, which examined wider frequency ranges, reported several events at frequencies between $f \approx $ 0.2 – 1.0 mHz (Walker et al. 1992; Fenrich et al. 1995; Francia and Villante 1997; Prikryl et al. 1998; Mathie and Mann 2000; Kepko et al. 2002; Kepko and Spence 2003; Lessard et al. 2003; Eriksson et al. 2006b; Villante et al. 2007; Mthembu et al. 2009; Villante et al. 2013; Archer et al. 2013; Norouzi-Sedeh et al. 2015; He et al. 2020; Birch and Hargreaves 2020; Alimaganbetov and Streltsov 2020; Di Matteo et al. 2022; Nykyri et al. 2024). In the proposed scenario, these events might be tentatively associated with the SW structures with $L_{x} \geq \approx $ 500 Mm reported by Kepko et al. (2020) and $V_{SW} \approx $ 300 – 350 km s^−1^. Interestingly, Lessard et al. (2003), who examined the occurrence of lowest frequency fluctuations in the energetic particle flux (and, less visible, in the magnetic field data) at the geostationary orbit, reported the presence of a discrete spectrum of oscillations below $f \approx $ 1 mHz that were typically observed on a global scale and persisting for several hours or more. They also concluded in favor of magnetospheric fluctuations likely driven by SW variations. However, similar magnetospheric waves might also be triggered by other processes, such as the modulated magnetic reconnection at the magnetopause, due to the fluctuating IMF orientation in particular SW structures (Prikryl et al. 1998) as well as by Alfvénic fluctuations embedded in HVS (Villante et al. 2006). In this context, interesting results were obtained by Alimaganbetov and Streltsov (2020) who analyzed the SW fluctuations registered by ACE and the ground fluctuations observed at high, middle, and low latitudes during 84 intense substorm events and reported geomagnetic fluctuations, with approximately the same frequency at all latitudes, most frequently at $f \approx $ 0.45 – 0.55 and $f \approx $ 0.75 – 0.80 mHz. Remarkably, during these active periods, the waves correspondence with the fluctuations in $N_{SW}$ was not as good as that one with the fluctuations in the IMF components (typically, with greater amplitude along $B_{x}$). In general, the amplitude of fluctuations at low latitudes was higher than at middle latitudes, suggesting that, during similar periods, the geomagnetic fluctuations below ≈ 1 mHz might also be generated by the disturbances in the ring current driven by the IMF fluctuations. Finally, we remind that standing Alfvén waves below $f \approx $ 1.0 mHz can also occur during unusual conditions of the magnetosphere system, e. g., during abnormally expanded state (Mathie and Mann 2000). In addition, more recently, Nykyri et al. (2024) proposed a new nightside generation mechanism in which the lunar wake, during the Moon transit through the Earth’s magnetotail, may excite magnetic field fluctuations in the lowest frequency range of the magnetospheric discrete frequency waves.
- **The relation with the cavity/waveguide modes**. Events at discrete frequencies might still be related (at least occasionally) to cavity/waveguide modes at frequencies changing with the changes of the magnetospheric characteristics (Baker 2003; Hartinger et al. 2023a). In general, however, the power spectra of the magnetospheric fluctuations were found to be more complex than expected often exhibiting, since earliest investigations (e. g. Walker et al. 1992), peaks at frequencies lower and higher than the CMS frequencies, that cannot be reconciled with the cavity/waveguide model predictions. Indeed, in the cavity/waveguide scenario, events below $f \approx $ 1.0 mHz would require an outer magnetospheric boundary at (unrealistic) geocentric distances greater than ≈ 30 R_E_ while events above $f \approx $ 3.5 mHz would be rarely expected for a reflecting boundary located above the plasmapause. On the other hand, as reported by Di Matteo and Villante (2018), who analyzed magnetospheric field measurements following the impact of ISs (when global cavity/waveguide might be expected), oscillations modes with the same characteristics at different LTs are not commonly observed at the geostationary orbit. These results would suggest an occurrence of the global waveguide/cavity modes rarer than expected (Hartinger et al. 2013). A possible way to relate the “magic” frequencies and the MHD eigenmodes is to investigate the expected relationships of the wave frequency with solar activity and latitude. As previously remarked, the magnetosphere’s mass density, changing over a solar cycle, might determine a modulation of the frequency of standing Alfvén waves, resulting in lower values at solar maximum and higher values at solar minimum. In Fig. 16 we report the results which might be obtained sorting the set of discrete frequencies resulting from single events by the solar activity as represented by the sunspot (Wolf) number. Figure 16a shows the results obtained from geomagnetic observations at all latitudes: clearly, there is no apparent relationship between the reported frequencies and the sunspot number. In our opinion, this result does not imply that MHD eigenmodes are not involved in the occurrence of ULF waves at discrete frequencies. Rather, it supports our suggestions that the critical aspects we remark in this work play a major role: indeed, the results in Fig. 16a are at least contaminated by the latitudinal variation of the FLR frequencies. Therefore, we repeated the analysis selecting stations in a narrower latitudinal range (the chosen values are such to allow a reasonable number of points in the scatter plot; Fig. 16b). Again, there is no evident relation between frequencies and sunspot number. This feature might reflect another of the critical aspects. Indeed, both latitudes and frequencies are affected by the different criteria adopted in different investigations: indeed, some works report the latitude where the wave reaches its maximum amplitude, other analyses report the latitudinal range where the wave’s amplitude is above a certain threshold, while some others identify the wave occurrence regardless of its amplitude. Obviously, these inconsistencies between different investigations create ambiguities which contributed to the current controversy on this subject. Regarding the change of the standing Alfvén wave frequency with latitude, we remind that one characteristic of the waves at a set of discrete frequencies is their simultaneous observations at various locations. Considering this aspect, many investigations provide the latitudes where the waves were observed without providing specific information about the latitude where the amplitude of the wave at a given frequency reaches its maximum value (marking the latitude of the field line resonating). Without this information, the obtained occurrence distributions might well provide a misleading picture. Figure 16c-d show the occurrence number of single events in terms of frequency and latitude. Taking in account the uncertainties in the wave frequency and the reported latitudinal extension of the event occurrence (it also depends on the criteria used to identify the event), we determined the total number of cases falling into bins in latitude and frequency defined by a step of 0.05 mHz mHz and 0.5°, respectively. Actually, to obtain a correct distribution we should normalize the events occurrence by the number of intervals analyzed in each investigation at all available stations (but, in general, this information is not available). From the geomagnetic results in Fig. 16c, we can only conclude that waves at frequencies between 1 and 5 mHz mostly occur in the latitudinal range $65^{\circ}<\theta _{a}<75^{\circ}$, where they match the typical FLR frequencies. The ionospheric results in Fig. 16d (which consists of few measurements) show some indication of the latitudinal dependence of the observed discrete frequencies with the occurrence of lower frequencies spreading toward higher latitude while the higher frequencies are concentrated around $\theta _{a}\approx 70^{\circ}$. These results confirm the need of more detailed analyses in which the critical aspects highlighted in the present paper are carefully examined.

In the present paper, we conducted a comprehensive review of many investigations related to these aspects: globally, our review includes the results of 103 single events and those of 17 statistical investigations. The experimental measurements were performed in different regions: magnetosphere (presented in magenta in the following figures), ionosphere (orange), and at several ground-based stations (green). As shown in Fig. 12a, although the analysis extended between 1978 – 2018, most of the single events were identified during solar cycle 23 (August 1996 – December 2008). Particularly, more than 50% of them (55) occurred between 1998 – 2004. Consequently, the reported results should be considered mostly indicative of solar maximum conditions. By contrast, some statistical studies (globally extending between 1985 – 2015, Fig. 12b) spanned time intervals comparable to a solar cycle (Baker (2003), 1989 – 1999; Viall et al. (2009), 1995 – 2005; Di Matteo and Villante (2018), 1998 – 2008; Archer et al. (2013), 2001 – 2013). Note also that the analyses conducted in different regions often do not overlap the same time intervals. Figure 13a-b show that the geomagnetic events were mostly observed above $\theta _{a}\approx $ 50° in both hemispheres; the same occurs for ionospheric events. The magnetospheric events are excluded in this figure since the information is often unavailable and dependent on the field line tracing methodology. In the northern hemisphere, where most observations were performed, the peak percentage of geomagnetic events was observed around $\theta _{a}\approx $ 60°. The MLT distributions (Fig. 13c) show a predominant occurrence of geomagnetic and magnetospheric events in the wide dayside sector (≈ 06 – 18 MLT). The magnetospheric events were mostly observed around noon (≈ 10 – 14 MLT). Although observed through the entire day, the ionospheric events were more frequently observed in the night hours (≈ 00 – 06 MLT). Figure 14 shows the reported set of discrete frequency for single events (panel a) and statistical analyses (panel b). Note that the explored frequency ranges, limited by the grey bars, were not the same. Unfortunately, in several papers the upper limit of the analyzed frequency range is not reported, but it is likely that they did not extend $f\approx $ 5 mHz). From an analysis of the global results, we suggest the following conclusions:

- **The stability of the** **CMS** **frequencies**. In our opinion, although, as we discuss in next paragraphs, some frequencies appear more often than others and the events typically tend to cluster in some frequency intervals, a global analysis of the magnetospheric, ionospheric and geomagnetic oscillations do not allow to conclude for the existence of a stable and persistent *absolute* set of favorite frequencies; indeed:
  - The originally proposed CMS frequencies were the central ones of quite large bands (in general, $\delta f \approx $ 0.32 mHz) and their variability was likely greater than the one proposed in those investigations. For example, according to Walker et al. (1992) (*“frequency constant to better than 10% over a LT period of nearly 4 hours”*) the frequencies of the proposed events might have fluctuated, in few hours, approximately in bands at least large as $Df_{1}\approx $ 1.2 – 1.4, $Df_{2}\approx $ 1.7 – 2.1, $Df_{3}\approx $ 2.4 – 2.9 and $Df_{4}\approx $ 3.0 – 3.6 mHz, practically covering ≈ 70% of the range $Df \approx $ 1.2 – 3.6 mHz. On the other hand, several other papers (for example, Ziesolleck and McDiarmid 1994) remarked that, at the same stations, the geomagnetic signals often experienced *“a considerable temporal variation in the signal frequency not only within a particular LT sector but also from day to day”*.
  - The wave frequency is more clearly identified in the radar data than in geomagnetic measurements since the latter integrates signals from a significant area of the ionosphere. This aspect was examined by Ziesolleck et al. (1998) who compared FLR events observed simultaneously by SuperDARN and CANOPUS and concluded that although, in general, ionospheric radars and ground-based magnetometers observed the same event with very similar spectral characteristics, in some cases, the estimated frequencies (and their spatial variations) were significantly different, an aspect possibly due, at least at high latitudes, to the broadband electrojet noise superposed on the geomagnetic measurements.
  - The analyses have been conducted adopting different methods for the event identification (from highly sophisticated to visual selection) and for the estimate of the corresponding frequency. As clearly evidenced by Di Matteo and Villante (2017) and Di Matteo and Villante (2018), the agreement between two different methods for the event identification might be only of the order of ≈ 50% (both in the SW and in the magnetosphere). Additionally, for multicomponent signals, wrong estimates of the wave frequency might be provided at intermediate frequencies between the real ones of periodic components (interestingly in this sense, Ziesolleck and McDiarmid (1995), reported a peak of preferential frequencies *“near 3.0 mHz lying right in between the two CMS frequencies of 2.6 and 3.4 mHz”*). Additional problems arise, when the magnetospheric field measurements are rotated in the MFA coordinates using running averages to determine the ambient magnetic field. This rotation approach may determine a tremendous bias (particularly in statistical analyses) with the identification of spurious events at frequencies determined by the length of the examined intervals (Di Matteo and Villante 2018). Mitigation of this effect includes the use of the same window length for the estimate of the ambient magnetic field and the power spectrum (Di Matteo and Villante 2018) or the use of alternative approaches (e. g., Regi et al. 2017).
  - Several papers examined the experimental observations over long term intervals and with high spectral resolution to compare the estimated frequencies with the proposed CMS frequencies (for example, at CANOPUS: *“a peak near 2.0 mHz lied in the vicinity of the 1.9 mHz CMS frequency”;* Ziesolleck and McDiarmid 1995) and to investigate the possible occurrence of a persistent set of favorite frequencies. In general, these analyses concluded that the set of the CMS frequencies did not appear particularly distinguished from other sets. On the other hand, due to the short (daily) and the long term (over a solar cycle) variation of $f_{r}$, stable frequency sets should not be expected for FLRs at a given site. Rather, at different LTs, FLRs at the same frequency should be expected at different sites, following the latitudinal, LT and solar cycle dependence of $f_{r}$. In addition, in several cases (e. g., Villante et al. 1998; Lepidi et al. 1999; Villante et al. 2001, 2013, 2016), the same frequencies were simultaneously detected at large latitudinal separation, an aspect hard to reconcile with the proposed FLR pattern, predicting, for a peak at a given frequency, no more than 1° – 2° in latitudinal extent. It is also worth noting, in this context, that the comparison between different investigations should request a careful attention to the SW conditions. Indeed, the impact of ICME, ISs and $P_{SW}$ fronts may determine huge changes of the magnetospheric size and characteristics (with respect to more quiet SW conditions), with relevant variations of the local $f_{r}$ as well as of the cavity/waveguide modes eigenfrequencies.
  - In addition to previous arguments, it is worth noting that, in general, the criteria adopted for selecting events may deeply influence the comparison of the results obtained by different investigations (even at the same station). The different criteria for the identification of events (“long lasting”; “observed at several stations”; “highly polarized”; “FLRs”, etc.) might pre-determine the selection of classes of events having different origin and characteristics. For example, Boulet (1992), at CANOPUS, did not request any coherence among signals of the geomagnetic field components. By contrast, at the same array, Ziesolleck and McDiarmid (1995) focused on highly-polarized waves, even with low power, systematically excluding from their analysis possible large amplitude non-polarized or weekly-polarized waves. Similarly, the visual selection of Pc5 events at CANOPUS, conducted by Baker (2003), was tailored toward strong monochromatic and highly polarized signals with multicomponent intervals (≈ 19% of identified events) excluded in the polarization analysis and in the subsequent distinction in FLRs and non-FLRs events. In our opinion, an example of the bias toward specific classes of events determined by the selection criteria can be recognized in Fig. 2a in which the event occurrence reported by Baker (2003) is skewed toward high latitudes showing a MLT dependence typical of FLRs. Observations at lower latitudes are, instead, significantly less frequent and show, in the dayside, a more uniform distribution in MLT, similar to that one typical of non-FLR events. On the other hand, with decreasing latitude, $f_{r}$ progressively increases toward the Pc4/3 range (Mathie et al. 1999a) and the occurrence of discrete frequency events at lower frequencies might be related to evanescent compressive modes able to penetrate deep into the magnetosphere.
- **The aspects of the frequency distribution**. The analysis of the frequency distributions of events provides interesting results. The reviewed investigations applied various methodologies and approaches to identify the set of discrete frequencies; consequently, different uncertainties were associated to the frequency estimates. Therefore, to obtain a global frequency distribution, for an event with uncertainty interval $\Delta f$, covering $n$ frequency bins, each including a portion $\Delta f_{i}$ of $\Delta f$, we assigned a fraction of event ($\Delta f_{i}/\Delta f$) to the corresponding bin $i$, where $i=1, \dots , n$, so that $\Sigma _{i} \Delta f_{i}/\Delta f =1$. The resultant distribution corresponds to the total number of events. Figure 15 shows the resulting histograms as organized in bins of ±0.25 mHz, centered at 0.5 mHz, 1.0 mHz, 1.5 mHz …(different organizations provided similar results). Note that the events repeatedly examined in the scientific literature, such as those in the pioneering investigations, have been considered only once, with the best frequency resolution. As seen in panel a, the global frequency distribution of the single events reveals, in the range of frequency explored by most investigations (dashed lines), a strong predominance of cases between $f\approx $ 1.5–2.5 mHz, with percentages maximizing in the bin centered at $f = {2.0}\text{ mHz}$ (a feature mostly due to events occurring at $f\approx $ 1.9 mHz, Table 1) and rapidly decreasing with increasing frequency. Evidence for an additional peak emerges at $f = {3.5}\text{ mHz}$. The same analysis conducted separating for different regions (panel c; contaminated by a smaller statistic) reveals that these aspects are explicit in the geomagnetic measurements, showing sharp peaks at $f = {2.0}\text{ mHz}$ (appearing also in the ionospheric data) and at $f = {3.5}\text{ mHz}$, while the magnetospheric distribution reveals decreasing percentages between $f=1.5$–3.0 mHz and a minor increase at $f = {3.5}\text{ mHz}$. Panel b and d show the global histograms obtained collecting the results of all statistical analysis; in these histograms we counted as a single entity the occurrence of events in each frequency band in the single analysis, independently on the actual number of the concurring events or percentage (unweighted histograms). The global histogram in panel b confirms the peak at $f = {2.0}\text{ mHz}$. In this case, the separate analyses (panel d) reveal this peak in all regions; in addition, they show an enhancement at $f = {3.5}\text{ mHz}$ in the geomagnetic data and a small enhancement at $f = {3.0}\text{ mHz}$ in the ionospheric measurements. The comparison between the histograms in Fig. 15 may be influenced by the poor statistics, by the unweighted histograms of the statistical investigations, by the extension of some of them to entire solar cycles as well as to various latitudes and MLT of the experimental observations. However, they undoubtedly reveal a peak of events at $f\approx $ 1.9–2.0 mHz and a percentage of events around $f\approx $ 3.5 mHz more relevant than at nearby frequencies (more explicitly for geomagnetic events). We found also interesting to repeat the global analysis for smaller frequency bins (0.2 mHz, Fig. 15e-f). Both distributions, in addition to the peak at $f\approx $ 1.8–2.0 mHz, show enhancements at $f\approx $ 1.2–1.4, ≈ 3.2–3.6 mHz and, for the statistical analysis, also at $f\approx $ 2.6–2.8 mHz (i.e., in substantial agreement with the ranges of the so-called CMS frequencies). While suggestive, these results need to be further investigated with more extensive and consistent investigations (i.e., taking in consideration the bias and limitation we have discussed through this review). In fact, we note that by using the results without considering the uncertainties, we would obtain a more scattered distribution (not shown) which remarks the low statistical significance due to the low number of events in narrower frequency bins.

**Fig. 16:**
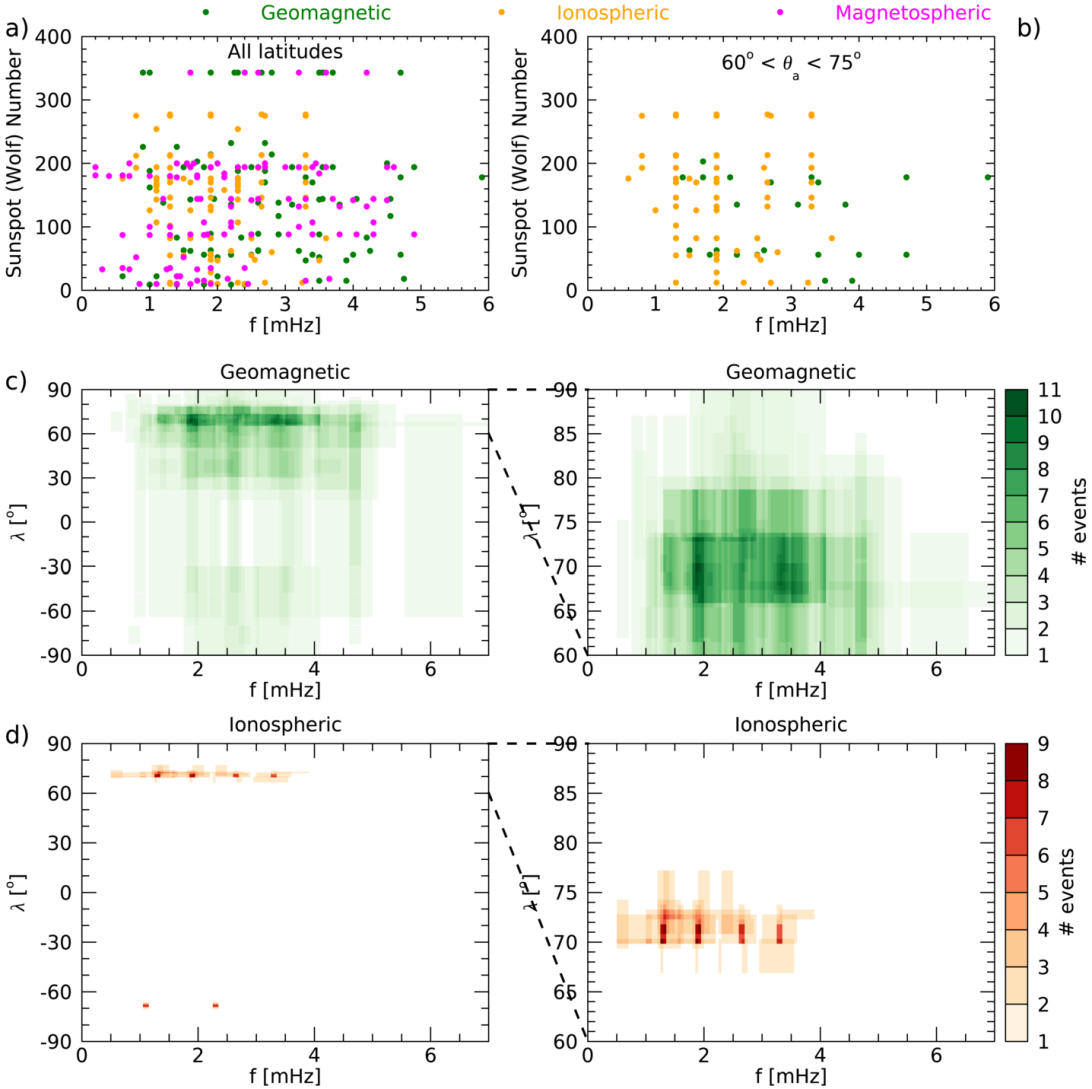
**Set of discrete frequencies for single events in function of solar activity and latitude.** a) Scatter plot of sunspots number versus frequency identified in single event analysis. Uncertainties in frequency are not reported for clarity. b) Same as a) but from observations in a narrower latitudinal range. c) Occurrence number of single events in terms of frequency and latitude from geomagnetic observations. d) Same as c) from ionospheric observations
- **The relation with surface modes**. Among other generation mechanisms, particular attention has been dedicated to the role of the magnetopause motions and surface waves on magnetospheric boundaries. In this sense, Plaschke et al. (2009a, #32) (see also Plaschke et al. 2009b), examined the possible occurrence of magnetopause motions activated by $P_{SW}$ fluctuations buffeting the non-rigid magnetopause and perturbing the Chapman-Ferraro current system. Considering 6697 crossings of the five Time History of Events and Macroscale Interactions (THEMIS) spacecraft (April – Sept. 2007, solar minimum), they obtained a distribution of the magnetopause oscillation frequencies with enhancements at $f \approx $ 1.3, ≈ 1.9, ≈ 2.5, ≈ 3.1 and ≈ 4.1 mHz (bin size, $\delta f \approx $ 0.2 mHz), i.e., comparable to the CMS frequencies. These results were interpreted in terms of standing Alfvénic surface modes at the magnetopause eigenfrequencies (Kruskal and Schwarzschild 1954). Plaschke et al. (2009c) reported that these events might preferentially occur in the noon sector, during periods of northward IMF orientation, slow/moderate SW speed and low cone angle (between the Sun-Earth line and the IMF direction). Archer et al. (2013, #37) proposed fundamental frequencies of the magnetopause waves such as $f \approx $ 1.15 mHz for fast SW speed and $f_{S} \approx $ 0.7 mHz for slow SW speed and suggested that most of the CMS frequencies were likely due to surface waves and their harmonics. These aspects were further examined by Archer and Plaschke (2015, #38) who considered SW data spanning an entire solar cycle (2001 – 2013) and proposed a fundamental frequency of the magnetopause modes ranging between $f \approx $ 0.52 – 0.67 mHz, with a most probable value of $f \approx $ 0.64 mHz. More recently, Archer et al. (2019, #46) identified in satellite observations evidence of magnetopause surface modes at $f \approx $ 1.7 – 1.8 and ≈ 3.3 mHz, with possible signatures of the latter wave in ground magnetometer ($f \approx $ 3.5 ± 0.2 mHz). Additionally, He et al. (2020, #47) reported plasmapause surface wave modes at discrete frequencies, namely at $f \approx $ 1.4 mHz which was observed also at ground observatories, accompanied by additional fluctuations at $f \approx $ 0.6, ≈ 1.1, and ≈ 2.0 mHz. Note, however, that magnetohydrodynamic (MHD) surface modes on a plasma boundary appear to be highly localized at ground in latitude and local time, therefore difficult to reconcile with fluctuations at discrete frequency simultaneously observed along a wide range of latitudes and LTs. Additionally, the duration of surface waves, expected to be short lived (Kozyreva et al. 2019), questions their relation to the longer lasting discrete waves.
- **Differentiating waves at discrete frequencies due to different mechanisms**. As extensively discussed, set of magnetospheric waves at discrete frequencies can result from many external and internal generation mechanisms that can occur simultaneously, such as impacts of SW fronts and compressive fluctuations, FLRs, waveguide/cavity modes, MHD surface waves and SW “forced breathing”, among others. Consequently, as already remarked by Ziesolleck and McDiarmid (1995), the lack of a favorite set of frequencies could be related to: (i) the low occurrence rate of the related mechanism with respect others generating waves over a continuum in frequency, as well as (ii) the comparable occurrence rates between mechanisms generating waves at complementary sets of discrete frequencies. So far, the aspects possibly related to the concurring contributions of different generation mechanisms in the same frequency band have not been examined in the scientific literature. By contrast, extensive analyses of these aspects are needed to untangle the effects of multiple generation mechanisms on the occurrence of waves at sets of discrete frequencies. An attempt to discriminate between ULF waves of different origins has been recently conducted by Di Matteo et al. (2022, #48) who showed, for a time interval of ≈ 6 h (November 9, 2002; solar maximum), at the geostationary orbit and at ground stations, the appearance, in subsequent subinterval of ≈ 90 min, of ULF waves at sets of discrete frequencies covering most of the Pc5 frequency range, from ≈ 1.5 to ≈ 4.9 mHz, namely: $f \approx $ 1.5 – 1.6, ≈ 3.7, 4.6 mHz; $f \approx $ 2.3 – 2.4, ≈ 3.4 – 3.7 mHz; $f \approx $ 2.4 – 2.7, ≈ 2.9 – 3.1 mHz; $f \approx $ 1.8 – 1.9, ≈ 2.3 – 2.4, ≈ 3.1 – 3.2, ≈ 4.9 mHz. Despite occurring either simultaneously or right after one another, these waves were shown to be likely related to different generation mechanisms: shock impact, SW “forced breathing”, FLRs, internal instabilities. In conclusion, effort toward investigations discriminating between waves related to different mechanisms might shed new light on the occurrence of waves at sets of discrete frequencies.

## Supplementary Information

Below is the link to the electronic supplementary material.

Table S1 is provided as a supplementary spreadsheet including characteristics of magnetospheric waves at discrete frequencies, particularly: paper’s reference number, author/year, reported frequency, data and time of the single-event/statistical-analysis, instrument(s), sampling time, time interval length, frequency range, frequency resolution, location ($\theta _{a}$, MLT), pre-processing step, spectral analysis procedure, general comments. (XLSX 45 kB)

## Data Availability

Sunspot data are from the World Data Center SILSO, Royal Observatory of Belgium, Brussels (http://www.sidc.be/silso/).

## References

[CR1] Agapitov O, Glassmeier K-H, Plaschke F, Auster H-U, Constantinescu D, Angelopoulos V, Magnes W, Nakamura R, Carlson CW, Frey S, McFadden JP (2009) Surface waves and field line resonances: a THEMIS case study. J Geophys Res 114(A7):A00C27. 10.1029/2008JA013553

[CR2] Alfonsetti M, Vellante M, Villante U (1998) Power spectra of the solar wind parameters at low frequencies in the inner Solar System. Mem Soc Astron Ital 69:753

[CR3] Alimaganbetov M, Streltsov AV (2020) ULF waves observed in solar wind and on the ground at high, mid, and low latitudes. J Atmos Sol-Terr Phys 200:105220. 10.1016/j.jastp.2020.105220

[CR4] Archer MO, Plaschke F (2015) What frequencies of standing surface waves can the subsolar magnetopause support? J Geophys Res 120(5):3632–3646. 10.1002/2014JA020545

[CR5] Archer MO, Hartinger MD, Horbury TS (2013) Magnetospheric “magic” frequencies as magnetopause surface eigenmodes. Geophys Res Lett 40(19):5003–5008. 10.1002/grl.50979

[CR6] Archer MO, Hartinger MD, Walsh BM, Plaschke F, Angelopoulos V (2015) Frequency variability of standing Alfvén waves excited by fast mode resonances in the outer magnetosphere. Geophys Res Lett 42(23):10,150–10,159. 10.1002/2015GL066683

[CR7] Archer MO, Hietala H, Hartinger MD, Plaschke F, Angelopoulos V (2019) Direct observations of a surface eigenmode of the dayside magnetopause. Nat Commun 10(1):615. 10.1038/s41467-018-08134-5 30755606 10.1038/s41467-018-08134-5 PMC6372605

[CR8] Baker GJ (2003) A comprehensive survey of auroral latitude Pc5 pulsation characteristics. J Geophys Res 108(A10):1384. 10.1029/2002JA009801

[CR9] Baker KB, Wing S (1989) A new magnetic coordinate system for conjugate studies at high latitudes. J Geophys Res 94(A7):9139–9143. 10.1029/JA094iA07p09139

[CR10] Baumjohann W, Junginger H, Haerendel G, Bauer OH (1984) Resonant Alfvén waves excited by a sudden impulse. J Geophys Res 89(A5):2765–2769. 10.1029/JA089iA05p02765

[CR11] Birch MJ, Hargreaves JK (2020) Quasi-periodic ripples in high latitude electron content, the geomagnetic field, and the solar wind. Sci Rep 10:1313. 10.1038/s41598-019-57201-4 31992733 10.1038/s41598-019-57201-4 PMC6987239

[CR12] Boulet (1992) Supplement: 1992 spring meeting, May 12-16. Eos Trans AGU 73(14):157. 10.1029/91EO10131

[CR13] Bruno R, Carbone V (2013) The solar wind as a turbulence laboratory. Living Rev Sol Phys 10(1):2. 10.12942/lrsp-2013-2

[CR14] Chen L, Hasegawa A (1974) A theory of long-period magnetic pulsations: 2. Impulse excitation of surface eigenmode. J Geophys Res 79(7):1033–1037. 10.1029/JA079i007p01033

[CR15] Chisham G, Orr D (1997) A statistical study of the local time asymmetry of Pc 5 ULF wave characteristics observed at midlatitudes by SAMNET. J Geophys Res 102(A11):24339–24350. 10.1029/97JA01801

[CR16] Claudepierre SG, Hudson MK, Lotko W, Lyon JG, Denton RE (2010) Solar wind driving of magnetospheric ULF waves: field line resonances driven by dynamic pressure fluctuations. J Geophys Res 115(A11):11202. 10.1029/2010JA015399

[CR17] Colonico A, Di Matteo S, Villante U (2020) Characterization of the Pc5 frequency range power spectrum at low latitude in 22 years of geomagnetic field observations. In: EGU General Assembly Conference Abstracts, p 4516. 10.5194/egusphere-egu2020-4516

[CR18] De Lauretis M, Regi M, Francia P, Marcucci MF, Amata E, Pallocchia G (2016) Solar wind-driven Pc5 waves observed at a polar cap station and in the near cusp ionosphere. J Geophys Res 121(11):11145–11156. 10.1002/2016JA023477

[CR19] Degeling AW, Rae IJ, Watt CEJ, Shi QQ, Rankin R, Zong Q-G (2018) Control of ULF wave accessibility to the inner magnetosphere by the convection of plasma density. J Geophys Res 123(2):1086–1099. 10.1002/2017JA024874

[CR20] Denton RE, Thomsen MF, Takahashi K, Anderson RR, Singer HJ (2011) Solar cycle dependence of bulk ion composition at geosynchronous orbit. J Geophys Res Space Phys 116(A3):03212. 10.1029/2010JA016027

[CR21] Di Matteo S, Sivadas N (2022) Solar-wind/magnetosphere coupling: understand uncertainties in upstream conditions. Front Astron Space Sci 9:333. 10.3389/fspas.2022.1060072

[CR22] Di Matteo S, Villante U (2017) The identification of solar wind waves at discrete frequencies and the role of the spectral analysis techniques: the identification of solar wind waves. J Geophys Res 122(5):4905–4920. 10.1002/2017JA023936

[CR23] Di Matteo S, Villante U (2018) The identification of waves at discrete frequencies at the geostationary orbit: the role of the data analysis techniques and the comparison with solar wind observations. J Geophys Res. 10.1002/2017JA024922

[CR24] Di Matteo S, Villante U, Viall N, Kepko L, Wallace S (2022) On differentiating multiple types of ULF magnetospheric waves in response to solar wind periodic density structures. J Geophys Res 127(3):e2021JA030144. 10.1029/2021JA030144 10.1029/2021JA030144 PMC9285707 35859722

[CR25] Dungey JW (1954) The attenuation of Alfvén waves. J Geophys Res 59(3):323–328. 10.1029/JZ059i003p00323

[CR26] Dungey JW (1955) Electrodynamics of the outer atmosphere. In: Physics of the ionosphere, Physical Society, London, p 229

[CR27] Elkington SR (2006) A review of ULF interactions with radiation belt electrons. In: Takahashi K et al. (eds) Magnetospheric ULF waves: synthesis and new directions. Geophysical Monograph, vol 169. AGU, Washington, p 177. 10.1029/169GM12

[CR28] Elkington SR, Sarris TE (2016) 4. In: Balasis G, Daglis IA, Mann IR (eds) The role of Pc-5 ULF waves in the radiation belts: current understanding and open questions. Oxford University Press, New York. 10.1093/acprof:oso/9780198705246.003.0005

[CR29] Eriksson PTI, Blomberg LG, Schaefer S, Glassmeier K-H (2006a) On the excitation of ULF waves by solar wind pressure enhancements. Ann Geophys 24(11):3161–3172. 10.5194/angeo-24-3161-2006

[CR30] Eriksson PTI, Walker ADM, Stephenson JAE (2006b) A statistical correlation of Pc5 pulsations and solar wind pressure oscillations. Adv Space Res 38(8):1763–1771. 10.1016/j.asr.2005.08.023

[CR31] Erlandson RE, Sibeck DG, Lopez RE, Zanetti LJ, Potemra TA (1991) Observations of solar wind pressure initiated fast mode waves at geostationary orbit and in the polar cap. J Atmos Sol-Terr Phys 53(3–4):231–239. 10.1016/0021-9169(91)90107-I

[CR32] Fenrich FR, Waters CL (2008) Phase coherence analysis of a field line resonance and solar wind oscillation. Geophys Res Lett 35(20):20102. 10.1029/2008GL035430

[CR33] Fenrich FR, Samson JC, Sofko G, Greenwald RA (1995) ULF high- and low- m field line resonances observed with the super dual auroral radar network. J Geophys Res 100(A11):21535–21547. 10.1029/95JA02024

[CR34] Francia P, Villante U (1997) Some evidence of ground power enhancements at frequencies of global magnetospheric modes at low latitude. Ann Geophys 15(1):17–23. 10.1007/s00585-997-0017-2

[CR35] Francia P, Lepidi S, Villante U, Di Giuseppe P, Lazarus AJ (1999) Geomagnetic response at low latitude to continuous solar wind pressure variations during northward interplanetary magnetic field. J Geophys Res 104(A9):19923–19930. 10.1029/1999JA900229

[CR36] Fukunishi H (1979) Latitude dependence of power spectra of magnetic pulsations near L = 4 excited by scc’s and si’s. J Geophys Res 84(A12):7191–7200. 10.1029/JA084iA12p07191

[CR37] Glassmeier K-H, Buchert S, Motschmann U, Korth A, Pedersen A (1999) Concerning the generation of geomagnetic giant pulsations by drift-bounce resonance ring current instabilities. Ann Geophys 17(3):338–350. 10.1007/s00585-999-0338-4

[CR38] Grant AK, Rosner JL (1992) Dipole transition matrix elements for systems with power-law potentials. Phys Rev D 46(9):3862–3870. 10.1103/PhysRevD.46.3862 10.1103/physrevd.46.3862 10015343

[CR39] Harrold BG, Samson JC (1992) Standing ULF modes of the magnetosphere: a theory. Geophys Res Lett 19(18):1811–1814. 10.1029/92GL01802

[CR40] Hartinger MD, Angelopoulos V, Moldwin MB, Takahashi K, Clausen LBN (2013) Statistical study of global modes outside the plasmasphere. J Geophys Res 118(2):804–822. 10.1002/jgra.50140

[CR41] Hartinger MD, Plaschke F, Archer MO, Welling DT, Moldwin MB, Ridley A (2015) The global structure and time evolution of dayside magnetopause surface eigenmodes. Geophys Res Lett 42(8):2594–2602. 10.1002/2015GL063623

[CR42] Hartinger MD, Elsden T, Archer MO, Takahashi K, Wright AN, Artemyev A, Zhang X, Angelopoulos V (2023a) Properties of magnetohydrodynamic normal modes in the Earth’s magnetosphere. J Geophys Res 128(12):e2023JA031987. 10.1029/2023JA031987

[CR43] Hartinger MD, Shi X, Rodger CJ, Fujii I, Rigler EJ, Kappler K, Matzka J, Love JJ, Baker JBH, Mac Manus DH, Dalzell M, Petersen T (2023b) Determining ULF wave contributions to geomagnetically induced currents: the important role of sampling rate. Space Weather 21(5):e2022SW003340. 10.1029/2022SW003340

[CR44] He F, Guo R-L, Dunn WR, Yao Z-H, Zhang H-S, Hao Y-X, Shi Q-Q, Rong Z-J, Liu J, Tian A-M, Zhang X-X, Wei Y, Zhang Y-L, Zong Q-G, Pu Z-Y, Wan W-X (2020) Plasmapause surface wave oscillates the magnetosphere and diffuse aurora. Nat Commun 11(1):1668. 10.1038/s41467-020-15506-3 32245960 10.1038/s41467-020-15506-3 PMC7125146

[CR45] Heyns MJ, Lotz SI, Gaunt CT (2021) Geomagnetic pulsations driving geomagnetically induced currents. Space Weather 19(2):e2020SW002557. 10.1029/2020SW002557

[CR46] Hughes WJ, Southwood DJ (1976) An illustration of modification of geomagnetic pulsation structure by the ionosphere. J Geophys Res 81(19):3241. 10.1029/JA081i019p03241

[CR47] Katsavrias C, Preka-Papadema P, Moussas X (2012) Wavelet analysis on solar wind parameters and geomagnetic indices. Sol Phys 280(2):623–640. 10.1007/s11207-012-0078-6

[CR48] Kaufmann RL, Walker DN (1974) Hydromagnetic waves excited during an ssc. J Geophys Res 79(34):5187–5195. 10.1029/JA079i034p05187

[CR49] Keiling A (2009) Alfvén waves and their roles in the dynamics of the Earth’s magnetotail: a review. Space Sci Rev 142(1–4):73–156. 10.1007/s11214-008-9463-8

[CR50] Kepko L, Spence HE (2003) Observations of discrete, global magnetospheric oscillations directly driven by solar wind density variations. J Geophys Res 108(A6):1257. 10.1029/2002JA009676

[CR51] Kepko L, Spence HE, Singer HJ (2002) ULF waves in the solar wind as direct drivers of magnetospheric pulsations. Geophys Res Lett 29(8):39–1394. 10.1029/2001GL014405

[CR52] Kepko L, Viall NM, Wolfinger K (2020) Inherent length scales of periodic mesoscale density structures in the solar wind over two solar cycles. J Geophys Res 125(8):28037. 10.1029/2020JA028037

[CR53] Kepko L, Viall NM, DiMatteo S (2024) Periodic mesoscale density structures comprise a significant fraction of the solar wind and are formed at the sun. J Geophys Res 129(1):e2023JA031403. 10.1029/2023JA031403

[CR54] Kivelson MG, Southwood DJ (1985) Resonant ULF waves: a new interpretation. Geophys Res Lett 12(1):49–52. 10.1029/GL012i001p00049

[CR55] Kivelson MG, Southwood DJ (1986) Coupling of global magnetospheric MHD eigenmodes to field line resonances. J Geophys Res 91(A4):4345. 10.1029/JA091iA04p04345

[CR56] Kivelson MG, Etcheto J, Trotignon JG (1984) Global compressibional oscillations of the terrestrial magnetosphere: the evidence and a model. J Geophys Res 89(A11):9851–9856. 10.1029/JA089iA11p09851

[CR57] Klibanova YY, Mishin VV, Tsegmed B, Moiseev AV (2016) Properties of daytime long-period pulsations during magnetospheric storm commencement. Geomagn Aeron 56(4):426–440. 10.1134/S0016793216040071

[CR58] Kokubun S (2013) ULF waves in the outer magnetosphere: geotail observation 1 transverse waves. Earth Planets Space 65(5):411–433. 10.5047/eps.2012.12.013

[CR59] Kozyreva O, Pilipenko V, Lorentzen D, Baddeley L, Hartinger M (2019) Transient oscillations near the dayside open-closed boundary: evidence of magnetopause surface mode? J Geophys Res 124(11):9058–9074. 10.1029/2018JA025684

[CR60] Kozyreva OV, Pilipenko VA, Bland EC, Baddeley LJ, Zakharov VI (2020) Periodic modulation of the upper ionosphere by ULF waves as observed simultaneously by SuperDARN radars and GPS/TEC technique. J Geophys Res 125(7):28032. 10.1029/2020JA028032

[CR61] Kruskal M, Schwarzschild M (1954) Some instabilities of a completely ionized plasma. Proc R Soc A 223(1154):348–360. 10.1098/rspa.1954.0120

[CR62] Lanzerotti LJ, Southwood DJ (1979) Hydromagnetic waves. In: Parker EN, Kennel CF, Lanzerotti LJ (eds) Solar System plasma physics, vol 3. North-Holland, Amsterdam, pp 109–135

[CR63] Lee EA, Mann IR, Loto’aniu TM, Dent ZC (2007) Global Pc5 pulsations observed at unusually low L during the great magnetic storm of 24 March 1991. J Geophys Res 112(A5):A05208. 10.1029/2006JA011872

[CR64] Lepidi S, Francia P, Villante U, Meloni A, Lazarus AJ, Lepping RP (1999) The Earth’s passage of the April 11, 1997 coronal ejecta: geomagnetic field fluctuations at high and low latitude during northward interplanetary magnetic field conditions. Ann Geophys 17(10):1245–1250. 10.1007/s00585-999-1245-4

[CR65] Lessard MR, Hudson MK, Samson JC, Wygant JR (1999) Simultaneous satellite and ground-based observations of a discretely driven field line resonance. J Geophys Res 104(A6):12361–12377. 10.1029/1998JA900117

[CR66] Lessard MR, Hanna J, Donovan EF, Reeves GD (2003) Evidence for a discrete spectrum of persistent magnetospheric fluctuations below 1 mHz. J Geophys Res 108(A3):1125. 10.1029/2002JA009311

[CR67] Liou K, Takahashi K, Newell PT, Yumoto K (2008) Polar ultraviolet imager observations of solar wind-driven ULF auroral pulsations. Geophys Res Lett 35(16):16101. 10.1029/2008GL034953

[CR68] Liu W, Sarris TE, Li X, Ergun R, Angelopoulos V, Bonnell J, Glassmeier KH (2010) Solar wind influence on Pc4 and Pc5 ULF wave activity in the inner magnetosphere. J Geophys Res 115(A12):12201. 10.1029/2010JA015299

[CR69] Mathie RA, Mann IR (2000) Observations of an anomalously low frequency Alfvén continuum in an abnormally expanded magnetosphere. Geophys Res Lett 27(24):4017–4020. 10.1029/2000GL003791

[CR70] Mathie RA, Mann IR, Menk FW, Orr D (1999b) Pc5 ULF pulsations associated with waveguide modes observed with the IMAGE magnetometer array. J Geophys Res 104(A4):7025–7036. 10.1029/1998JA900150

[CR71] Mathie RA, Menk FW, Mann IR, Orr D (1999a) Discrete field line resonances and the Alfvén continuum in the outer magnetosphere. Geophys Res Lett 26(6):659–662. 10.1029/1999GL900104

[CR72] McIlwain CE (1966) Magnetic coordinates. Space Sci Rev 5(5):585–598. 10.1007/BF00167327

[CR73] Menk FW (2011) Magnetospheric ULF waves: a review. In: Liu W, Fujimoto M (eds) The dynamic magnetosphere. IAGA Special Sopron Book Series, vol 3. Springer, Dordrecht, pp 223–256. 10.1007/978-94-007-0501-2_13

[CR74] Morton RJ, Tomczyk S, Pinto RF (2016) A global view of velocity fluctuations in the corona below 1.3 R_⊙_ with CoMP. Astrophys J 828(2):89. 10.3847/0004-637X/828/2/89

[CR75] Morton RJ, Weberg MJ, McLaughlin JA (2019) A basal contribution from p-modes to the Alfvénic wave flux in the Sun’s corona. Nat Astron 3:223. 10.1038/s41550-018-0668-9

[CR76] Motoba T, Kikuchi T, Lühr H, Tachihara H, Kitamura T-I, Hayashi K, Okuzawa T (2002) Global Pc5 caused by a DP 2-type ionospheric current system. J Geophys Res 107(A2):1032. 10.1029/2001JA900156

[CR77] Motoba T, Kikuchi T, Okuzawa T, Yumoto K (2003) Dynamical response of the magnetosphere-ionosphere system to a solar wind dynamic pressure oscillation. J Geophys Res 108(A5):1206. 10.1029/2002JA009696

[CR78] Mthembu SH, Malinga SB, Walker ADM, Magnus L (2009) Characterization of ultra low frequency (ULF) pulsations and the investigation of their possible source. Ann Geophys 27(8):3287–3296. 10.5194/angeo-27-3287-2009

[CR79] Norouzi-Sedeh L, Waters CL, Menk FW (2015) Survey of ULF wave signatures seen in the Tasman international geospace environment radars data. J Geophys Res 120(2):949–963. 10.1002/2014JA020652

[CR80] Nykyri K, Di Matteo S, Archer MO, Ma X, Hartinger MD, Sarantos M, Zesta E, Paterson WR (2024) Could a low-frequency perturbation in the Earth’s magnetotail be generated by the lunar wake? Geophys Res Lett 51(22):e2024GL110129. 10.1029/2024GL110129

[CR81] Ozeke LG, Mann IR, Murphy KR, Rae IJ, Milling DK, Elkington SR, Chan AA, Singer HJ (2012) ULF wave derived radiation belt radial diffusion coefficients. J Geophys Res 117(A4):A04222. 10.1029/2011JA017463

[CR82] Ozeke LG, Mann IR, Murphy KR, Jonathan Rae I, Milling DK (2014) Analytic expressions for ULF wave radiation belt radial diffusion coefficients. J Geophys Res 119(3):1587–1605. 10.1002/2013JA019204 10.1002/2013JA019204 PMC4497482 26167440

[CR83] Plaschke F, Glassmeier K-H, Auster HU, Angelopoulos V, Constantinescu OD, Fornaçon K-H, Georgescu E, Magnes W, McFadden JP, Nakamura R (2009a) Statistical study of the magnetopause motion: first results from THEMIS. J Geophys Res 114(A1):A00C10. 10.1029/2008JA013423

[CR84] Plaschke F, Glassmeier K-H, Auster HU, Constantinescu OD, Magnes W, Angelopoulos V, Sibeck DG, McFadden JP (2009b) Standing Alfvén waves at the magnetopause. Geophys Res Lett 36(2):L02104. 10.1029/2008GL036411

[CR85] Plaschke F, Glassmeier K-H, Sibeck DG, Auster HU, Constantinescu OD, Angelopoulos V, Magnes W (2009c) Magnetopause surface oscillation frequencies at different solar wind conditions. Ann Geophys 27(12):4521–4532. 10.5194/angeo-27-4521-2009

[CR86] Pokhotelov D, Rae IJ, Murphy KR, Mann IR (2015) The influence of solar wind variability on magnetospheric ULF wave power. Ann Geophys 33(6):697–701. 10.5194/angeo-33-697-2015

[CR87] Potemra TA, Lühr H, Zanetti LJ, Takahashi K, Erlandson RE, Marklund GT, Block LP, Blomberg LG, Lepping RP (1989) Multisatellite and ground-based observations of transient ULF waves. J Geophys Res 94(A3):2543. 10.1029/JA094iA03p02543

[CR88] Prikryl P, Greenwald RA, Sofko GJ, Villain JP, Ziesolleck CWS, Friis-Christensen E (1998) Solar-wind-driven pulsed magnetic reconnection at the dayside magnetopause, Pc5 compressional oscillations, and field line resonances. J Geophys Res 103(A8):17307–17322. 10.1029/97JA03595

[CR89] Provan G, Yeoman TK (1997) A comparison of field-line resonances observed at the Goose Bay and Wick radars. Ann Geophys 15(2):231–235. 10.1007/s00585-997-0231-y

[CR90] Pu Z-Y, Kivelson MG (1983) Kelvin-Helmholtz instability at the magnetopause: solution for compressible plasmas. J Geophys Res 88(A2):841–852. 10.1029/JA088iA02p00841

[CR91] Radoski HR (1974) A theory of latitude dependent geomagnetic micropulsations: the asymptotic fields. J Geophys Res 79(4):595–603. 10.1029/JA079i004p00595

[CR92] Regi M, Del Corpo A, De Lauretis M (2017) The use of the empirical mode decomposition for the identification of mean field aligned reference frames. Ann Geophys 56:0651. 10.4401/ag-7067

[CR93] Rostoker G, Samson JC, Creutzberg F, Hughes TJ, McDiarmid DR, McNamara AG, Jones AV, Wallis DD, Cogger LL (1995) Canopus — a ground-based instrument array for remote sensing the high latitude ionosphere during the ISTP/GGS program. Space Sci Rev 71(1–4):743–760. 10.1007/BF00751349

[CR94] Rubtsov AV, Nosé M, Matsuoka A, Kasahara Y, Kumamoto A, Tsuchiya F, Shinohara I, Miyoshi Y (2023) Plasmasphere control of ULF wave distribution at different geomagnetic conditions. J Geophys Res 128(10):e2023JA031675. 10.1029/2023JA031675

[CR95] Ruohoniemi JM, Greenwald RA, Baker KB, Samson JC (1991) HF radar observations of Pc 5 field line resonances in the midnight/early morning MLT sector. J Geophys Res 96(A9):15697–15710. 10.1029/91JA00795

[CR96] Saito T (1969) Geomagnetic pulsations. Space Sci Rev 10(3):319–412. 10.1007/BF00203620

[CR97] Saito T, Matsushita S (1967) Geomagnetic pulsations associated with sudden commencements and sudden impulses. Planet Space Sci 15:573. 10.1016/0032-0633(67)90163-8

[CR98] Samson JC, Rankin R (1994) The coupling of solar wind energy to MHD cavity modes, waveguide modes, and field line resonances in the Earth’s magnetosphere. In: Engebretson MJ, Takahashi K, Scholer M (eds) Solar wind sources of magnetospheric ultra-low-frequency waves. Geophysical Monograph, vol 169. AGU, Washington, pp 253–264. 10.1029/GM081p0253

[CR99] Samson JC, Greenwald RA, Ruohoniemi JM, Hughes TJ, Wallis DD (1991a) Magnetometer and radar observations of magnetohydrodynamic cavity modes in the Earth’s magnetosphere. Can J Phys 69(8–9):929–937. 10.1139/p91-147

[CR100] Samson JC, Hughes TJ, Creutzberg F, Wallis DD, Greenwald RA, Ruohoniemi JM (1991b) Observations of a detached, discrete arc in association with field line resonances. J Geophys Res 96(A9):15683–15695. 10.1029/91JA00796

[CR101] Samson JC, Harrold BG, Ruohoniemi JM, Greenwald RA, Walker ADM (1992a) Field line resonances associated with MHD waveguides in the magnetosphere. Geophys Res Lett 19(5):441–444. 10.1029/92GL00116

[CR102] Samson JC, Wallis DD, Hughes TJ, Creutzberg F, Ruohoniemi JM, Greenwald RA (1992b) Substorm intensifications and field line resonances in the nightside magnetosphere. J Geophys Res 97(A6):8495. 10.1029/91JA03156

[CR103] Shepherd SG (2014) Altitude-adjusted corrected geomagnetic coordinates: definition and functional approximations. J Geophys Res 119(9):7501–7521. 10.1002/2014JA020264

[CR104] Shi X, Ruohoniemi JM, Baker JBH, Lin D, Bland EC, Hartinger MD, Scales WA (2018) Survey of ionospheric Pc3-5 ULF wave signatures in SuperDARN high time resolution data. J Geophys Res 123(5):4215–4231. 10.1029/2017JA025033 10.1029/2017JA025033 PMC6013742 29938156

[CR105] Shimazu H, Araki T, Kamei T, Hanado H (1995) A symmetric appearance of Pc5 on dawn and dusk sides associated with solar wind pressure enhancement. J Geomagn Geoelectr 47(2):177–189. 10.5636/jgg.47.177

[CR106] Sibeck DG, Baumjohann W, Elphic RC, Fairfield DH, Fennell JF, Gail WB, Lanzerotti LJ, Lopez RE, Luehr H, Lui ATY, Maclennan CG, McEntire RW, Potemra TA, Rosenberg TJ, Takahashi K (1989) The magnetospheric response to 8-minute period strong-amplitude upstream pressure variations. J Geophys Res 94(A3):2505. 10.1029/JA094iA03p02505

[CR107] SILSO, World Data Center, 1978-2018: Sunspot Number and Long-term Solar Observations. In: Royal Observatory of Belgium, on-line Sunspot Number Catalogue. http://www.sidc.be/silso/

[CR108] Southwood DJ (1974) Some features of field line resonances in the magnetosphere. Planet Space Sci 22(3):483–491. 10.1016/0032-0633(74)90078-6

[CR109] Stephenson JAE, Walker ADM (2002) HF radar observations of Pc5 ULF pulsations driven by the solar wind. Geophys Res Lett 29(9):8–184. 10.1029/2001GL014291

[CR110] Stephenson JAE, Walker ADM (2010) Coherence between radar observations of magnetospheric field line resonances and discrete oscillations in the solar wind. Ann Geophys 28(1):47–59. 10.5194/angeo-28-47-2010

[CR111] Stewart B (1861) XXII. On the great magnetic disturbance which extended from August 28 to September 7, 1859, as recorded by photography at the kew observatory. Philos Trans R Soc 151:423–430. 10.1098/rstl.1861.0023

[CR112] Takahashi K, Ukhorskiy AY (2007) Solar wind control of Pc5 pulsation power at geosynchronous orbit. J Geophys Res 112(A11):11205. 10.1029/2007JA012483

[CR113] Tomczyk S, McIntosh SW, Keil SL, Judge PG, Schad T, Seeley DH, Edmondson J (2007) Alfvén waves in the solar corona. Science 317(5842):1192. 10.1126/science.1143304 17761876 10.1126/science.1143304

[CR114] Treumann RA, Baumjohann W, Narita Y (2019) On the ion-inertial-range density-power spectra in solar wind turbulence. Ann Geophys 37(2):183–199. 10.5194/angeo-37-183-2019. arXiv:1811.09995 [physics.space-ph]

[CR115] Vellante M, Villante U, De Lauretis M, Barchi G (1996) Solar cycle variation of the dominant frequencies of Pc3 geomagnetic pulsations at L = 1.6. Geophys Res Lett 23(12):1505–1508. 10.1029/96GL01399

[CR116] Vellante M, Förster M, Villante U, Zhang TL, Magnes W (2007) Solar activity dependence of geomagnetic field line resonance frequencies at low latitudes. J Geophys Res 112(A2):02205. 10.1029/2006JA011909

[CR117] Viall NM, Kepko L, Spence HE (2008) Inherent length-scales of periodic solar wind number density structures. J Geophys Res 113(A7):07101. 10.1029/2007JA012881

[CR118] Viall NM, Kepko L, Spence HE (2009) Relative occurrence rates and connection of discrete frequency oscillations in the solar wind density and dayside magnetosphere. J Geophys Res 114(A1):A01201. 10.1029/2008JA013334

[CR119] Villante U (2007) Ultra low frequency waves in the magnetosphere. In: Kamide Y, Chian AC-L (eds) Handbook of the solar-terrestrial environment, p 398. 10.1007/978-3-540-46315-3_16

[CR120] Villante U, Lepidi S, Francia P, Meloni A, Palangio P (1997) Long period geomagnetic field fluctuations at Terra Nova Bay (Antarctica). Geophys Res Lett 24(12):1443–1446. 10.1029/97GL01466

[CR121] Villante U, Francia P, Lepidi S, De Lauretis M, Pietropaolo E, Cafarella L, Meloni A, Lazarus AJ, Lepping RP, Mariani F (1998) Geomagnetic field variations at low and high latitude during the January 10-11, 1997 magnetic cloud. Geophys Res Lett 25(14):2593–2596. 10.1029/98GL00335

[CR122] Villante U, Francia P, Lepidi S (2001) Pc5 geomagnetic field fluctuations at discrete frequencies at a low latitude station. Ann Geophys 19(3):321–325. 10.5194/angeo-19-321-2001

[CR123] Villante U, Vellante M, Francia P, De Lauretis M, Meloni A, Palangio P, Zolesi B, Pezzopane M, Förster M, Zhang TL, Magnes W, Nenovski P, Cholakov I, Wesztergom V (2006) ULF fluctuations of the geomagnetic field and ionospheric sounding measurements at low latitudes during the first CAWSES campaign. Ann Geophys 24(5):1455–1468. 10.5194/angeo-24-1455-2006

[CR124] Villante U, Francia P, Vellante M, di Giuseppe P, Nubile A, Piersanti M (2007) Long-period oscillations at discrete frequencies: a comparative analysis of ground, magnetospheric, and interplanetary observations. J Geophys Res 112(A4):04210. 10.1029/2006JA011896

[CR125] Villante U, Del Corpo A, Francia P (2013) Geomagnetic and solar wind fluctuations at discrete frequencies: a case study. J Geophys Res 118(1):218–231. 10.1029/2012JA017971

[CR126] Villante U, Di Matteo S, Piersanti M (2016) On the transmission of waves at discrete frequencies from the solar wind to the magnetosphere and ground: a case study. J Geophys Res 121(1):380–396. 10.1002/2015JA021628

[CR127] Villante U, Recchiuti D, Di Matteo S (2022) The transmission of ULF waves from the solar wind to the magnetosphere: an analysis of some critical aspects. Front Astron Space Sci 9:835539. 10.3389/fspas.2022.835539

[CR128] Voelker H (1966) On geomagnetic pulsations accompanying storm sudden commencements and sudden impulses. Earth Planet Sci Lett 1(6):383–386. 10.1016/0012-821X(66)90031-8

[CR129] Walker ADM, Ruohoniemi JM, Baker KB, Greenwald RA, Samson JC (1992) Spatial and temporal behavior of ULF pulsations observed by the Goose Bay HF radar. J Geophys Res 97(A8):12187. 10.1029/92JA00329

[CR130] Wang B, Liu T, Nishimura Y, Zhang H, Hartinger M, Shi X, Ma Q, Angelopoulos V, Frey HU (2020) Global propagation of magnetospheric Pc5 ULF waves driven by foreshock transients. J Geophys Res 125(12):e2020JA028411. 10.1029/2020JA028411

[CR131] Waters CL (2000) ULF resonance structure in the magnetosphere. Adv Space Res 25(7–8):1541–1558. 10.1016/S0273-1177(99)00667-5

[CR132] Waters CL, Samson JC, Donovan EF (1995) The temporal variation of the frequency of high latitude field line resonances. J Geophys Res 100(A5):7987. 10.1029/94JA02712

[CR133] Yagova NV (2015) Spectral slope of high-latitude geomagnetic disturbances in the frequency range 1-5 mHz. Control parameters inside and outside the magnetosphere. Geomagn Aeron 55(1):32–40. 10.1134/S0016793215010144

[CR134] Zhang XY, Zong Q-G, Wang YF, Zhang H, Xie L, Fu SY, Yuan CJ, Yue C, Yang B, Pu ZY (2010) ULF waves excited by negative/positive solar wind dynamic pressure impulses at geosynchronous orbit. J Geophys Res 115(A10):10221. 10.1029/2009JA015016

[CR135] Ziesolleck CWS, McDiarmid DR (1994) Auroral latitude Pc 5 field line resonances: quantized frequencies, spatial characteristics, and diurnal variation. J Geophys Res 99(A4):5817. 10.1029/93JA02903

[CR136] Ziesolleck CWS, McDiarmid DR (1995) Statistical survey of auroral latitude Pc 5 spectral and polarization characteristics. J Geophys Res 100(A10):19299. 10.1029/95JA00434

[CR137] Ziesolleck CWS, Fenrich FR, Samson JC, McDiarmid DR (1998) Pc5 field line resonance frequencies and structure observed by SuperDARN and CANOPUS. J Geophys Res 103(A6):11771–11786. 10.1029/98JA00590

[CR138] Zong Q (2022) Magnetospheric response to solar wind forcing: ultra-low-frequency wave-particle interaction perspective. Ann Geophys 40(1):121–150. 10.5194/angeo-40-121-2022

[CR139] Zong Q-G, Zhou X-Z, Wang YF, Li X, Song P, Baker DN, Fritz TA, Daly PW, Dunlop M, Pedersen A (2009) Energetic electron response to ULF waves induced by interplanetary shocks in the outer radiation belt. J Geophys Res 114(A10):10204. 10.1029/2009JA014393

[CR140] Zong Q, Rankin R, Zhou X (2017) The interaction of ultra-low-frequency PC3-5 waves with charged particles in Earth’s magnetosphere. Rev Mod Plasma Phys 1(1):10. 10.1007/s41614-017-0011-4

